# The regional variation of laminar thickness in the human isocortex is related to cortical hierarchy and interregional connectivity

**DOI:** 10.1371/journal.pbio.3002365

**Published:** 2023-11-09

**Authors:** Amin Saberi, Casey Paquola, Konrad Wagstyl, Meike D. Hettwer, Boris C. Bernhardt, Simon B. Eickhoff, Sofie L. Valk

**Affiliations:** 1 Otto Hahn Research Group for Cognitive Neurogenetics, Max Planck Institute for Human Cognitive and Brain Sciences, Leipzig, Germany; 2 Institute of Neurosciences and Medicine (INM-7), Research Centre Jülich, Jülich, Germany; 3 Institute of Systems Neuroscience, Heinrich Heine University Düsseldorf, Düsseldorf, Germany; 4 Wellcome Trust Centre for Neuroimaging, University College London, London, United Kingdom; 5 Max Planck School of Cognition, Leipzig, Germany; 6 Multimodal Imaging and Connectome Analysis Laboratory, McConnell Brain Imaging Centre, Montreal Neurological Institute and Hospital, McGill University, Montreal, Canada; Inserm U1208, FRANCE

## Abstract

The human isocortex consists of tangentially organized layers with unique cytoarchitectural properties. These layers show spatial variations in thickness and cytoarchitecture across the neocortex, which is thought to support function through enabling targeted corticocortical connections. Here, leveraging maps of the 6 cortical layers based on 3D human brain histology, we aimed to quantitatively characterize the systematic covariation of laminar structure in the cortex and its functional consequences. After correcting for the effect of cortical curvature, we identified a spatial pattern of changes in laminar thickness covariance from lateral frontal to posterior occipital regions, which differentiated the dominance of infra- versus supragranular layer thickness. Corresponding to the laminar regularities of cortical connections along cortical hierarchy, the infragranular-dominant pattern of laminar thickness was associated with higher hierarchical positions of regions, mapped based on resting-state effective connectivity in humans and tract-tracing of structural connections in macaques. Moreover, we show that regions with similar laminar thickness patterns have a higher likelihood of structural connections and strength of functional connections. In sum, here we characterize the organization of laminar thickness in the human isocortex and its association with cortico-cortical connectivity, illustrating how laminar organization may provide a foundational principle of cortical function.

## Abstract 2

The human isocortex consists of tangentially organized layers with unique cytoarchitectural properties. This study shows that the regional variation of high-resolution histology-based laminar thickness profiles in the human isocortex is associated with the cortical connectome and hierarchical organization.

## Introduction

Cortical cytoarchitecture, that is, the organization and characteristics of neurons across the depth of the cerebral cortex, varies markedly across the cortical mantle [1–4]. Characterizing this variation has been an important focus of histological studies over the past century. Early studies were largely based on visual inspection and qualitative descriptions of cytoarchitectural features across the cerebral cortex to identify local borders between regions [2] or to describe more global cytoarchitectural variations [4,5]. With methodological advances of recent decades, there has been a shift towards more quantitative investigations of cortical cytoarchitecture based on statistical analysis on 2D histological sections [6–8]. The central idea of these studies has been to quantify the variation of cell body–stained image intensity across the cortical depth, i.e., "cortical profile." This is followed by observer-independent analysis of how cortical profiles vary across the cerebral cortex and define borders of regions, particularly with respect to the central moments, i.e., mean, standard deviation, kurtosis, and skewness [1,6,7]. The release of BigBrain, a whole-brain ultrahigh-resolution *postmortem* histological atlas of a 65-year-old male [9], enables such quantitative investigations at a much larger scale, for example, to quantify large-scale microstructural gradients at a neocortical [10] and mesiotemporal level [11].

Quantitative studies of cortical profiles have helped improve our understanding of cytoarchitectural variability of the human cerebral cortex. However, the cerebral cortex is a layered structure, and models of cortical profiles are, at least explicitly, agnostic to cortical layering. The layers in the neocortex are generally described as 6 horizontally superimposed stripes of gray matter with characteristic features such as size, type, and density of the neurons, which can again be differentiated into multiple sublayers [1,4]. From the pial to the gray-white matter interface, they include layer I, which contains mostly dendrites and axon terminals and has a low cellular density; layers II and III, which mainly contain pyramidal cells, with a size gradient in neurons of layer III that become larger towards its lower extent; layer IV, which consists of densely packed small pyramidal and non-pyramidal neurons; layer V, which is composed of pyramidal neurons that are small and intratelencephalic (layer Va) or large and sparse (layer Vb); and layer VI with corticothalamic pyramidal cells and heterogeneously shaped neurons [2,4,12,13]. One of the prominent cytoarchitectural features that vary across the cerebral cortex is its laminar structure, with respect to laminar thickness, as well as neuronal size and density of each layer. Indeed, laminar features have been an important focus of many qualitative studies of human cytoarchitectural variation [3,4,14]. For example, agranular and dysgranular cortical types are defined based on the absence or thinness of layer IV, relative to eulaminate and koniocortical regions [4,14]. However, studies on quantitative analysis of cortical cytoarchitecture with respect to its laminar features in humans are limited. Yet, understanding layered organization of the human neocortex may provide further insights into how intracortical circuits ultimately support function [15,16].

The laminar pattern and likelihood of cortico-cortical connections are suggested to relate to the interregional variation of cortical cytoarchitecture [3,17,18]. Connectivity is shown to be more likely between regions with similar cytoarchitecture [19–24]. In addition, the gradation of cytoarchitecture is suggested to predict the laminar pattern of cortico-cortical connections [3,17,18,25,26], categorized as "feedback" (FB), "feedforward" (FF), or "lateral" based on tract-tracing data [27–29]. These laminar projections have, in turn, been used to describe an ordering of regions along a cortical hierarchy, in which FF projections are suggested to carry high-dimensional sensory information from lower to higher regions and are reciprocated by FB projections transmitting context and modulatory signals from higher to lower regions [29,30]. Recently, it was shown that a marker of cortical myelination (T1w/T2w) was associated with the map of laminar-based hierarchy [27]. Together with findings on the association of cortical cytoarchitecture and laminar projections [3,17,25,26], this suggests a potential link between cortical microstructure and hierarchy. Yet, it is unclear how laminar thickness may scaffold connections within the cortical hierarchy. Notably, in neuroscience, the term "hierarchy" has been used to describe different phenomena [31], such as gradients of structural and functional features [27,32], topological sequence of connections [33], asymmetry of directional connections indicating interregional control or dominance [34,35], or, as described above, the sorting of laminar projection patterns and their physiological correlates [28,29,36–41]. Throughout this paper, we will focus on the latter 2 definitions of hierarchy, that is, laminar-based and asymmetry-based hierarchy.

Here, we aimed to study the organization of laminar profiles across the cortical mantle and its relevance to cortical hierarchy and interregional connectivity to further understand the relationship between human intracortical structure and function. To do so, we leveraged previously reported maps of the locations of cortical layers across isocortical regions of the BigBrain that were predicted using a convolutional neural network [42]. We extend previous work investigating the spatial arrangement of cortical profiles based on microstructure [10], through formally probing layer-profiles in this model, and describe a data-driven axis of laminar thickness covariance by quantifying the interregional covariation of laminar thickness in the BigBrain [9,42]. To do so, we employ dimensionality reduction techniques to identify the principal axis along which laminar thickness covaries. We next evaluate how laminar thickness covariation relates to hierarchical positioning of cortical regions based on resting-state effective connectivity in humans and anatomical layer-wise connections in macaques. We then investigate whether similarity of laminar structure relates to the likelihood and strength of structural and functional interregional connections and, last, explore its links to interregional structural covariance and maturational coupling.

## Results

### BigBrain laminar thickness covariance and its principal axis

**Fig 1:**
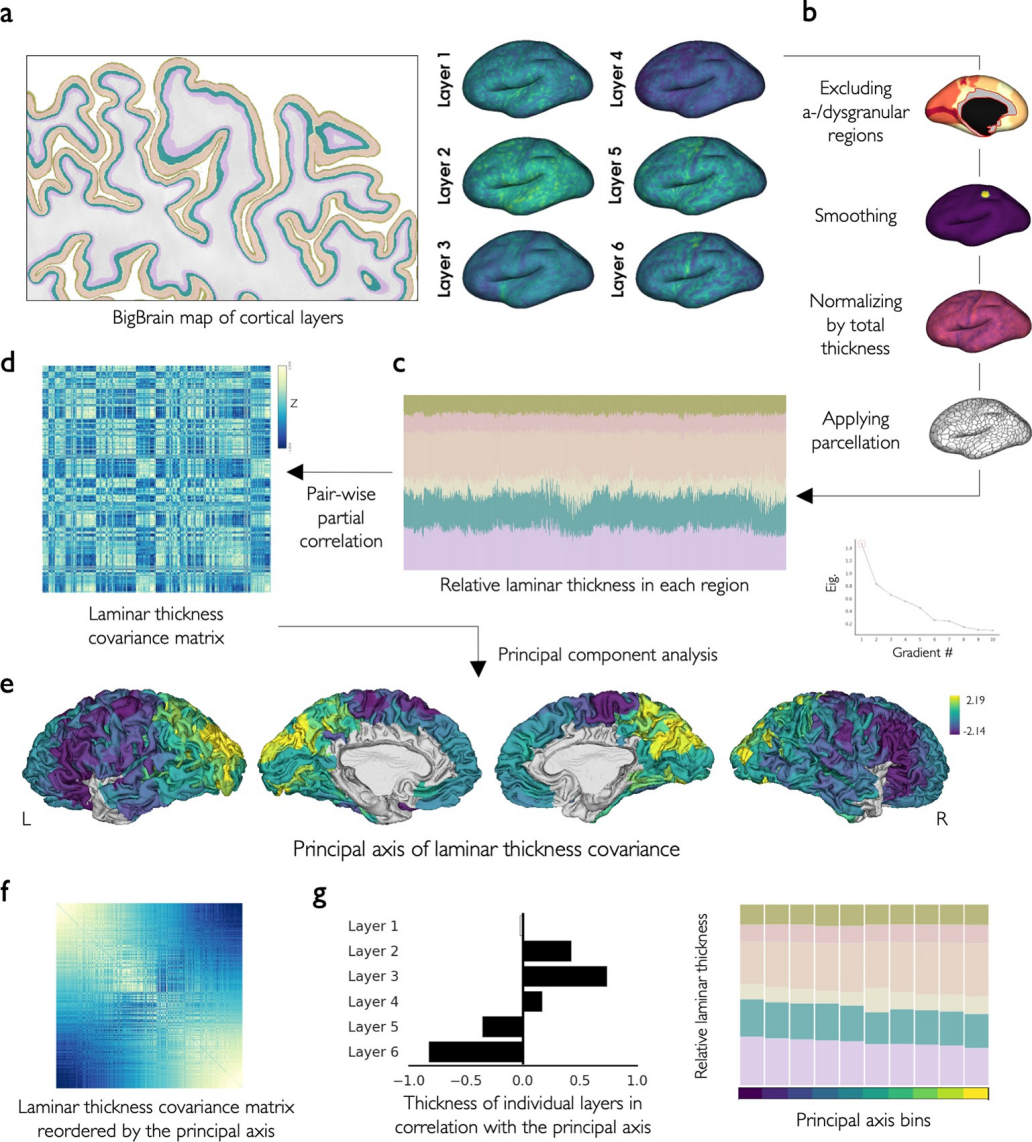
Laminar thickness covariance and its principal axis. **(a)** The laminar thickness maps based on the *postmortem* histological atlas of BigBrain. (**b**, **c**) For each cortical layer, the a-/dysgranular regions were excluded, the thickness map was smoothed using a disc, normalized by the total thickness, and parcellated. **(d)** The LTC matrix was created by calculating the pairwise partial correlation of relative thickness across layers and between regions. (**e)** The main axis of laminar thickness covariance (LTC G1) was calculated by principal component analysis. (**f)** LTC G1 reorders the LTC such that closer regions on this axis have similar LTC patterns. (**g)** LTC G1 characterized a shift of infra- to supragranular dominance. The data and code needed to generate this figure can be found in https://zenodo.org/record/8410965.

We used the maps of cortical layers based on the BigBrain, an ultrahigh-resolution *postmortem* histological atlas of a 65-year-old male [9,42], to study laminar thickness covariation across the cerebral cortex (Fig 1A). We first excluded agranular and dysgranular regions, such as cingulate, anterior insula, temporal pole, and parahippocampal cortices, in addition to allocortex, given their lack of a clear 6-layer structure [14]. Cortical folding impacts the laminar structure, such that layers inside of the fold are compressed and thicker, whereas layers outside of the fold are stretched and thinner [43–46]. Accordingly, in the BigBrain, we observed that from the sulci to the gyri, the relative thickness of superficial layers decreases (r = -0.28, p_spin_ < 0.001) (S1A Fig). To reduce the local effects of curvature on laminar thickness, we smoothed laminar thickness maps using a moving disk, which reduced this effect remarkably, as the correlation of curvature with the relative thickness of superficial layers dropped to r = -0.13 (p_spin_ < 0.001) (S1A). Following, the laminar thickness maps were normalized by the total cortical thickness at each cortical location to get the relative thickness. The maps of relative laminar thickness were then parcellated using the Schaefer-1000 parcellation (Fig 1B and 1C). We next calculated the laminar thickness covariance (LTC) matrix, showing the similarity of laminar thickness patterns between cortical areas. The LTC matrix was created by calculating the pairwise partial correlation of relative laminar thickness between cortical locations (controlled for the average laminar thickness across the isocortex), which was subsequently z-transformed (Figs 1D and S2).

Principal component analysis was then applied to the LTC matrix to identify the axes or gradients along which differences in the loadings indicates regional dissimilarity in the laminar thickness pattern [47]. Here, we focused on the principal axis, LTC G1, which explained approximately 28.1% of the variance in LTC (see the second and third axes in S3 Fig). LTC G1 spanned from the lateral frontal regions, towards medial frontal, temporal, and primary visual areas, ending in the parietal and occipital regions (Fig 1E). This axis was correlated with the relative thickness of layers II (r = 0.42, p_variogram_ < 0.001), III (r = 0.73, p_variogram_ < 0.001), and IV (r = 0.17, p_variogram_ < 0.001) positively, and layers V (r = -0.35, p_variogram_ < 0.001) and VI (r = -0.82, p_variogram_ < 0.001) negatively, characterizing a shift from the dominance of infra- to supragranular layers (Fig 1G).

The spatial map of LTC G1 was mostly robust to analytical choices, i.e., using unparcelled data (17,386 vertices) as well as alternative parcellation schemes, covariance metrics, dimensionality reduction techniques, sparsity ratios, and the inclusion of a-/dysgranular regions (S4 Fig). In addition, evaluating the left and right hemispheres separately, we observed high similarity of hemisphere-specific LTC G1 maps (r = 0.74, p_variogram_ < 0.001; S5 Fig). While LTC was higher between physically proximal regions in an exponential regression model (R^2^ = 0.16, p_spin_ = 0.004), the spatial map of LTC G1 was robust to the effects of geodesic distance (r = 0.97, p_variogram_ < 0.001) (S6 Fig). We also showed that LTC G1 created based on a 3-layer model with supragranular, granular, and infragranular layers was similar to the original 6-layer model (S7 Fig). Moreover, as an alternative data-driven approach of quantifying organization of laminar thickness variability, we used K-means clustering, which revealed 4 optimal clusters of the regional laminar thickness profiles that were largely aligned with the LTC G1 (F = 813.2, p_spin_ < 0.001) (S8 Fig).

Last, we quantified intraregional homogeneity of laminar thickness patterns as the difference of intra- versus interregional vertex-level LTC across Brodmann areas. We observed higher intraregional homogeneity of laminar thickness in areas such as BA17, BA45, and BA47, in contrast to a higher heterogeneity in areas such as BA22 and BA23 (S9 Fig).

### Laminar thickness covariation with laminar neuronal density and size

Having characterized the spatial variation of laminar thickness patterns, we next studied its association with layer-/depth-wise measures of neuronal density and size in the BigBrain, in addition to a map of cortical types, which is a theory-driven map of laminar structure. By doing so, we aimed to understand how laminar thickness covaries with the other cytoarchitectural features of laminar structure captured using data- and theory-driven approaches.

**Fig 2:**
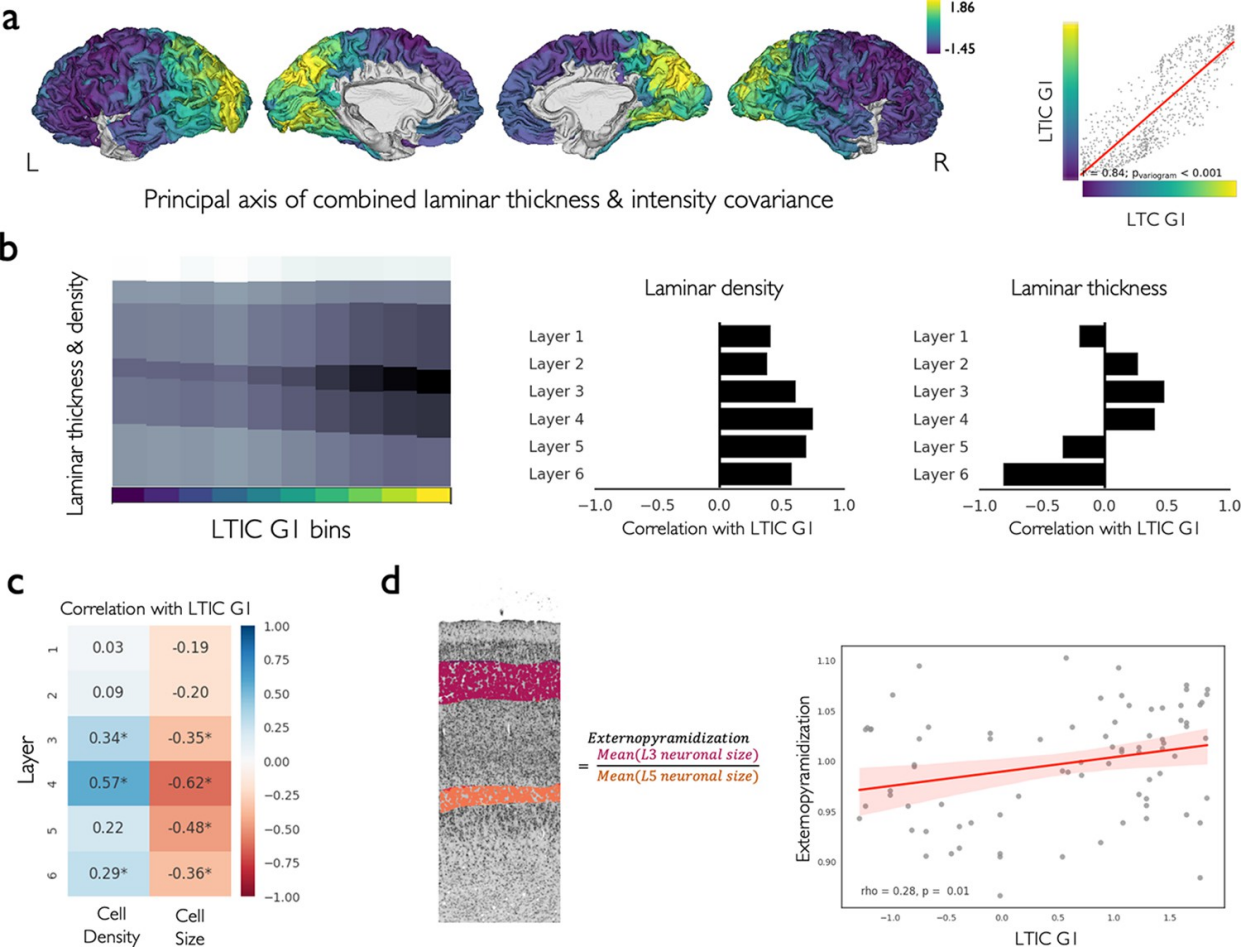
Laminar thickness covariation with laminar neuronal density and size. **(a)** The principal axis of combined laminar thickness and intensity covariance matrices (LTIC G1) and its correlation with LTC G1. (**b)** The pattern of changes in the thickness and density of the 6 layers along the LTIC G1. **(c)** The correlation of laminar neuronal density and size along the LTIC G1 among the available samples (S11 Fig). **(d)** The correlation of externopyramidization with the LTIC G1. The data and code needed to generate this figure can be found in https://zenodo.org/record/8410965.

Microstructural profile covariance (MPC) is based on the image intensity profiles in the BigBrain cerebral cortex, reflecting variation of grey-matter density across cortical depth, and is a data-driven model of cytoarchitecture that is explicitly agnostic to layer boundaries [10]. MPC was significantly correlated with our model of laminar thickness covariation, at the level of matrices (r = 0.34, p_spin_ < 0.001) and their principal axes (r = 0.55, p_variogram_ < 0.001) (S10 Fig). Extending this approach to the individual layers, we calculated layer-wise intensity profiles of the BigBrain cerebral cortex as the image intensity sampled at 10 equivolumetric surfaces across each layer’s depth, which we then averaged across the samples. Next, we calculated laminar intensity covariance (LIC) and applied principal component analysis on the fused matrices of LTC and LIC, as a model of laminar structure covariation that took both laminar thickness and laminar grey-matter density into account. The principal axis of the laminar thickness and intensity covariance (LTIC G1) was significantly correlated with LTC G1 and showed a similar pattern (r = 0.84, p_variogram_ < 0.001) (Fig 2A). Along the LTIC G1, from rostral to caudal regions, we observed significantly increased grey-matter density of all the layers with layer IV showing the strongest effect (r = 0.75, p_variogram_ < 0.001) (Fig 2B). The image intensity in the cell body–stained BigBrain atlas reflects an aggregate of neuronal size and density, and at a resolution of 20 μm, as individual neurons cannot be readily distinguished, these components cannot be disentangled. To further explore variations of neuronal size and density separately, we leveraged on a preliminary dataset of layer-wise neuron segmentations based on higher-resolution (1 μm) 2D patches from selected cortical regions of the BigBrain (S11A Fig). We observed variation of laminar neuronal features along LTIC G1, which was most prominent in layer IV, showing increase of neuronal density (rho = 0.57, p < 0.001) and decrease of neuronal size (rho = −0.62, *p* < 0.001) (Fig 2C). In addition, the ratio of average neuronal size in layer III to layer V, as a proxy for externopyramidization, was increased along LTIC G1 (rho = 0.28, *p* = 0.01; Fig 2D). Last, we compared our data-driven model of laminar thickness covariation with the map of cortical types, a theory-driven model of laminar structural variation [14], and observed no significant association of the maps (F = 6.41, p_spin_ = 0.633) but significantly higher within- than between-type average LTC in koniocortex (S12 Fig).

### Principal axis of laminar thickness covariance in association with cortical hierarchy

We next sought to understand how the variation of laminar thickness across the isocortex relates to the cortico-cortical directional connectivity and the resulting maps of asymmetry- and laminar-based cortical hierarchy.

**Fig 3:**
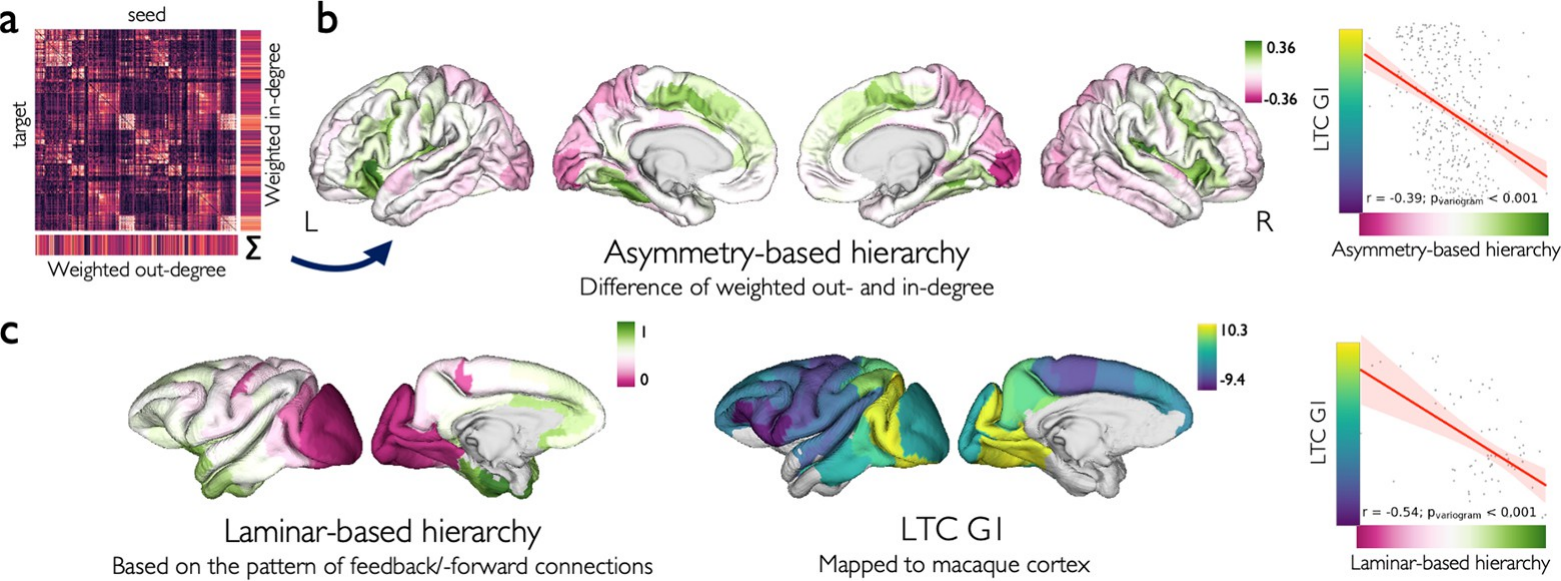
Association of laminar thickness covariance and cortical hierarchy. **(a)** The group-averaged effective connectivity matrix based on regression dynamic causal modeling. **(b)** Regional asymmetry-based hierarchy was calculated as the difference between their weighted unsigned out-degree and in-degree and was significantly correlated with the LTC G1. **(c)** Regional laminar-based hierarchy map of macaque (left hemisphere) was correlated with the LTC G1 aligned to the macaque cerebral cortex. The data and code needed to generate this figure can be found in https://zenodo.org/record/8410965.

Asymmetry-based hierarchy was defined based on the group-averaged effective (directed) connectivity of cortical regions based on resting-state fMRI. The effective connectivity matrix (Fig 3A) shows the influence of each brain region on the activity of other regions during resting state, and was previously estimated using regression dynamical causal modelling (rDCM), based on the data from 40 healthy adults [48–51]. Using the effective connectivity matrix, we calculated the asymmetry-based hierarchy of each region as the difference between its weighted out-degree (efferent strength) and in-degree (afferent strength). The asymmetry-based hierarchy map was significantly correlated with LTC G1 (r = −0.39, p_variogram_ < 0.001), indicating higher asymmetry-based hierarchy of infragranular-dominant regions (Fig 3B). Accordingly, the asymmetry-based hierarchy map was significantly correlated with the relative thickness of layers III and IV negatively, and layers V and VI positively (S13 Fig). Of note, decomposing the asymmetry-based hierarchy into its components, we observed a significant correlation of LTC G1 with the weighted in-degree (r = 0.60, p_variogram_ < 0.001) but not out-degree (r = −0.01, p_variogram_ = 0.868) (S14 Fig). The asymmetry-based hierarchy map of a replication sample from the Human Connectome Project (HCP) dataset (*N* = 100) [50,52] was similarly correlated with LTC G1 (r = −0.49, p_variogram_ < 0.001; S15 Fig). Note that for the above analyses, we recalculated LTC and LTC G1 in the Schaefer-400 parcellation, as the effective connectivity matrices obtained from the previous work by Paquola and colleagues [50] were available in this parcellation.

In addition, we obtained the laminar-based hierarchy map of the macaque cerebral cortex from a previous study [27]. Laminar-based hierarchy assumes higher hierarchical positions for regions projecting FB and receiving FF connections, as quantified in tract-tracing studies [28,29]. After aligning the LTC G1 map of the human cerebral cortex to the macaque’s cerebral cortex in the left hemisphere [53], we observed that it was significantly correlated with the map of macaque’s laminar-based hierarchy (r = −0.54, p_variogram_ < 0.001; Fig 3C). In addition, the laminar-based hierarchy showed significant positive correlations with the relative thickness of layers III and IV, and negative correlations with the relative thickness of layers V and VI (S13 Fig). These findings indicated association of laminar thickness variation to 2 alternative maps of cortical hierarchy based on the asymmetry of effective functional connectivity and the laminar pattern of structural connections.

### Laminar thickness covariance links to interregional connectivity

**Fig 4:**
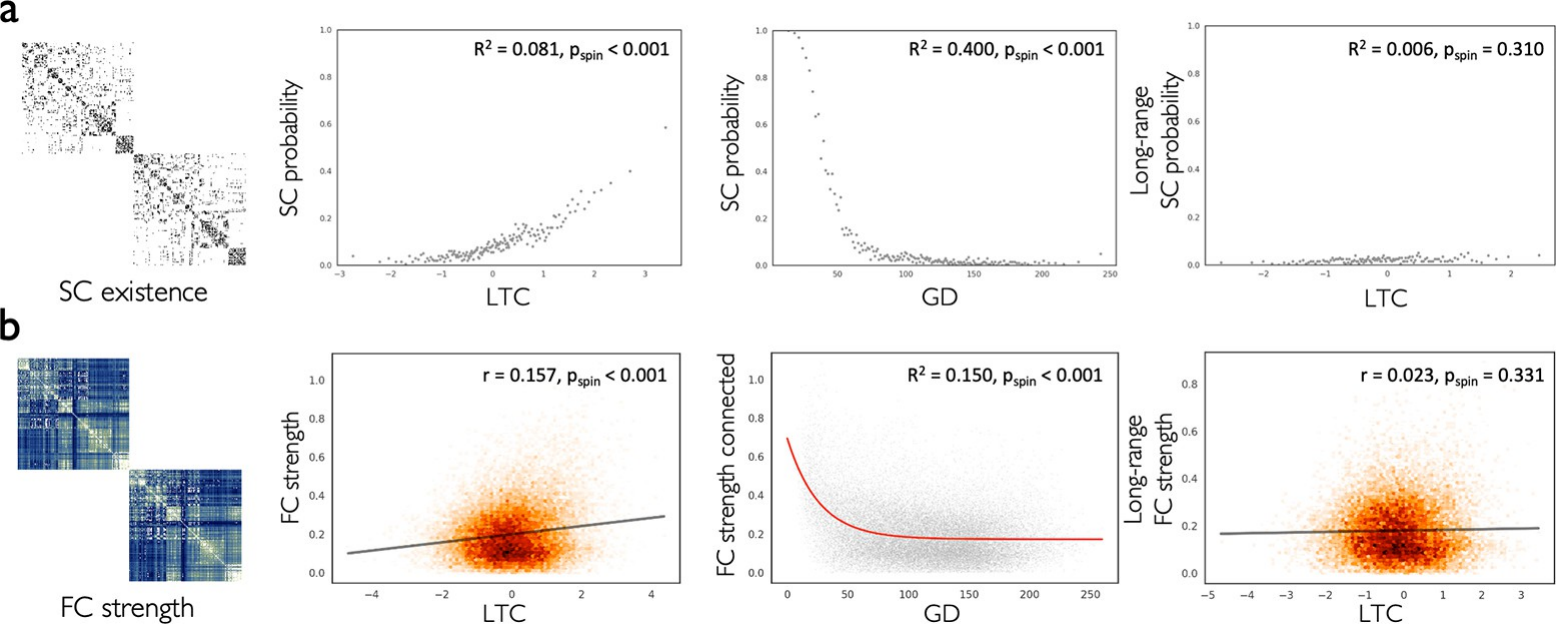
Association of laminar thickness covariance with connectivity. **(a)** The binarized SC matrix showing the existence of intrahemispheric connections (left). SC likelihood was associated with increased LTC (center left) and decreased GD (center right). SC likelihood among long-range connections was not significantly associated with LTC. **(b)** The FC matrix showing the strength of intrahemispheric connections (left). FC strength was correlated with increased LTC (center left) and decreased exponentially with GD (center right). FC strength among long-range connections was not significantly correlated with LTC. The data and code needed to generate this figure can be found in https://zenodo.org/record/8410965. FC, functional connectivity; GD, geodesic distance; LTC, laminar thickness covariance; SC, structural connectivity.

Having observed alignment of asymmetry- and laminar-based hierarchy with laminar thickness variation, we next studied whether the similarity of regions in laminar thickness relates to interregional connectivity in humans (Fig 4). We used the structural and functional connectivity (SC and FC) matrices (400 regions) averaged across a subgroup of the HCP dataset (*N* = 207) [52,54], which was obtained from the ENIGMA (Enhancing NeuroImaging Genetics through Meta-Analysis) Toolbox [55]. Using logistic regression, we observed higher LTC was associated with the increased likelihood of SC (R^2^ = 0.081, p_spin_ < 0.001). In addition, LTC was correlated with the increased strength of FC (r = 0.16, p_spin_ < 0.001). Neighboring regions in the cerebral cortex are more likely to connect [56,57] and also tend to have similar structural and functional features [58,59]. Here, we also observed this effect, with physically proximal regions showing higher likelihood of SC (R^2^ = 0.400, p_spin_ < 0.001) and strength of FC (R^2^ = 0.150, p_spin_ < 0.001) on one hand, and higher LTC (R^2^ = 0.164, p_spin_ = 0.004) on the other hand. To understand whether LTC was associated with connectivity independent of distance effects, we studied the association of LTC with long-range connectivity. We observed that LTC was not significantly associated with the likelihood of long-range SC (R^2^ = 0.006, p_spin_ = 0.310) or strength of long-range FC (r = 0.023, p_spin_ = 0.331). This finding suggested interregional distance as an important covariate in the association of LTC with connectivity.

### Laminar thickness covariance in association to covariance and maturational coupling of cortical thickness

Thus far, we described how the laminar structure varies across the isocortex and evaluated its relevance to cortical hierarchy and connectivity. Lastly, we sought to study potential links of individual-level LTC to population-level interregional covariance and maturational coupling of cortical thickness. Structural covariance matrix reflects the pattern of covariation in cortical morphology (e.g., cortical thickness) across a population, which provides a model of shared maturational and genetic effects between cortical regions [60–62]. We obtained the structural covariance matrix based on the HCP dataset (*N* = 1,113) from our previous work [62] and observed that it was significantly correlated with the LTC at the level of matrices (r = 0.33, p_spin_ < 0.001) and their principal axes (r = -0.55, p_variogram_ < 0.001). This may indicate shared maturational and genetic effects between regions with similar laminar thickness (S16A Fig). Next, we studied the association of LTC with the interregional maturational coupling matrix (MCM), obtained from a previous study by Khundrakpam and colleagues [61]. This matrix shows the similarity of regions in longitudinal cortical thickness changes over development in a dataset of children and adolescents (*N* = 140, baseline age = 11.9 ± 3.6, followed up for approximately 2 years) and was weakly correlated with the LTC matrix (r = 0.10, p_spin_ < 0.001) (S17 Fig).

## Discussion

In the current study, we sought to extend previous quantitative studies on cytoarchitectural variability of the cerebral cortex [1,10], by focusing on the layered structure of the cerebral cortex, and evaluated its links to cortical connectivity. We used the map of cortical layers [42] based on the ultrahigh-resolution atlas of BigBrain [9] to identify a principal axis of laminar thickness covariation in the isocortex. We observed an axis of LTC showing a shift from the dominance of supragranular towards infragranular layers thickness from the occipital to lateral frontal areas. This shift was coaligned with the cortical hierarchy, defined based on either the asymmetry of afferent and efferent connections in the human cerebral cortex or the laminar pattern of connections in the macaque cerebral cortex. We also found a higher likelihood of structural and strength of functional connections between regions with similar patterns of laminar thickness, supporting the principle of "similar prefers similar" in cortical wiring and the structural model of connectivity [3,17,22,26]. Finally, we showed that laminar thickness covariation was linked to the population-level interregional covariance of total-depth cortical thickness, suggesting potential shared maturational and genetic effects between regions with similar laminar thickness.

The principal axis of laminar thickness covariation characterized an overall increase in the relative thickness of supragranular layers from the lateral frontal to posterior occipital regions. This was in line with a previous animal study that illustrated relative increase in the implied column height of the upper layers along the rostro-caudal axis of the cerebral cortex in several rodent and nonhuman primate species [63]. The same study also reported that from rostral to caudal regions the density of neurons increases, as had been shown in a few other studies [64–66], but additionally reported the increase to be more prominent in layers II to IV rather than layers V to VI (without differentiating the individual layers in each layer group). In an integrated model of combined laminar thickness and intensity variations, we observed a rostral to caudal principal axis, similar to LTC G1, characterizing increased grey-matter density in all the layers, most prominently in layer IV. Using a preliminary dataset of laminar neuronal features in a few cortical regions of the BigBrain and based on automated labeling of 1-μm resolution images [67], we also observed increased neuronal density and decreased soma size along the integrated axis of laminar thickness and intensity, with the most prominent association found in layer IV. In addition, we observed an increased ratio of layer III to layer V average neuronal size, which may indicate externopyramidization along this axis. Of note, our cellular-level results should be interpreted with caution as they were limited to a small number of available samples located primarily at the two ends of LTC G1. Overall, from the lateral frontal towards parietal and occipital regions, there is an increase in the prominence of the granular and supragranular cortical layers relative to the infragranular layers, with respect to thickness, and potentially neuronal density and soma size.

Previous theory-based approaches based on visual inspection of histological samples have additionally described a sensory-fugal axis of laminar structure variation transitioning from sensory to paralimbic regions [4,14]. This sensory-fugal axis, which was mapped qualitatively, is overall different from the quantitative axis of laminar thickness covariation that we described. This divergence may be attributed to the different approaches and the laminar features studied. Here, we benefited from using a data-driven approach on more extensive and denser histological data, but in doing so, we focused on the gross laminar features including thickness and the average grey-matter density. On the other hand, theory-driven maps of laminar structure such as cortical types are determined based on a variety of different laminar features [14], yet some of the finer features such as the properties of individual neurons were invisible to our model. This highlights the importance of future work on higher-resolution images of BigBrain, enabling a data-driven model of laminar structure that incorporates both gross and fine laminar features. Nevertheless, we observed that regions belonging to the same cortical type may have variable laminar thickness patterns. This may indicate differential processes underlying different features of laminar structure and, more broadly, cytoarchitecture. In fact, a previous data-driven model of MPC in the BigBrain revealed 2 main axes of cytoarchitectural variability: a rostro-caudal and a sensory-fugal axis [10]. Beyond cytoarchitecture, additional features such as myeloarchitecture and receptor architecture vary across regions and such changes may be distinct from cytoarchitectural variation of laminar structure [68]. A recent study on the large-scale variation of layer-wise receptor densities in the human cerebral cortex based on autoradiography [69] reported a "natural axis" of receptor distribution [70]. This axis spanned from association areas with higher infragranular AMPA density towards sensory areas with pronounced supragranular NMDA density as well as a higher diversity of receptor densities, which was more prominent in infragranular layers. The different axes that we highlighted here may reflect diverging neurobiological routes organizing the human cerebral cortex. Indeed, in our previous work on group-level cortical thickness covariance and genetic correlations, we observed a rostral-caudal axis, which was suggested to reflect differentiation between cortical hierarchy and maturational effect, and a ventral-dorsal axis reflecting microstructural pattern associated with the theory of dual origin [62,71].

Over the past century, there has been a debate over the optimal approach and level of granularity to study cytoarchitectural variability of the cerebral cortex [1,72]. Previous studies have ranged from focusing on fine cytoarchitectural details and identification of sharp borders between regions [2,4] to classification of the cerebral cortex into broader categories with grossly comparable cytoarchitecture [4,14]. On the other hand, some authors have argued against cortex-wide existence of sharp boundaries and rather focused on the gradual variations across the cerebral cortex [5,72]. We should note that, here, we refrained from making any assumptions on the (non)existence of sharp borders or a level of granularity as we aimed to provide a whole-cortex layer covariance organizational axis. We argue that the topology of cytoarchitectural variability of the cerebral cortex ranges from abrupt to more gradual changes [1,72]. Accordingly, the LTC G1 map consisted of a combination of sharp borders and gradual transitions but was more dominated by gradual changes. We observed the LTC G1 map was consistent regardless of whether laminar thickness data were averaged into parcels or were analyzed at the level of vertices. This highlights that LTC G1 captures broader variations of laminar thickness across regions, in contrast to the finer local and intraregional variations. Focusing on the local variations, we observed a varying level of intraregional heterogeneity of laminar thickness across regions, as quantified by the average within- versus between-regional LTC. Specifically, primary visual area and orbital parts of inferior frontal gyrus were most homogeneous structures, whereas regions in temporal and parietal lobes showed high heterogeneity of laminar thickness. Indeed, recent work is increasingly showing patterns of intraregional cortical heterogeneity such as stripes of differential myelination in V2 [73], inter-effector areas in M1 [74] or differential gene expression in V1 associated with cortical layout of eccentricity [75]. Future work may uncover the spatial pattern and nature of such intraregional heterogeneities in laminar structure and use data-driven approaches to study the organization of borders and abrupt alterations of layer thickness and associated cytoarchitecture variation.

We observed that cortical hierarchy, defined using laminar pattern of connections in macaques and asymmetry of effective connections in humans, was aligned with the main axis of LTC. This finding extends previous observations on the link between laminar-based cortical hierarchy and microstructure [18,27]. The laminar pattern of corticocortical connections is suggested to relate to the gradation of cortical microstructure (the "structural model") [3,17,25,26] or the physical proximity of regions (the "distance rule model") [28,29]. These models suggest that the laminar connections of cytoarchitecturally similar or proximal regions are mostly lateral, but the pattern of connections become increasingly FF/FB as regions are more dissimilar in cytoarchitecture or are more distant [3,17,25,28,29,76]. Here, we observed that LTC G1 was aligned with the laminar-based hierarchy map in macaques, and asymmetry-based hierarchy map of humans. Specifically, regions towards the infragranular-dominant end of the axis were positioned higher in the cortical hierarchy than the supragranular-dominant regions. This observation potentially relates to the laminar patterns of FF and FB connections along the laminar-based hierarchy, as observed in tract-tracing studies [27–29,77]. FF connections originate from the supragranular layers II and III and target layer IV of a higher-order region, whereas FB connections originate from infragranular layers V and VI and terminate outside layer IV of a lower-order region [28,29,36,38,41], which may reciprocate FF connections [78]. In addition, lateral connections originate from supra- and infragranular layers and terminate across all the layers, connecting regions at a similar level [38]. Of note, more detailed accounts of neuronal projections have revealed additional patterns of FF and FB connections [28,29,37,39,79], such as a FB projections originating from layer II and FF projections originating from layers V and VI [28,29], or FF and lateral projections targeting layer I [37]. The FF and FB projections are thought to have distinct physiological roles, that is, FF projections carry high-dimensional (sensory) information up the hierarchy, whereas FB projections propagate context and modulate the function of lower-order regions [29,30,80]. Interestingly, the FF and FB connections are, respectively, associated with gamma and alpha/beta rhythms [29,30,40,81–83], which, in turn, show regional and laminar specificity, with more prominent gamma rhythms in early visual areas and superficial layers and beta rhythms in fronto-parietal areas and infragranular layers [29]. In fact, the asymmetry of FF and FB projections inferred based on magnetoencephalography has been previously used to map the cortical hierarchy of visual areas in humans [40]. In our comparison of LTC G1 with the laminar-based hierarchy map, we performed a cross-species comparison, yet we should note the limitations of this approach given the differences of humans and nonhuman primates in cortical cytoarchitecture [84] and connectivity [53,85]. There is some evidence based on cortical oscillations (c.f. above) and the pattern of intralaminar connectivity estimated using layer-based functional magnetic resonance imaging [86], which indicate increased FB dominance towards rostral regions in humans as well. Moreover, the human map of cortical hierarchy that we defined based on the asymmetry of effective connections showed a similar association with LTC G1 as the macaque’s laminar-based hierarchy. However, the definitions of asymmetry-based and laminar-based hierarchy are different [31] and may result in different maps, as was previously shown in the frontal cortex of macaques [35]. Layer-wise functional imaging is a promising approach that can be used to further investigate the association of laminar structure with the pattern of laminar connections and their functional implications in humans [86]. For example, recent work using layer-based functional magnetic resonance imaging could show that specific cortical layers are involved in different aspects of memory processing in the dorsolateral prefrontal cortex [87]. Such differences in cognitive processing may be rooted in the connectivity profiles associated with different layer depths that are embedded in the laminar structure.

We found that the similarity of regions in their laminar thickness patterns was associated with an increased likelihood of structural and strength of functional connections. This finding supports a principle of the structural model for connectivity that relates cytoarchitectural similarity to connectivity [3,17,20]. Our finding was in line with studies showing higher likelihood or strength of connections between regions with similar microstructure, based on the complexity of pyramidal neurons [23], neuronal density [22,24], or cortical types [19–22]. In addition, and of particular relevance to our findings, interareal connectivity in the human cerebral cortex has been linked to the MPC of the BigBrain [88–90]. Specifically, connected regions were reported to have higher similarity in their microstructural profiles compared to nonconnected profiles, and MPC correlated with the connectivity strength [90]. Furthermore, a previous study used generative modeling of connectivity and showed that including both microstructural profiles covariance and wiring cost in the model, as opposed to including wiring cost alone, leads to a better fit [88]. In addition, a low-dimensional coordinate space of the human cerebral cortex calculated by incorporating interregional SC, physical proximity, and the BigBrain’s microstructural covariance was shown to predict FC with a high accuracy [89]. In our study, we extend these findings and show that the probability and strength of connectivity additionally relates to the laminar thickness profiles in the BigBrain. Using an alternative approach, another recent study focused on the interrelation between connectivity and the absolute thickness of individual layers in the BigBrain and showed that regions with thicker layer IV are less likely to connect to regions with higher thickness in layers III, V, and VI [91]. Overall, these findings are in line with the wiring principle of "similar prefers similar" [21,22,88,91], which has been observed not only with the similarity of microstructure but also in association to gene expression patterns [92–96], neurotransmitter receptor profiles [97], and macroscale morphometry [91,98]. An alternative model of connectivity is the "distance rule model," which proposes physical proximity as the main predictor of connectivity as a result of wiring cost minimization [56,57,99–101]. It can be argued that the increased connectivity of similar regions may be an epiphenomenon of the distance rule, as nearby cortical regions tend to be similar [58,59]. However, it has been shown that the distance rule alone does not fully account for the connectome architecture. For example, simulated connectomes were shown to better resemble the empirical connectomes when interregional similarity was considered in addition to the wiring cost reduction [88]. Recent studies on tract-tracing data have shown that both similarity of cortical types and physical proximity can predict likelihood of structural connections [25,76], though in most species, cytoarchitectonic similarity was related to connectivity, above and beyond physical proximity [76]. Nevertheless, in our study, long-range connections were not significantly associated with similarity of laminar thickness profiles, suggesting distance as an important covariate. A decreased association of microstructural similarity and connectivity among long-range connections has been also observed in previous studies [90]. This raises the question of how long distance connections are encoded in layer-based architecture of the human cerebral cortex. Possibly, the uncoupling of layer similarity and long-distance connections could be in part driven by an uncoupling through activity-dependent organization, linked to the tethering hypothesis [102]. Further work integrating connectivity with layer-based approaches may help to further understand the interrelationship between short- and long-distance connections and cortical architecture.

Having studied the *what* and *why* questions of LTC, we also explored the question of *how* the (adult) laminar structure variations may come about. A central hypothesis on the origins of laminar structure variability proposes that different developmental trajectories across regions may relate to the gradation of laminar structure [17,22,103]. There are regional differences in neurogenesis timing and cell cycle duration throughout fetal development [104–110], or region- and layer-specific neuronal death in early postnatal stages [111], which may result in the specification of regions and their cytoarchitectural variability. For example, outer subventricular zone, a germinal zone of the developing cortex that is thought to generate the expanded primate granular and supragranular layers, is denser and deeper in area 17 compared to area 18 and has an increased rate of cell cycles, leading to a marked expansion of the upper layers in this region [104,105,109,112]. In the current study, we observed that higher interregional LTC was linked to higher population-level interregional structural covariance, which potentially indicates shared genetic and maturational effects among regions [60,62]. In addition, we observed a significant but weak correlation of LTC with subject-level longitudinal maturational coupling of cortical regions during childhood and adolescence [61] and found distinct pre- and postnatal developmental trajectories of genes overexpressed at the two ends of LTC G1 (S1 Text). Importantly, our current findings only indirectly suggest developmental relevance of laminar thickness organization. For example, the transcriptomics analysis involves mere spatial colocalization of the LTC G1 with the gene expression maps and the developmental enrichment of those genes and, therefore, lacks mechanistic insights on the complex gene regulatory mechanisms underlying regional differences of laminar structure. We refer the interested reader to the rich literature on cortical arealization and its genetic regulation [113–116]. Consequently, further research will be needed to study the developmental relevance of laminar structure variability by investigating *postmortem* histology or *in vivo* markers of laminar structure [10,117,118] at different stages of development to shed light on the maturation of laminar structure and its regional variability.

### Limitations and future directions

In this study, we used the whole-brain map of cortical layers from a single individual, the BigBrain [9,42]. This is currently the only whole-brain and high-resolution map of cortical layers available, and until a similar atlas becomes available, it is unclear how much our findings would generalize to the other individuals. Of note, when we compared left and right hemispheres of the same individual, we observed similar principal axes, which hints at intraindividual interhemispheric consistency of the principal axis of LTC. In addition to generalizability, an intriguing question for the future research is the degree to which laminar structure varies across individuals, and how it might relate to behavior and function, and its changes through development. This highlights the importance of future studies on *in vivo* estimation of laminar structure based on high-resolution imaging.

We studied LTC using a 6-layer model of the isocortex, previously created using a convolutional neural network [42]. However, it is well known that some isocortical areas have fewer or a greater number of layers, due to the individual layers being absent or being divided into sublayers [1,4,119]. For example, area V1 is characterized by a prominent layer IV that is divided into 3 sublayers, and on the other hand, layer IV is unclear in agranular regions [4,14,119]. To avoid forcing a 6-layer model in regions with fewer number of layers and less clear layer boundaries, we excluded a- and dysgranular regions from our analyses. Exclusion of these regions limits the generalizability of our findings to the whole extent of the isocortex, yet we showed that the LTC G1 map was consistent when these regions are included. In addition, to further explore the impact of a priori defined number of layers, we used a 3-layer model of supragranular, granular, and infragranular layers and observed a similar principal axis. This indicates that LTC G1 captures variations of thickness in the supragranular, granular, and infragranular layer groups rather than the individual layers within each group. Future research may account for the regional differences in the number of layers using more fine-grained models of intracortical structure where the number of layers in each location is determined based on the data rather than being fixed. This would enable formally testing the optimal architecture of cortical depth and enables inclusion of a-/dysgranular areas in a more comprehensive model of laminar structure in the cerebral cortex.

### Conclusions

In sum, we described an axis of laminar thickness covariation in the BigBrain, which characterized a structural shift from supra- to infragranular layer thickness. This shift was coaligned with the asymmetry- and laminar-based maps of cortical hierarchy, with infragranular-dominant regions positioned higher across the hierarchy. In addition, regional variation of laminar thickness in the isocortex was related to interregional connectivity, although not among the long-range connections. Future work may help further understand the relevance of laminar structural variation to human brain function across the lifespan, ultimately providing insights into how the anatomy of the human brain supports human cognition.

## Materials and methods

The current research complies with all relevant ethical regulations as set by The Independent Research Ethics Committee at the Medical Faculty of the Heinrich Heine University Duesseldorf (study number 2018–317). We used previously published data from various sources that have received ethics approval from their respective institutions.

### BigBrain maps of laminar thickness

BigBrain is a 3D histological atlas of a *postmortem* human brain (male, 65 years), which is created by digital reconstruction of ultrahigh-resolution sections (20 μm) stained for cell bodies, and is publicly available at https://ftp.bigbrainproject.org/ [9]. The cerebral cortex of the BigBrain was previously segmented into 6 layers, using a convolutional neural network trained on the samples manually segmented by expert anatomists [42]. We used the BigBrain laminar thickness data in the *bigbrain* surface space, which was included in the BigBrainWarp toolbox (https://bigbrainwarp.readthedocs.io) [120]. The BigBrain surface mesh and laminar thickness maps were downsampled from approximately 164 k to approximately 10 k points (vertices) per hemisphere to reduce the computational cost of the analyses. The surface mesh was downsampled by selecting a reduced number of vertices and retriangulating the surface while preserving the cortical morphology, and the surface data (e.g., laminar thickness) were downsampled by assigning the value of each maintained vertex to the average value of that vertex and its nearest removed vertices [120].

### BigBrain layer-specific distribution of neurons

Layer-specific neuronal density and size in selected tissue sections of the BigBrain isocortex at the resolution of 1 μm was obtained from the Python package *siibra* (https://siibra-python.readthedocs.io/en/latest). This dataset was created by manual annotation of cortical layers and automatic segmentation of neuronal cell bodies (https://github.com/FZJ-INM1-BDA/celldetection) [67]. It contains the data for 111 tissue sections, corresponding to 80 vertices on the BigBrain downsampled surface.

### Laminar thickness covariance

We first excluded regions of the brain with the agranular or dysgranular cortical type due to the less clear definition of the layer boundaries in these regions [14]. The map of cortical types was created by assigning each von Economo region [121] to one of the 6 cortical types, including agranular, dysgranular, eulaminate I, eulaminate II, eulaminate III, and koniocortex, based on manual annotations published by García-Cabezas and colleagues [14]. Next, for each individual layer and in each hemisphere, the thickness maps were smoothed using a moving disk with a radius of 10 mm to reduce the local effects of curvature on laminar thickness. Specifically, the cortical surface mesh was inflated using FreeSurfer 7.1 (https://surfer.nmr.mgh.harvard.edu/) [122], and for each vertex, a disk was created by identifying its neighbor vertices within a Euclidean distance of 10 mm on the inflated surface, and a uniform average of the disk was calculated as the smoothed laminar thickness at that vertex. Next, to obtain the relative laminar structure at each vertex, laminar thicknesses were divided by the total cortical thickness. Finally, the maps of laminar thickness were parcellated using the Schaefer 1000-region atlas [123], of which 889 regions were outside a-/dysgranular cortex and were included in the analyses. The parcellation was performed by taking the median value of the vertices within each parcel. Alternative parcellation schemes, including the Schaefer 400-region [123], Desikan-Killiany (68 regions) [124], AAL (78 cortical regions) [125], AAL subdivided into 1,012 regions [126], and the HCP Multi-Modal Parcellation (360 regions) [127], were used to show robustness of findings and to enable associations of LTC with the data available in specific parcellations. In addition, we used a homotopic local-global parcellation by Yan and colleagues for the comparison of left and right hemispheres [128]. Of note, the parcellation maps that were originally in the *fsaverage* space were transformed to the *bigbrain* space, based on multimodal surface matching and using BigBrainWarp [120,129]. One parcellation (AAL) was originally available in the *civet* space and was transformed to *fsaverage* using neuromaps [130] before being transformed to the *bigbrain* space. In addition to the different parcellation schemes, to further evaluate robustness of our findings to the effect of parcellation, the analyses were also repeated on unparcellated data at the level of vertices.

LTC between the cortical regions was calculated by performing pairwise partial correlation of relative laminar thicknesses, while controlling for the average laminar thickness across the isocortex, to identify greater-than-average covariance. The partial correlation coefficients were subsequently Z-transformed, resulting in the LTC matrix. We also used alternative covariance metrics including full Pearson correlation as well as Euclidean distance in the robustness analyses.

### Geodesic distance

The geodesic distance between 2 points on the cortical surface refers to the length of the shortest path between them on the mesh-based representation of the cerebral cortex. Using the Connectome Workbench (https://www.humanconnectome.org/software/connectome-workbench) [131], we calculated the geodesic distance between the centroids of each pair of parcels, where the centroid was defined as the vertex that has the lowest sum of Euclidean distance from all other vertices within the parcel. The geodesic distance calculation was adapted from its implementation in micapipe (https://micapipe.readthedocs.io) [132]. To evaluate whether our findings were robust to the effect of geodesic distance, in some analyses, the effect of geodesic distance on LTC was regressed out using an exponential regression.

### Cortical folding

Mean curvature was calculated at each vertex of the midcortical surface based on the Laplace–Beltrami operator using *pycortex* (https://gallantlab.github.io/pycortex/) [133]. To compute the curvature similarity matrix, for each pair of parcels, we estimated their similarity in the distribution of mean curvature across their vertices. This was achieved by calculating Jensen–Shannon divergence of their respective probability density functions.

### Microstructural profile covariance and laminar intensity covariance

The image intensity of the cell body–stained BigBrain atlas reflects neuronal density and some size, and its variation across cortical depth at each cortical location is referred to as "cortical profile" or "microstructural profile." We obtained the microstructural profiles sampled at 50 equivolumetric surfaces along the cortical depth from the BigBrainWarp toolbox [120] and reproduced the histological MPC matrix as previously done by Paquola and colleagues [10,120]. The microstructural profiles were first parcellated by taking the median. Subsequently, MPC matrix was calculated by performing pairwise partial correlations of regional microstructural profiles, controlled for the average microstructural profile across the isocortex.

In addition, we created layer-specific cortical profiles by sampling the BigBrain image intensity at 10 equivolumetric surfaces along the depth of each layer, which were then averaged, creating 6 laminar intensity maps. Subsequently, an LIC matrix was created similar to the approach described above for the LTC and MPC matrices.

### Effective connectivity

We obtained the effective connectivity matrix from a prior study [50] based on the microstructure informed connectomics (MICs) cohort (*N* = 40; 14 females, age = 30.4 ± 6.7) [51] as well as a replication sample from the minimally preprocessed S900 release of the HCP dataset (*N* = 100; 66 females, age = 28.8 ± 3.8) [52,54]. The effective connectivity matrix between Schaefer 400 parcels was estimated based on rs-fMRI scans using regression dynamic causal modelling [48,49], freely available as part of the TAPAS software package [134]. This approach is a computationally highly efficient method of estimating effective, directed connectivity strengths between brain regions using a generative model.

The effective connectivity matrix was used to estimate asymmetry-based hierarchy, which assumes that hierarchically higher regions tend to drive the activity in other regions rather than their activity being influenced by them. Therefore, given the effective connectivity matrix, after converting it to an unsigned matrix, we calculated the regional asymmetry-based hierarchy as the difference of the weighted out-degree and in-degree of each region, assuming higher hierarchical position for regions with higher efferent than afferent strength.

### Macaque map of cortical hierarchy

The macaque map of laminar-based hierarchy was obtained from a previous work by Burt and colleagues [27]. Briefly, this map was created by applying a generalized linear model to the laminar projection data, based on the publicly available retrograde tract-tracing data (http://core-nets.org) [28,77], resulting in hierarchy values in 89 cortical regions of macaque’s M132 parcellation [135–137]. To compare the macaque cortical map of laminar-based hierarchy to the human maps of LTC G1 and thickness of individual layers, we aligned these maps to the macaque cerebral cortex using the approach developed by Xu and colleagues [53]. Specifically, we first transformed the unparcellated human maps from the *bigbrain* space to the human *fs_LR* space using BigBrainWarp [120], mapped it to macaque *fs_LR* space using the Connectome Workbench, and, finally, parcellated the transformed map in macaque *fs_LR* space using M132 parcellation.

### Structural and functional connectivity

The group-averaged FC and SC matrices based on a selected group of unrelated healthy adults (*N* = 207; 124 females, age = 28.7 ± 3.7) from the HCP dataset [52,54] were obtained from the publicly available ENIGMA Toolbox (https://github.com/MICA-MNI/ENIGMA) [55]. We fetched the connectivity matrices created in the Schaefer 400 parcellation. We refer the reader to the ENIGMA Toolbox publication and online documentations for the details on image acquisition and processing. Briefly, the FC matrix for each subject was generated by computing pairwise correlations between the time series of all cortical regions in a resting-state fMRI scan, which, after setting negative correlations to zero and Z-transformation, were aggregated across the participants. The SC matrices were generated from preprocessed diffusion MRI data using tractography performed with MRtrix3 [138] and were group-averaged using a distance-dependent thresholding procedure.

### Structural covariance, genetic correlation, and environmental correlation

We obtained the structural covariance matrix, as well as the interregional genetic and environmental correlation matrices from our previous work [62]. The structural covariance was based on the cortical thickness values of the Schaefer-400 parcels in each individual and was computed by correlating the cortical thickness values between regions and across HCP participants (*N* = 1,113; 606 females, age = 28.8 ± 3.7) [52,54], while controlling for age, sex, and global thickness. Twin-based bivariate polygenic analyses were then performed to decompose the phenotypic correlation between cortical thickness samples to genetic and environmental correlations.

### Maturational coupling

We obtained the group-averaged matrix of maturational coupling from previous work by Khundrakpam and colleagues based on a sample of children and adolescents (*N* = 141; 57 females, age at baseline = 11.9 ± 3.6) who were scanned 3 times during a 2-year follow-up [61]. In this study, subject-based maturational coupling was calculated between 78 cortical regions of the AAL parcellation as their similarity in the slope of longitudinal changes in cortical thickness across 3 time points. Subject-based maturational coupling matrices were subsequently pooled into a group-averaged matrix.

### Dimensionality reduction of matrices

We applied the gradients approach implemented in the BrainSpace toolbox to identify the main axes (gradients) along which cortical regions can be ordered with regard to their similarity in the input matrix [47]. In this approach, to reduce the influence of noise on the embedding, the matrix is first sparsified according to the parameter *p* (default: 0.9), by zeroing out the *p* lowest-ranking cells in each row of the matrix. Next, the normalized angle similarity kernel function is used to compute the affinity matrix. Subsequently, principal component analysis, a linear dimensionality reduction technique, is applied to the affinity matrix to estimate the macroscale gradients. Of note, to evaluate the robustness of our findings to analytical choices, we repeated this approach with alternative values of sparsity, as well as other, nonlinear dimensionality reduction techniques including Laplacian eigenmaps and diffusion map embedding. As the signs of gradient values are arbitrary and for consistency in interpretation, the gradients in these alternative configurations were flipped if needed to match the sign of the original gradient values. The gradients approach was performed on the matrices of LTC, MPC, and structural covariance. In addition, the gradients approach was applied to the fused matrices of LTC and LIC. Following a previous work [89], the matrix fusion was performed by rank-normalizing both matrices, followed by rescaling LIC ranks to that of LTC, and then horizontally concatenating the matrices.

### K-means clustering

We additionally used K-means clustering on the relative laminar thickness data to create a discrete map of laminar structure variability, as an alternative to the continuous map created using the gradients approach. The optimal number of clusters was identified using the *yellowbrick* package [139], by iteratively increasing the number of clusters, measuring the distortion score for each number of clusters, and identifying the elbow, after which adding more clusters does not considerably improve the model performance. The K-means clustering was performed using the *scikit-learn* package [140].

### Intraregional heterogeneity

The vertex-level LTC matrix was calculated in the downsampled *bigbrain* surface by using vertex-level smoothed laminar thickness patterns. Next, for every region, defined using the Brodmann map, we calculated the average LTC of all vertex pairs within the same region, as well as pairs with other regions. We quantified intraregional heterogeneity of laminar thickness as the difference of average within- versus between-region LTC.

### Matrix associations

#### Matrix correlations

The Pearson correlation of any 2 given matrices was calculated after realigning the matrices to each other and removing the edges that were undefined in either matrix, across the edges in the lower triangle of the matrices. The null hypothesis testing for matrix correlations were performed nonparametrically, by creating a null distribution of correlation values calculated after random spinning of the parcels in one of the matrices for 1,000 permutations. The spinning of parcels was performed using the ENIGMA Toolbox [55,141,142].

#### Association to geodesic distance

The effect of geodesic distance on continuous matrices was evaluated using an exponential fit, and its statistical significance was assessed based on pseudo R^2^ and using spin tests, similar to the approach used for matrix correlations.

#### Association of LTC to the cortical types

The average LTC for pairs of parcels within the same cortical type was calculated and compared to the average LTC for pairs of parcels that belonged to different cortical types using permutation testing. In each permutation (*n* = 1,000), we spun the parcels in the LTC matrix, as described above, and calculated the difference of average LTC for the edges in the same versus different cortical types (for each cortical type, as well as across all cortical types), resulting in the null distribution of LTC differences within/between cortical types, which was used to calculate the *p*-values.

#### Association of LTC and geodesic distance to the connectivity probability

The SC matrix was binarized and logistic regressions were used to evaluate how connectivity probability relates to LTC and geodesic distance. The logistic regressions were performed using *statsmodels* package (https://www.statsmodels.org/stable/index.html) [143]. In each model, pseudo R^2^ was reported and its statistical significance was assessed nonparametrically, using 1,000 spin permutations of the LTC or geodesic distance matrix, as described above. The continuous changes in probability of connectivity as a function of LTC and geodesic distance were visualized by segmenting all the edges into 200 nonoverlapping windows, sorted by the value of predictor, and plotting the probability of connectivity within each window, which was calculated by dividing the number of connected edges by the total number of edges within the window.

### Surface associations

#### Correlation of continuous maps

Brain regions that are closer tend to be more similar in their features compared to spatially distant regions, due to the spatial autocorrelation [58,59]. In null-hypothesis testing of surface data correlations, it is important to take the spatial autocorrelation into account and evaluate the correlation coefficients against a null model in which the spatial autocorrelation is preserved [59]. Therefore, we assessed the statistical significance for the correlation of surface maps using BrainSMASH (Brain Surrogate Maps with Autocorrelated Spatial Heterogeneity) (https://brainsmash.readthedocs.io/en/latest/) [59,144]. In this approach, surrogate surface maps are simulated with spatial autocorrelation that is matched to spatial autocorrelation in the original surface map, through creating random maps whose variograms are approximately matched to that of the original map. Of note, a few number of the reported correlations were performed between unparcellated data and at the level of vertices, and for these cases, we used an alternative approach of creating surrogates that preserve spatial autocorrelation, namely, by randomly spinning the sphere representation of the cortical mesh using the BrainSpace toolbox [47,141]. Subsequently, for the statistical testing of the correlation between surface maps X and Y, we generated 1,000 surrogates of X and created a null distribution by calculating the correlation coefficient of each X surrogate with the original Y and compared the original correlation coefficient against this null distribution to calculate the *p*-value. Furthermore, for correlation of LTC G1 with cellular laminar features, given the sparsity of samples in the latter (*N* = 80 vertices), we used Spearman correlation.

#### Association of continuous and categorical surface maps

The association of categorical surface maps with the continuous maps was assessed using one-way ANOVA, combined with post hoc independent *t* tests, which were corrected for multiple comparisons using Bonferroni correction. These tests were performed using spin permutation, by spinning the parcels of the continuous map and creating null distributions based on 1,000 spun surrogates. The spinning of parcels was performed using the ENIGMA Toolbox [55,141,142]. To visualize continuous-categorical associations, we either plotted the proportion of each category within each bin of the continuous variable, or used raincloud plots [145].

## Supporting information

S1 Fig Effect of curvature on laminar thickness and laminar thickness covariance before and after smoothing. **(a)** The relative thickness of superficial layers decreases from sulci (negative curvature) to gyri (positive curvature) (left). After smoothing of the laminar thickness maps, the effect of curvature on laminar thickness was reduced remarkably, and the correlation of curvature with the relative thickness of superficial layers decreased (right). **(b)** The matrix shows the similarity of parcels in their distribution of curvature values based on Jensen–Shannon divergence (left). The correlation of curvature similarity matrix with the laminar thickness covariance (LTC) matrix decreased after smoothing (right). **(c)** The curvature map (left) was significantly correlated with the principal axis of LTC (LTC G1), but the effect decreased after smoothing (right). The data and code needed to generate this figure can be found in https://zenodo.org/record/8410965.
(TIF)

S2 Fig Laminar thickness covariance for regions of interest. The laminar thickness covariance maps (left hemisphere) are shown for the centroid vertex of selected regions including the left primary visual cortex (V1), primary auditory cortex (A1), primary somatosensory cortex (S1), Broca’s area, frontal pole, and primary motor cortex (M1). The data and code needed to generate this figure can be found in https://zenodo.org/record/8410965.
(TIF)

S3 Fig The first 3 axes of laminar thickness covariation. Left top: The first 3 gradients collectively explained 63.7% of the variance in laminar thickness covariance (LTC). Left bottom: The scatter plot shows the position of brain regions in the gradient space of G1, G2, and G3. Center: LTC G1, G2, and G3 projected on cortical surface show regional variation of laminar thickness across different axes. Right: The pattern of relative laminar thickness variation along the 3 main axes. The data and code needed to generate this figure can be found in https://zenodo.org/record/8410965.
(TIF)

S4 Fig Robustness of the principal axis of laminar thickness covariance to analytical choices. The principal axis of laminar thickness covariance (LTC G1) spatial map was robust to the analytical choices. **(a-d)** The maps of LTC G1 (left hemisphere) created using alternative analytical choices and their correlation with the original gradient are shown. **(e)** The correlation of gradients created using different degrees of sparsity applied to the laminar thickness covariance matrix, from 0 to 0.9. The data and code needed to generate this figure can be found in https://zenodo.org/record/8410965.
(TIF)

S5 Fig Hemisphere-specific axes of laminar thickness covariance. Laminar thickness covariance (LTC) and its principal axis was calculated separately on the left and right hemispheres. The principal axes of left and right hemispheres were significantly correlated. The data and code needed to generate this figure can be found in https://zenodo.org/record/8410965.
(TIF)

S6 Fig Association of laminar thickness covariance with geodesic distance. Geodesic distance (left) showed an inverse exponential relationship with the laminar thickness covariance, indicating similar laminar thickness patterns between neighbor regions (center). The principal axis of LTC (LTC G1) after regressing out the effects of geodesic distance (right) was significantly correlated with the original LTC G1 (r = 0.97, p_variogram_ < 0.001), indicating robustness of LTC G1 to geodesic distance. The data and code needed to generate this figure can be found in https://zenodo.org/record/8410965.
(TIF)

S7 Fig Principal axis of laminar thickness covariance using the 3-layer model. The principal axis of laminar thickness covariance (LTC G1) created using a 3-layer model including supragranular (I-III), granular (IV), and infragranular (V-VI) layers was correlated with the original 6-layer model LTC G1 and similarly described a shift from the dominance of infragranular to granular and supragranular layers. The data and code needed to generate this figure can be found in https://zenodo.org/record/8410965.
(TIF)

S8 Fig K-means clustering of cortical regions based on relative laminar thickness. **(a)** The distortion score of K-means clustering for the different number of clusters. The optimal number of clusters based on the elbow method was selected as 4. **(b)** Cluster of regions based on relative laminar thickness. **(c)** Laminar thickness profiles of brain regions in each cluster. **(d)** The principal axis of laminar thickness covariance (LTC G1) values were significantly different between the clusters (F = 813.1, p_spin_ < 0.001). Post hoc spin tests (Bonferroni corrected) showed significantly different LTC G1 values between all pairs of clusters except 2 and 3. The data and code needed to generate this figure can be found in https://zenodo.org/record/8410965.
(TIF)

S9 Fig Intraregional homogeneity of laminar thickness covariance. Laminar thickness covariance (LTC) homogeneity was calculated as the difference of average LTC between vertices that belong to the same region (LTC_intra_) versus other regions (LTC_inter_). Here, we used Brodmann areas as the map of cortical regions. The data and code needed to generate this figure can be found in https://zenodo.org/record/8410965.
(TIF)

S10 Fig Microstructural profile covariance in association with laminar thickness covariance. **(a)** The average regional microstructural profiles show variations of BigBrain image intensity across cortical depth (50 samples). Microstructural profile covariance (MPC) matrix was created by the pairwise partial correlation of intensity profiles between the parcels. **(b)** The principal axis of MPC created using principal component analysis. **(c)** The correlation between MPC and laminar thickness covariance (LTC) matrices. **(d)** The correlation between main axes of LTC and MPC. The data and code needed to generate this figure can be found in https://zenodo.org/record/8410965.
(TIF)

S11 Fig Laminar cellular features across the principal axis of laminar thickness covariance. **(a)** Locations of cortical samples for which laminar cellular data were available. **(b)** Neuronal segmentation across cortical layers in an example sample. **(c)** Variation of laminar neuronal density and size along the principal axis of laminar thickness covariation among the available samples. The data and code needed to generate this figure can be found in https://zenodo.org/record/8410965.
(TIF)

S12 Fig Association of cortical types with laminar thickness covariance. **(a)** The map of cortical types (left hemisphere) shows increasing laminar differentiation from agranular (green) to koniocortical (red) regions. **(b)** The average laminar thickness covariance (LTC) among pairs of parcels with the same or different cortical types, excluding agranular and dysgranular regions. Koniocortical regions showed significantly higher within-, compared to between-type average LTC. **(c)** Distribution of the principal axis of LTC (LTC G1) across the cortical types are shown in a raincloud plot. No significant difference in LTC G1 values was observed between the cortical types (F = 6.41, p_spin_ = 0.633). The data and code needed to generate this figure can be found in https://zenodo.org/record/8410965.
(TIF)

S13 Fig Association of asymmetry- and laminar-based hierarchy with the relative thickness of individual layers. Bar length shows the correlation coefficient and its color represents the level of statistical significance from white (p_variogram, FDR_ > 0.05) to black (p_variogram, FDR_ < 0.001). The data and code needed to generate this figure can be found in https://zenodo.org/record/8410965.
(TIF)

S14 Fig Afferent and efferent connectivity strength in association with the principal axis of laminar thickness covariance. The principal axis of laminar thickness covariance (LTC G1) was significantly correlated with regional weighted in-degree (afferent strength) (top) but not weighted out-degree (efferent strength) (bottom). The data and code needed to generate this figure can be found in https://zenodo.org/record/8410965.
(TIF)

S15 Fig Association of LTC G1 with asymmetry-based hierarchy in the replication dataset. **(a)** The group-averaged effective connectivity matrix of the replication sample (*N* = 100) based on regression dynamic causal modeling. **(b)** Regional asymmetry-based hierarchy was calculated as the difference between their weighted unsigned out-degree and in-degree and was significantly correlated with principal axis of laminar thickness covariance (LTC G1). The data and code needed to generate this figure can be found in https://zenodo.org/record/8410965.
(TIF)

S16 Fig Laminar thickness covariance in association to structural covariance. **(a)** The structural covariance matrix based on cortical thickness (left) in association with the laminar thickness covariance (LTC; center left). Main axes of structural covariance (center right) and LTC were correlated (right). **(b)** Interregional genetic and environmental correlation matrices based on cortical thickness in the HCP sample and their correlation with laminar thickness covariance. The data and code needed to generate this figure can be found in https://zenodo.org/record/8410965.
(TIF)

S17 Fig Laminar thickness covariance in association to maturation coupling. Maturational coupling matrix (MCM) was weakly associated with the laminar thickness covariance matrix (LTC). The data and code needed to generate this figure can be found in https://zenodo.org/record/8410965.
(TIF)

S1 Text Transcriptomics analyses of laminar thickness covariance. (PDF)

## Abbreviations

BrainSMASH: Brain Surrogate Maps with Autocorrelated Spatial Heterogeneity
ENIGMA: Enhancing NeuroImaging Genetics through Meta-Analysis
FB: feedback
FC: functional connectivity
FF: feedforward
HCP: Human Connectome Project
LIC: laminar intensity covariance
LTC: laminar thickness covariance
MCM: maturational coupling matrix
MIC: microstructure informed connectomics
MPC: microstructural profile covariance
rDCM: regression dynamical causal modelling
SC: structural connectivity
