# Supplementary figures and images for: The regional variation of laminar thickness in the human isocortex is related to cortical hierarchy and interregional connectivity
Source: PLoS Biol. 2023 Nov 9;21(11):e3002365. doi: 10.1371/journal.pbio.3002365 (PMC10684102; doi:10.1371/journal.pbio.3002365)

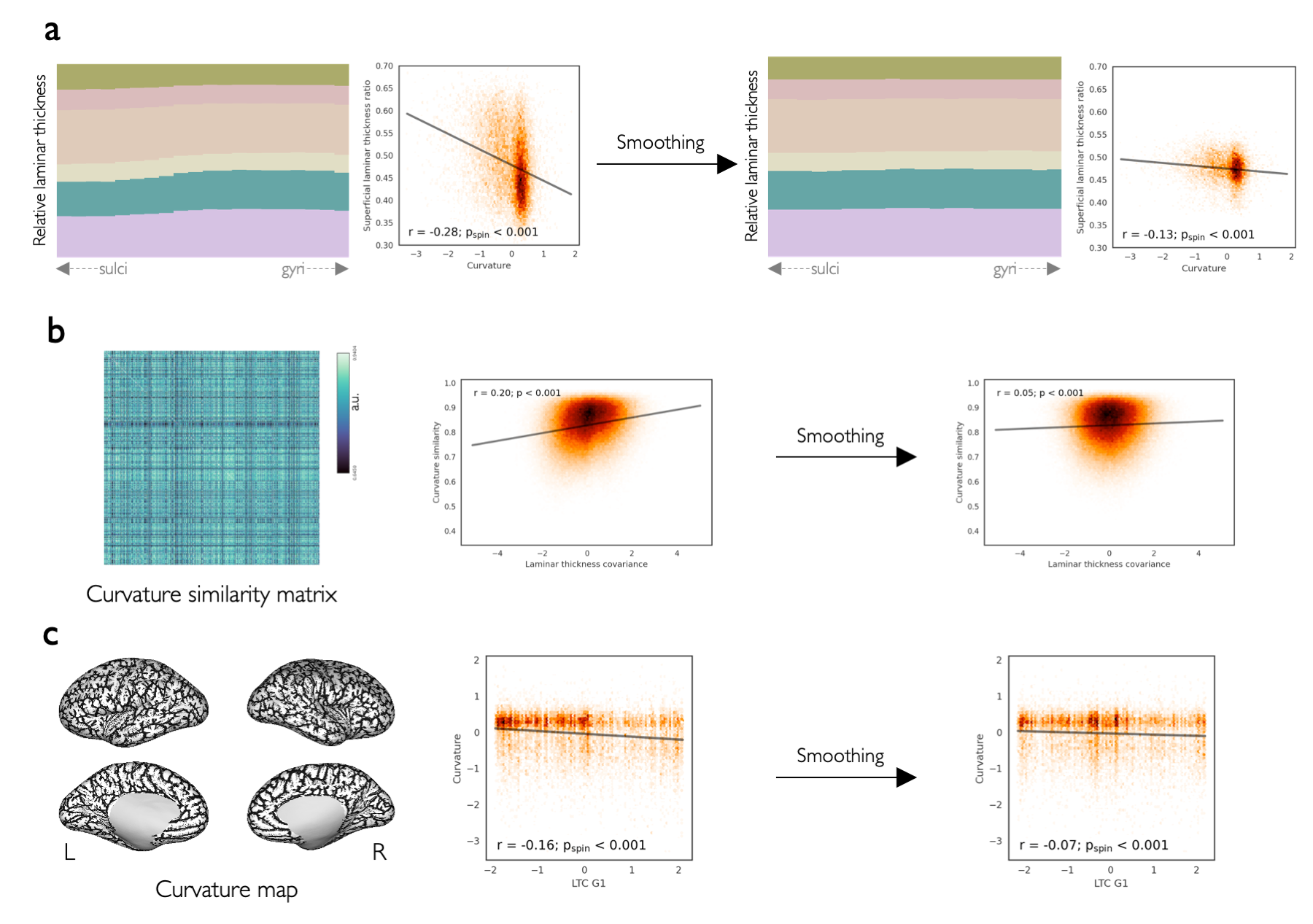

Supplement: S1 Fig — (a) The relative thickness of superficial layers decreases from sulci (negative curvature) to gyri (positive curvature) (left). After smoothing of the laminar thickness maps, the effect of curvature on laminar thickness was reduced remarkably, and the correlation of curvature with the relative thickness of superficial layers decreased (right). (b) The matrix shows the similarity of parcels in their distribution of curvature values based on Jensen–Shannon divergence (left). The correlation of curvature similarity matrix with the laminar thickness covariance (LTC) matrix decreased after smoothing (right). (c) The curvature map (left) was significantly correlated with the principal axis of LTC (LTC G1), but the effect decreased after smoothing (right). The data and code needed to generate this figure can be found in https://zenodo.org/record/8410965. (TIF) [file pbio.3002365.s001.tif]

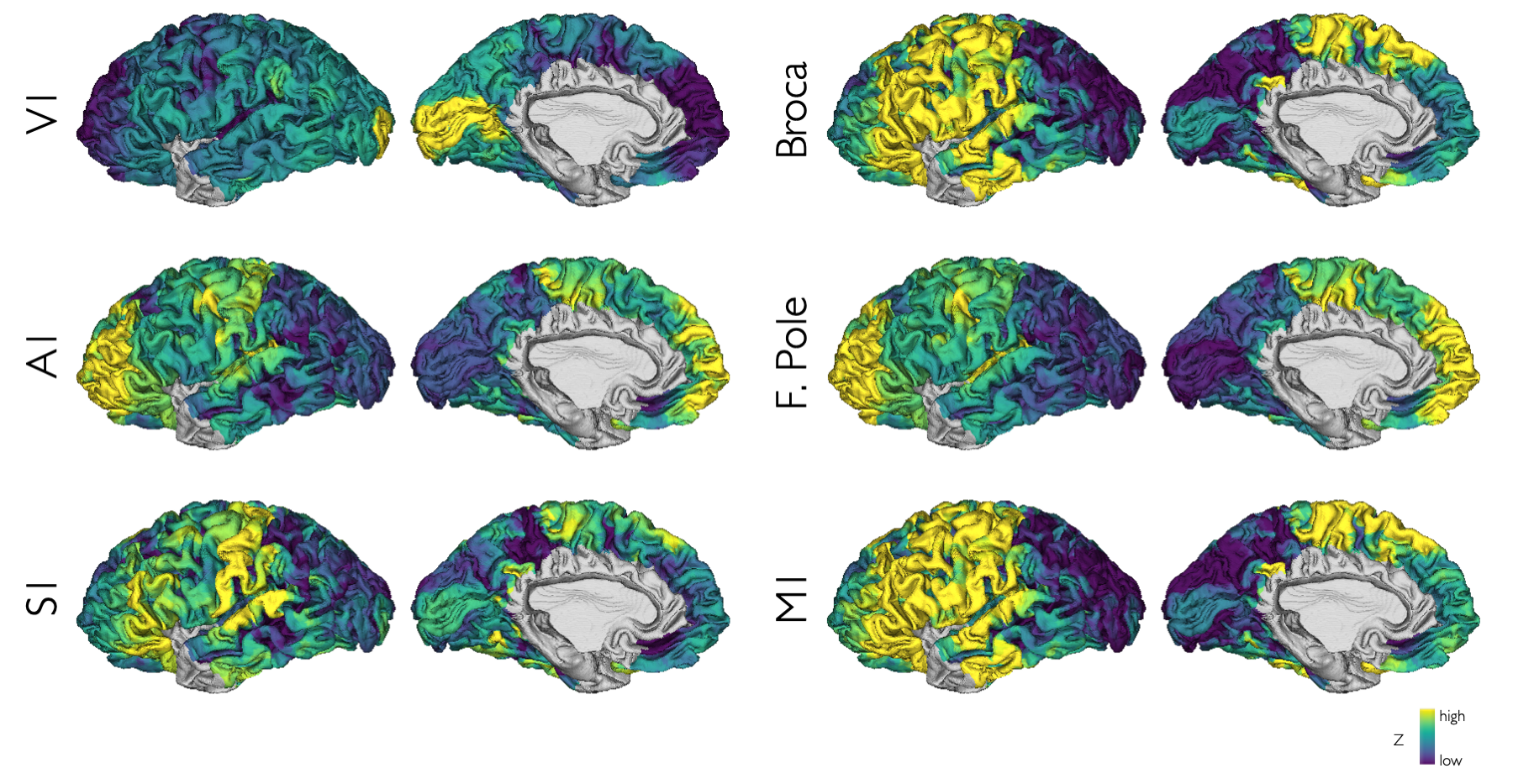

Supplement: S2 Fig — The laminar thickness covariance maps (left hemisphere) are shown for the centroid vertex of selected regions including the left primary visual cortex (V1), primary auditory cortex (A1), primary somatosensory cortex (S1), Broca’s area, frontal pole, and primary motor cortex (M1). The data and code needed to generate this figure can be found in https://zenodo.org/record/8410965. (TIF) [file pbio.3002365.s002.tif]

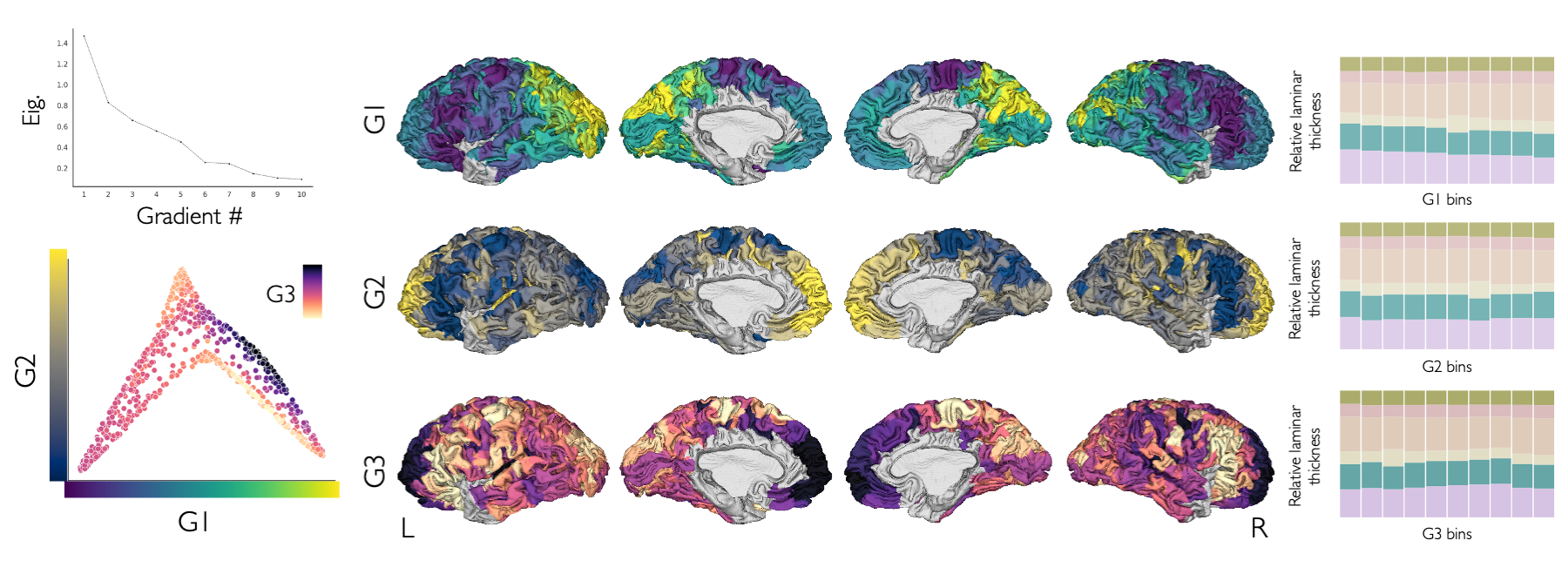

Supplement: S3 Fig — Left top: The first 3 gradients collectively explained 63.7% of the variance in laminar thickness covariance (LTC). Left bottom: The scatter plot shows the position of brain regions in the gradient space of G1, G2, and G3. Center: LTC G1, G2, and G3 projected on cortical surface show regional variation of laminar thickness across different axes. Right: The pattern of relative laminar thickness variation along the 3 main axes. The data and code needed to generate this figure can be found in https://zenodo.org/record/8410965. (TIF) [file pbio.3002365.s003.tif]

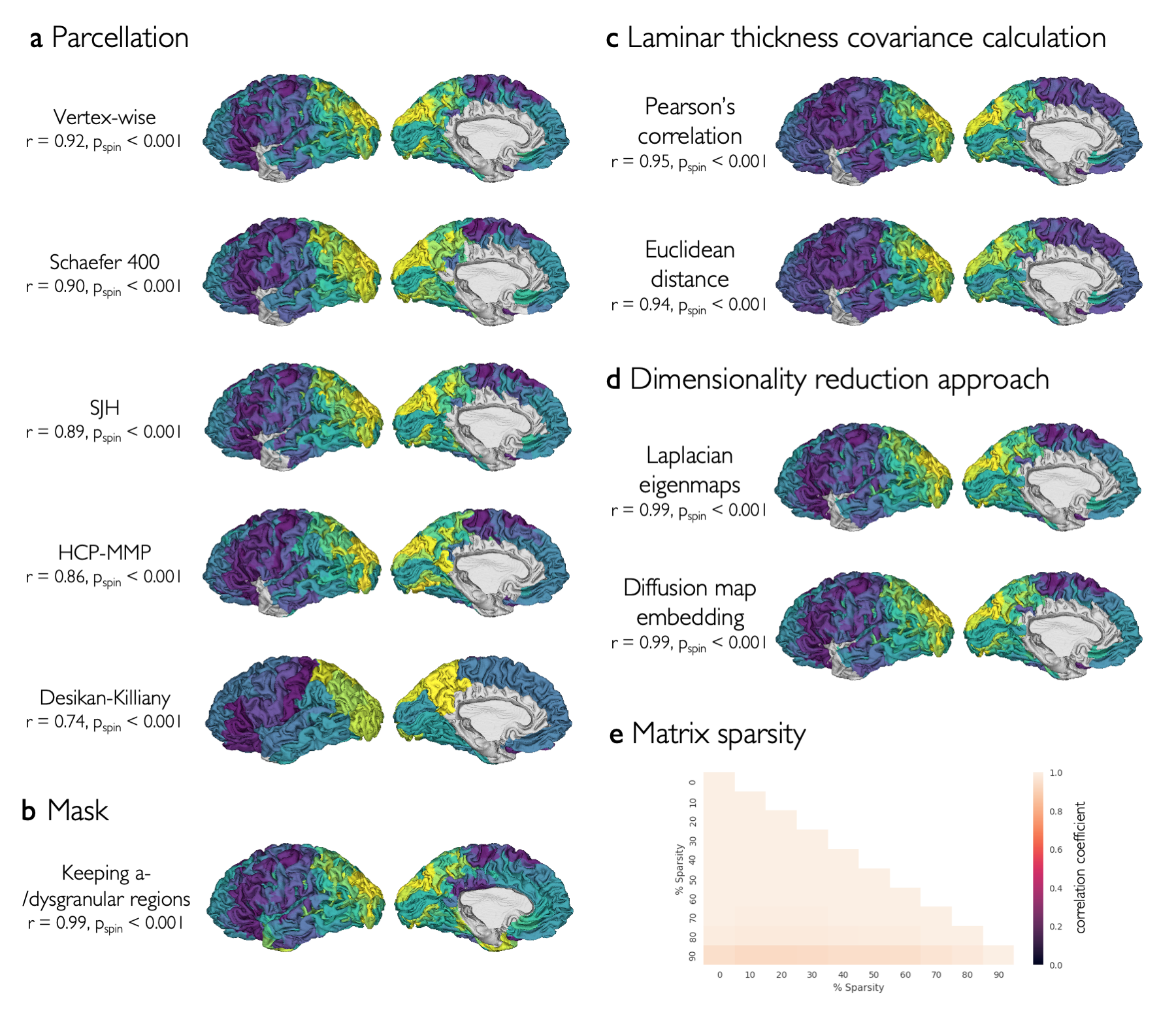

Supplement: S4 Fig — The principal axis of laminar thickness covariance (LTC G1) spatial map was robust to the analytical choices. (a-d) The maps of LTC G1 (left hemisphere) created using alternative analytical choices and their correlation with the original gradient are shown. (e) The correlation of gradients created using different degrees of sparsity applied to the laminar thickness covariance matrix, from 0 to 0.9. The data and code needed to generate this figure can be found in https://zenodo.org/record/8410965. (TIF) [file pbio.3002365.s004.tif]

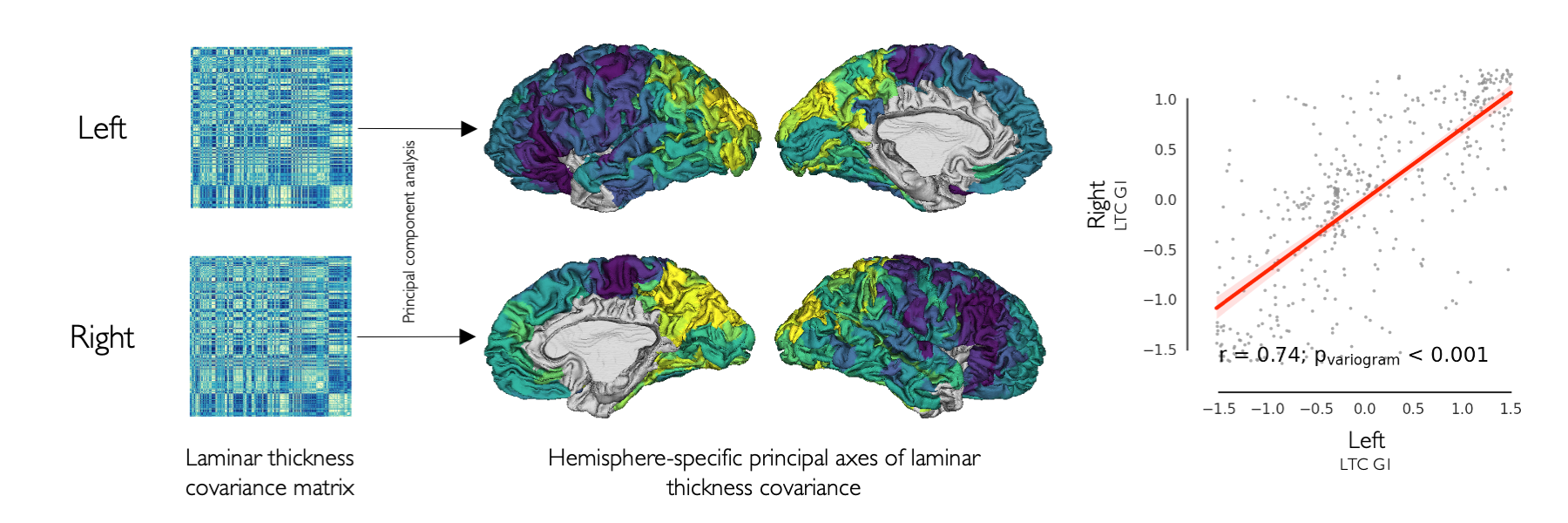

Supplement: S5 Fig — Laminar thickness covariance (LTC) and its principal axis was calculated separately on the left and right hemispheres. The principal axes of left and right hemispheres were significantly correlated. The data and code needed to generate this figure can be found in https://zenodo.org/record/8410965. (TIF) [file pbio.3002365.s005.tif]

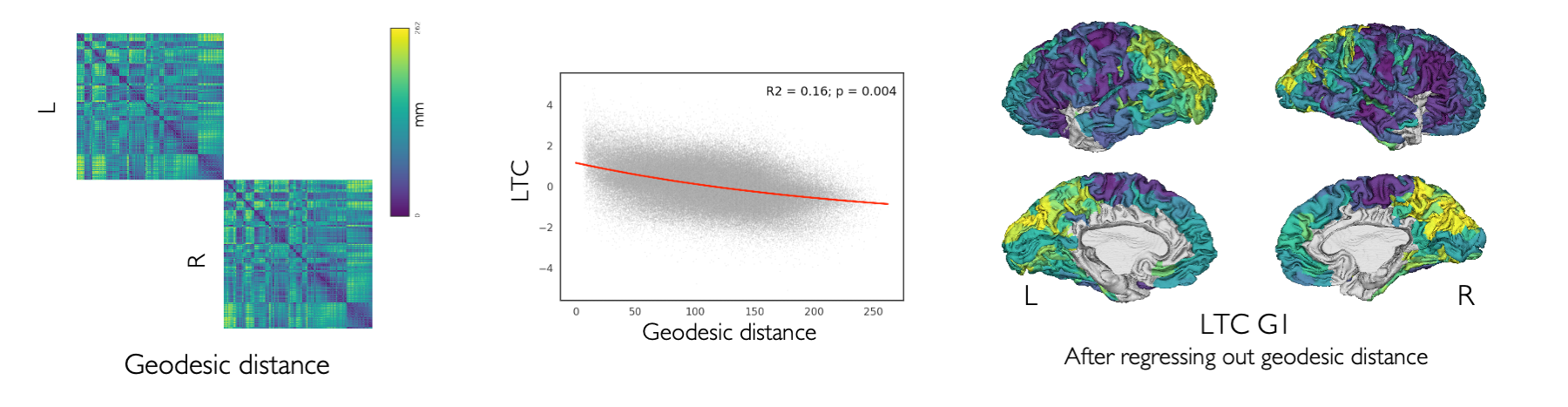

Supplement: S6 Fig — Geodesic distance (left) showed an inverse exponential relationship with the laminar thickness covariance, indicating similar laminar thickness patterns between neighbor regions (center). The principal axis of LTC (LTC G1) after regressing out the effects of geodesic distance (right) was significantly correlated with the original LTC G1 (r = 0.97, pvariogram < 0.001), indicating robustness of LTC G1 to geodesic distance. The data and code needed to generate this figure can be found in https://zenodo.org/record/8410965. (TIF) [file pbio.3002365.s006.tif]

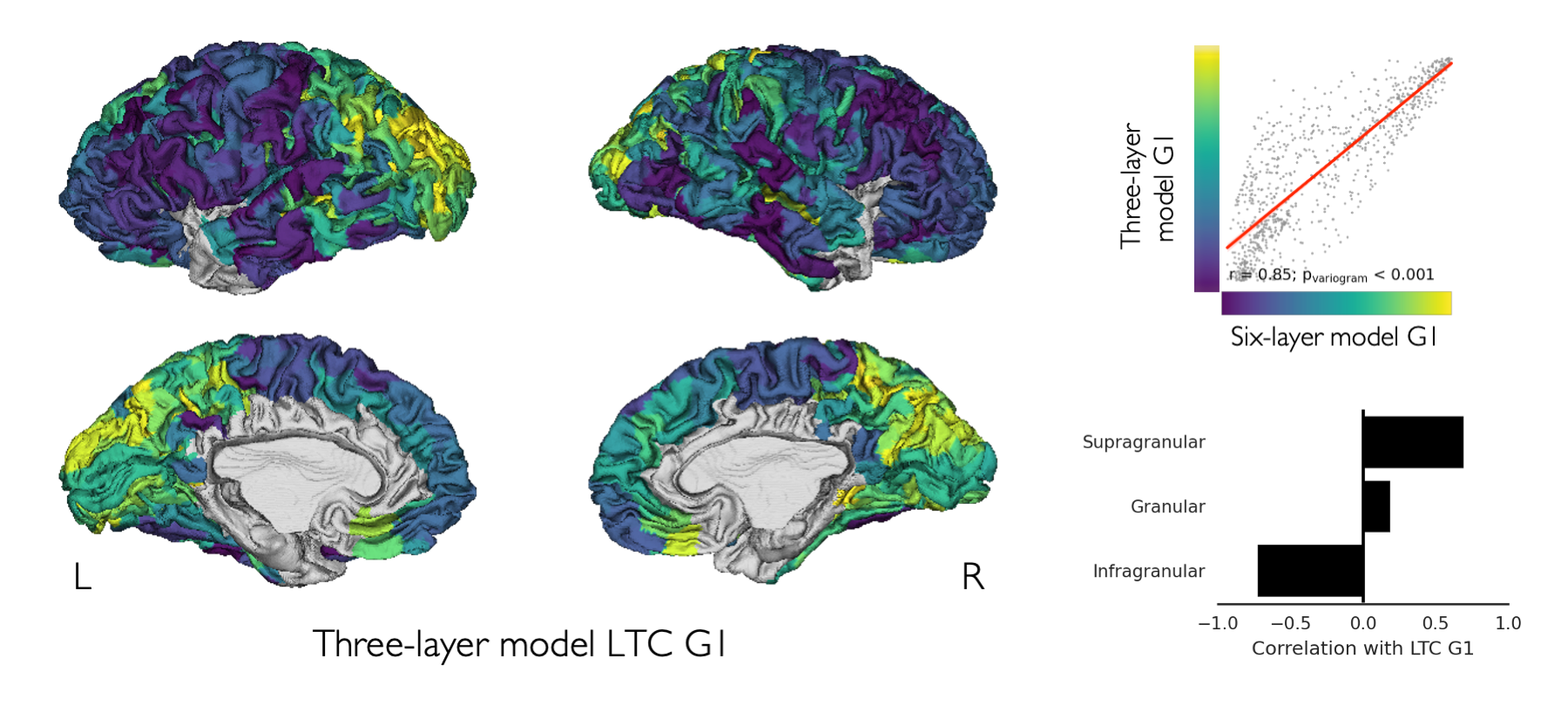

Supplement: S7 Fig — The principal axis of laminar thickness covariance (LTC G1) created using a 3-layer model including supragranular (I-III), granular (IV), and infragranular (V-VI) layers was correlated with the original 6-layer model LTC G1 and similarly described a shift from the dominance of infragranular to granular and supragranular layers. The data and code needed to generate this figure can be found in https://zenodo.org/record/8410965. (TIF) [file pbio.3002365.s007.tif]

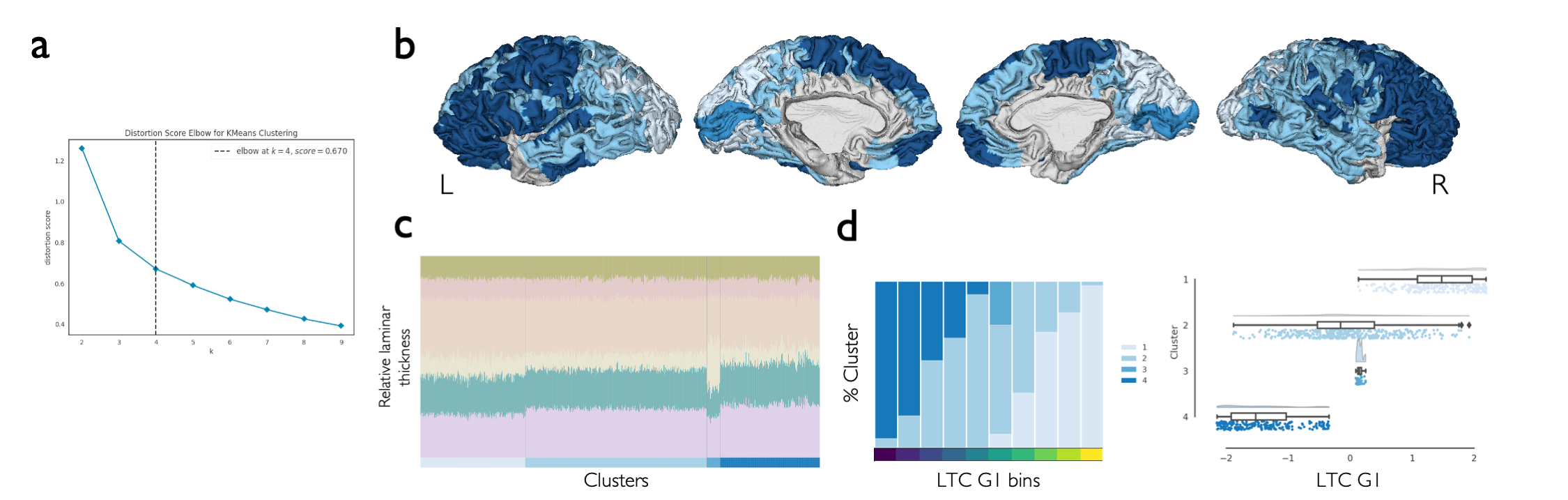

Supplement: S8 Fig — (a) The distortion score of K-means clustering for the different number of clusters. The optimal number of clusters based on the elbow method was selected as 4. (b) Cluster of regions based on relative laminar thickness. (c) Laminar thickness profiles of brain regions in each cluster. (d) The principal axis of laminar thickness covariance (LTC G1) values were significantly different between the clusters (F = 813.1, pspin < 0.001). Post hoc spin tests (Bonferroni corrected) showed significantly different LTC G1 values between all pairs of clusters except 2 and 3. The data and code needed to generate this figure can be found in https://zenodo.org/record/8410965. (TIF) [file pbio.3002365.s008.tif]

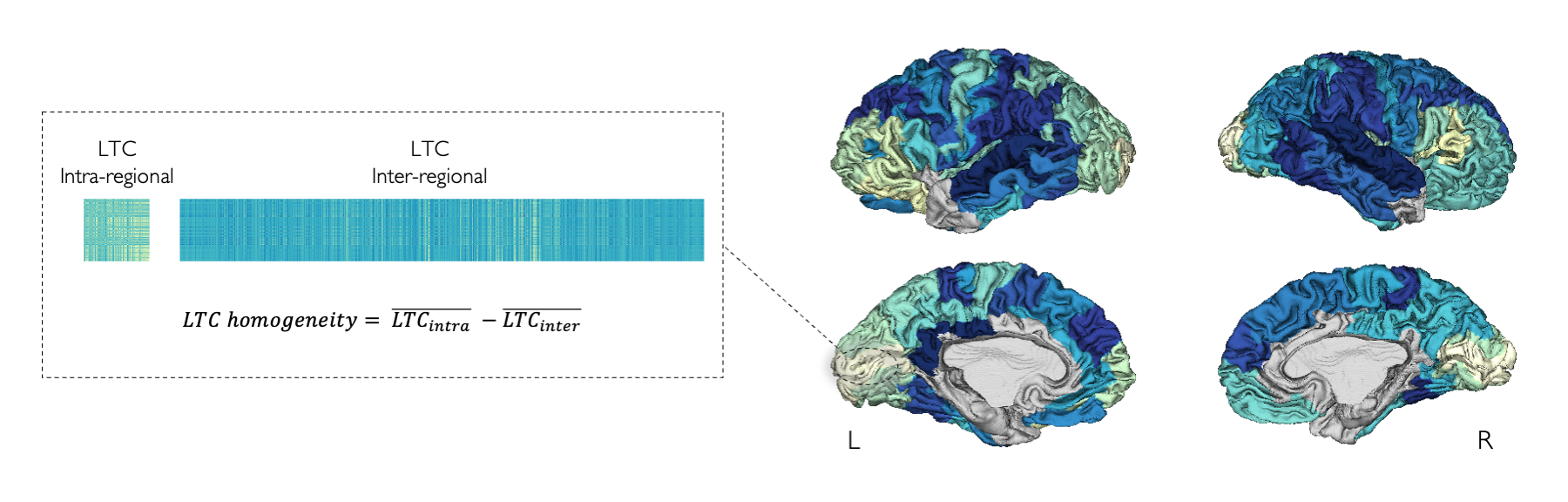

Supplement: S9 Fig — Laminar thickness covariance (LTC) homogeneity was calculated as the difference of average LTC between vertices that belong to the same region (LTCintra) versus other regions (LTCinter). Here, we used Brodmann areas as the map of cortical regions. The data and code needed to generate this figure can be found in https://zenodo.org/record/8410965. (TIF) [file pbio.3002365.s009.tif]

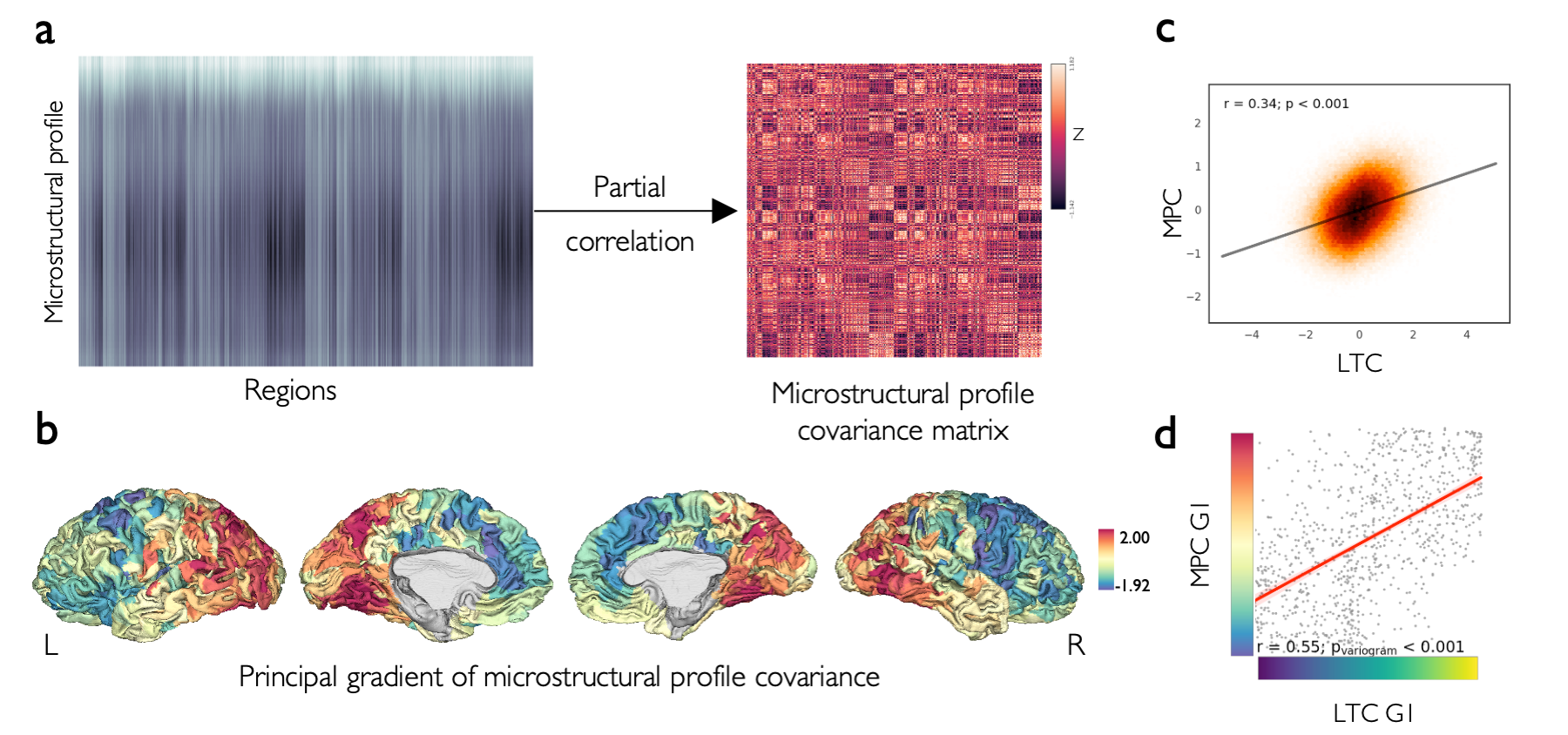

Supplement: S10 Fig — (a) The average regional microstructural profiles show variations of BigBrain image intensity across cortical depth (50 samples). Microstructural profile covariance (MPC) matrix was created by the pairwise partial correlation of intensity profiles between the parcels. (b) The principal axis of MPC created using principal component analysis. (c) The correlation between MPC and laminar thickness covariance (LTC) matrices. (d) The correlation between main axes of LTC and MPC. The data and code needed to generate this figure can be found in https://zenodo.org/record/8410965. (TIF) [file pbio.3002365.s010.tif]

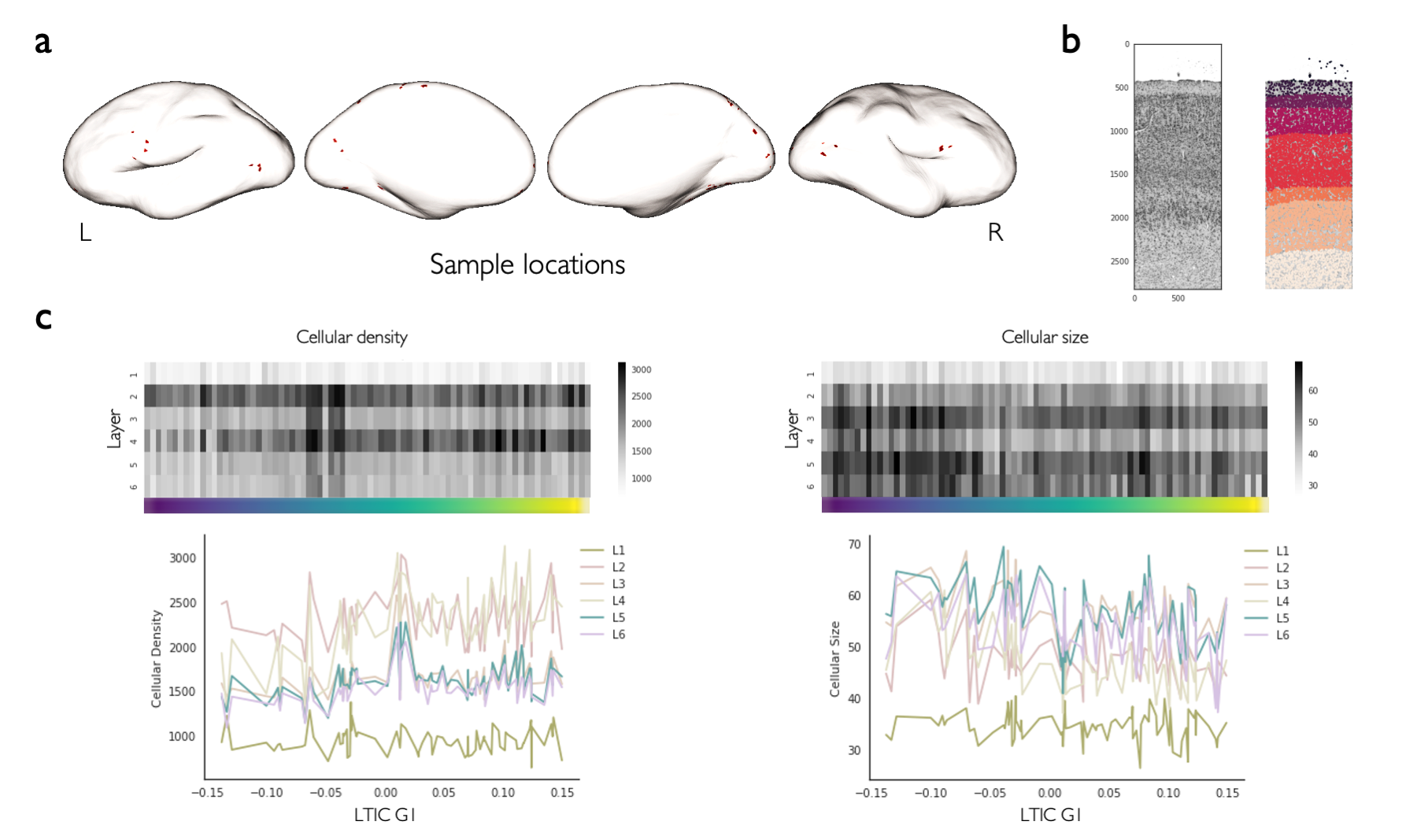

Supplement: S11 Fig — (a) Locations of cortical samples for which laminar cellular data were available. (b) Neuronal segmentation across cortical layers in an example sample. (c) Variation of laminar neuronal density and size along the principal axis of laminar thickness covariation among the available samples. The data and code needed to generate this figure can be found in https://zenodo.org/record/8410965. (TIF) [file pbio.3002365.s011.tif]

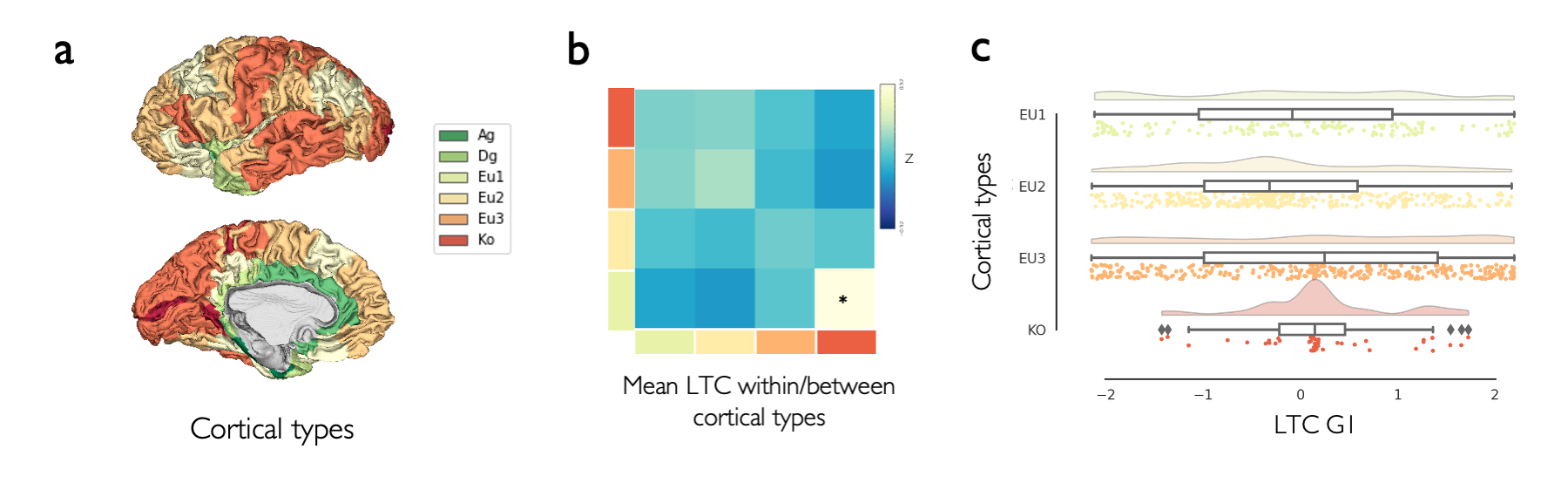

Supplement: S12 Fig — (a) The map of cortical types (left hemisphere) shows increasing laminar differentiation from agranular (green) to koniocortical (red) regions. (b) The average laminar thickness covariance (LTC) among pairs of parcels with the same or different cortical types, excluding agranular and dysgranular regions. Koniocortical regions showed significantly higher within-, compared to between-type average LTC. (c) Distribution of the principal axis of LTC (LTC G1) across the cortical types are shown in a raincloud plot. No significant difference in LTC G1 values was observed between the cortical types (F = 6.41, pspin = 0.633). The data and code needed to generate this figure can be found in https://zenodo.org/record/8410965. (TIF) [file pbio.3002365.s012.tif]

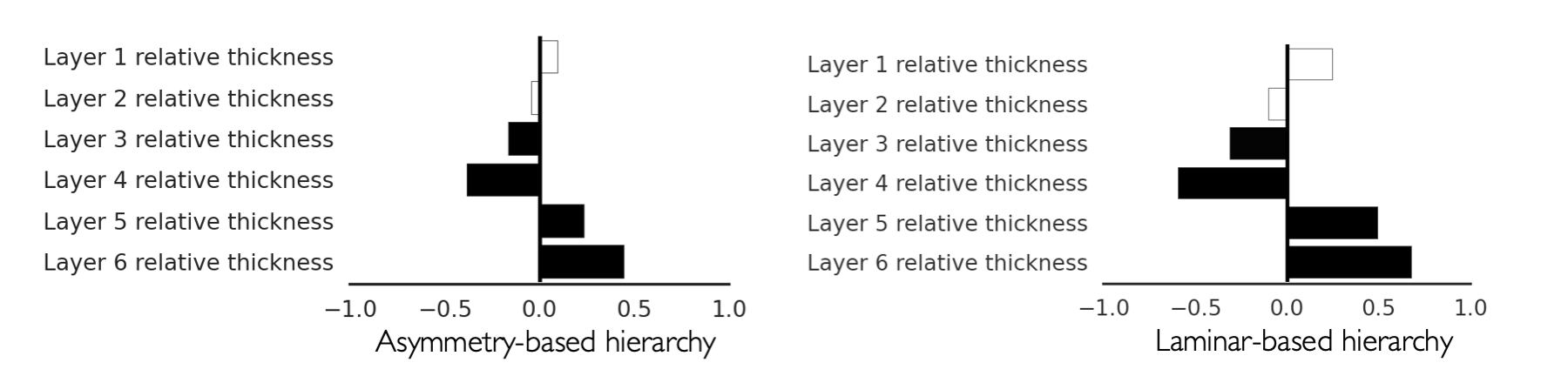

Supplement: S13 Fig — Bar length shows the correlation coefficient and its color represents the level of statistical significance from white (pvariogram, FDR > 0.05) to black (pvariogram, FDR < 0.001). The data and code needed to generate this figure can be found in https://zenodo.org/record/8410965. (TIF) [file pbio.3002365.s013.tif]

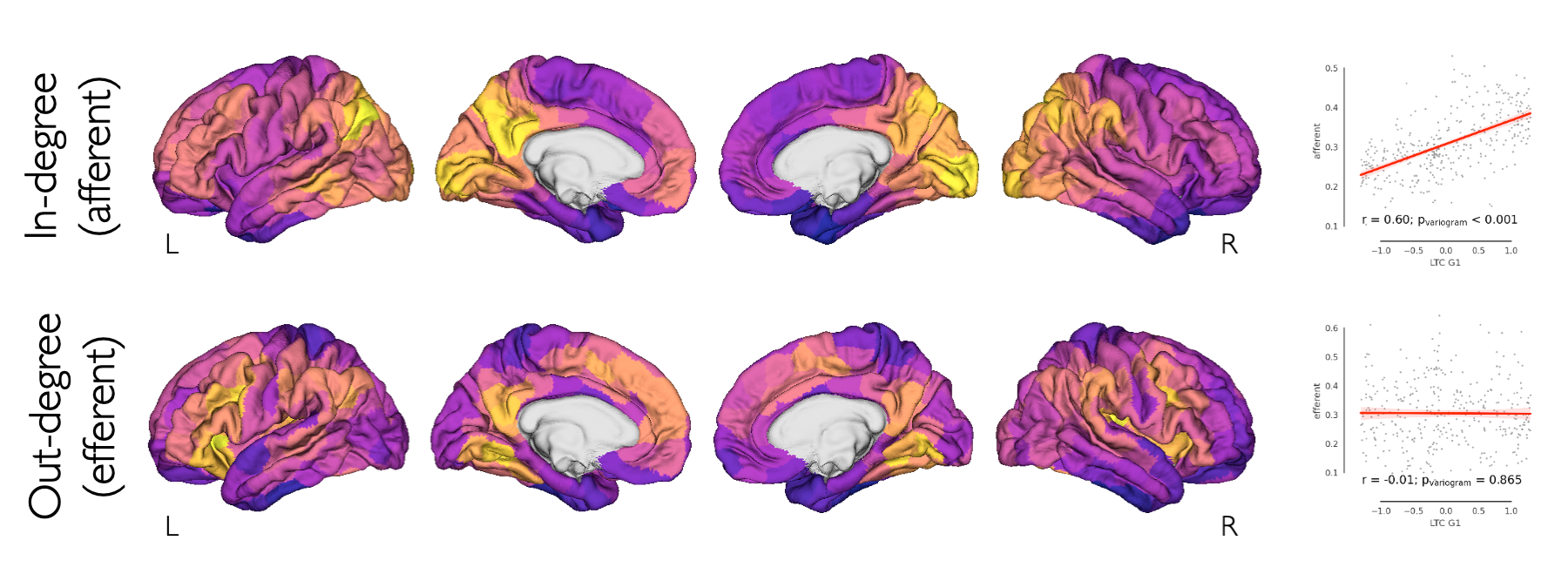

Supplement: S14 Fig — The principal axis of laminar thickness covariance (LTC G1) was significantly correlated with regional weighted in-degree (afferent strength) (top) but not weighted out-degree (efferent strength) (bottom). The data and code needed to generate this figure can be found in https://zenodo.org/record/8410965. (TIF) [file pbio.3002365.s014.tif]

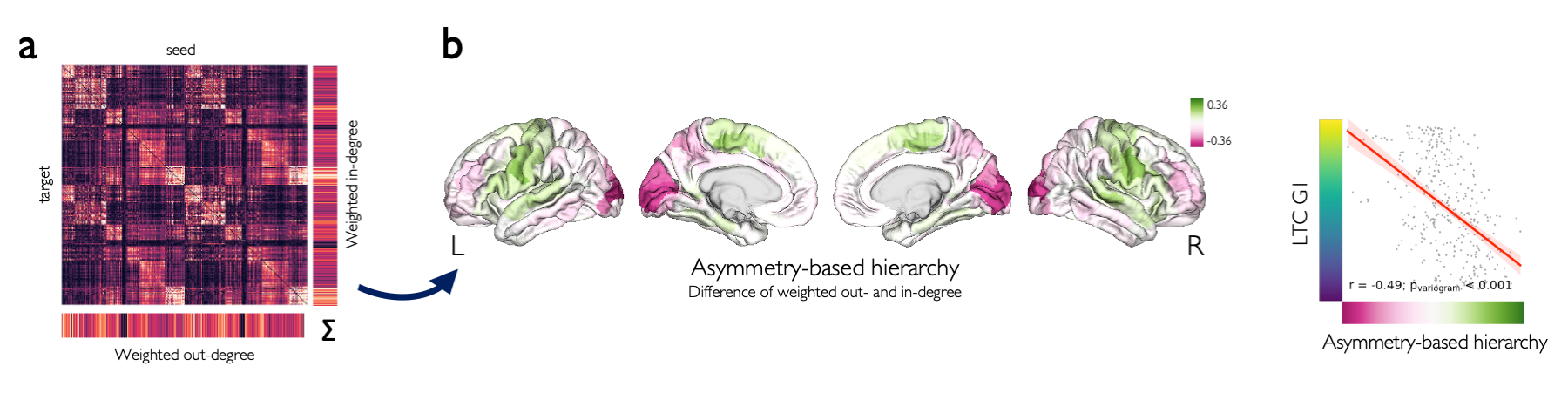

Supplement: S15 Fig — (a) The group-averaged effective connectivity matrix of the replication sample (N = 100) based on regression dynamic causal modeling. (b) Regional asymmetry-based hierarchy was calculated as the difference between their weighted unsigned out-degree and in-degree and was significantly correlated with principal axis of laminar thickness covariance (LTC G1). The data and code needed to generate this figure can be found in https://zenodo.org/record/8410965. (TIF) [file pbio.3002365.s015.tif]

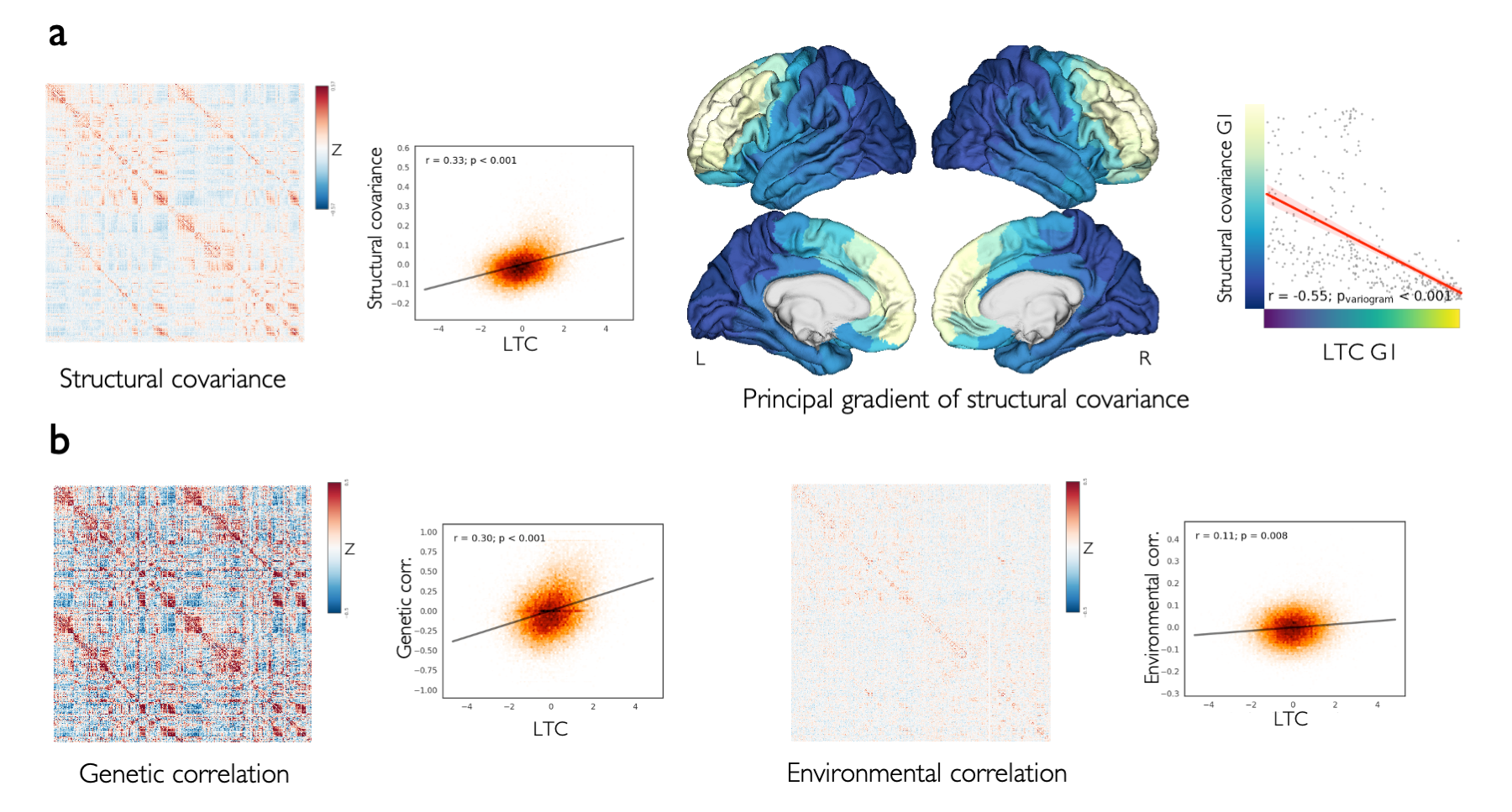

Supplement: S16 Fig — (a) The structural covariance matrix based on cortical thickness (left) in association with the laminar thickness covariance (LTC; center left). Main axes of structural covariance (center right) and LTC were correlated (right). (b) Interregional genetic and environmental correlation matrices based on cortical thickness in the HCP sample and their correlation with laminar thickness covariance. The data and code needed to generate this figure can be found in https://zenodo.org/record/8410965. (TIF) [file pbio.3002365.s016.tif]

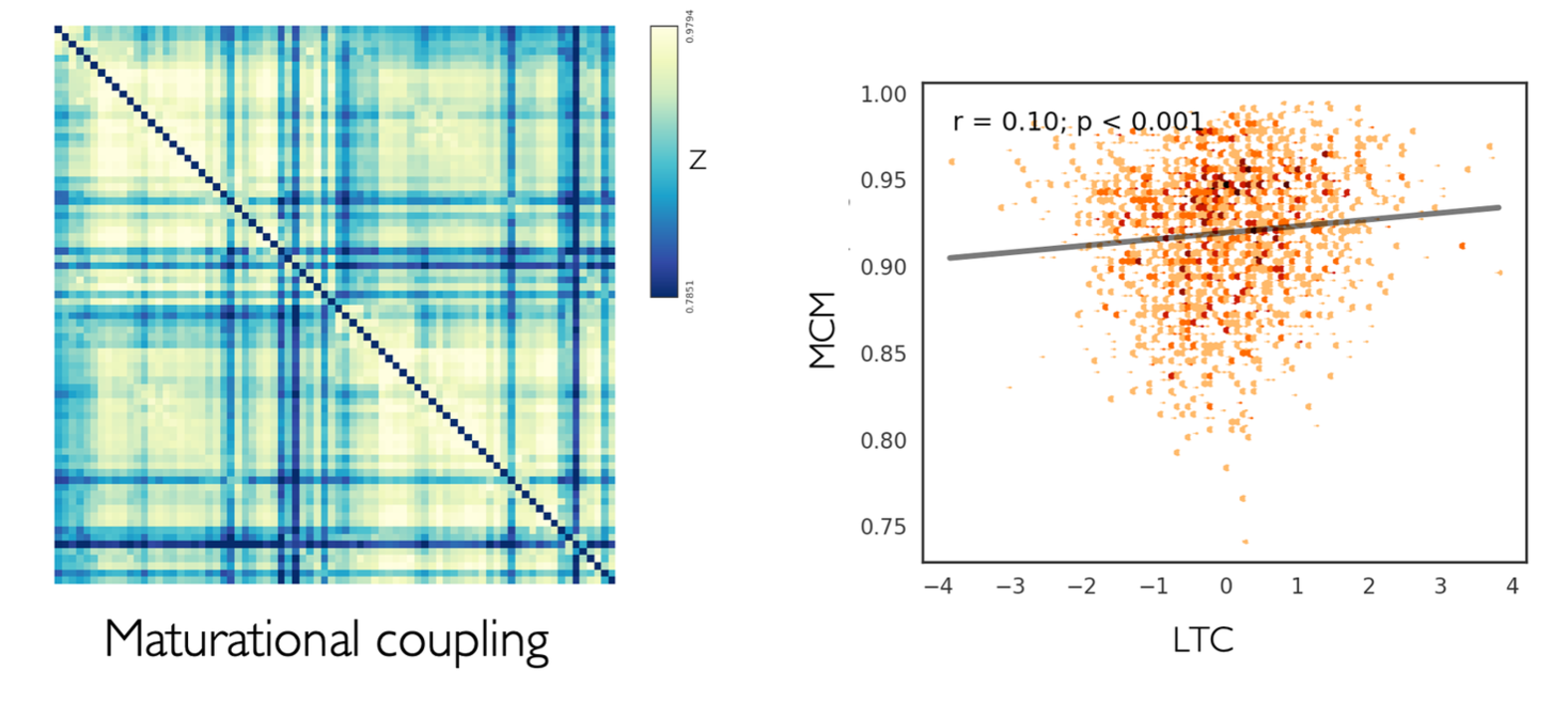

Supplement: S17 Fig — Maturational coupling matrix (MCM) was weakly associated with the laminar thickness covariance matrix (LTC). The data and code needed to generate this figure can be found in https://zenodo.org/record/8410965. (TIF) [file pbio.3002365.s017.tif]
