# Supplementary material for: The regional variation of laminar thickness in the human isocortex is related to cortical hierarchy and interregional connectivity
Source: PLoS Biol. 2023 Nov 9;21(11):e3002365. doi: 10.1371/journal.pbio.3002365 (PMC10684102; doi:10.1371/journal.pbio.3002365)
Supplement: S1 Text — (PDF) [file pbio.3002365.s018.pdf]

Text S1

Regional gene expression maps

Regional microarray expression data were obtained from 6 post-mortem brains (1 female, age = 24.0–57.0) provided by the Allen Human Brain Atlas (AHBA; https://human.brain-map.org) [1]. Data was processed with the *abagen* toolbox (https://github.com/rmarkello/abagen) [2] using the Schaefer-400 surface-based atlas in MNI space.

First, microarray probes were reannotated using data provided by Arnatkevičiūtė and colleagues [3]; probes not matched to a valid Entrez ID were discarded. Next, probes were filtered based on their expression intensity relative to background noise [4], such that probes with intensity less than the background in ≥ 50% or 25% of samples across donors were discarded. The threshold of ≥ 50% was used for correlated gene expression and developmental gene enrichment analyses, and ≥ 25% was used for creating transcriptomics maps of neuronal subtypes, as it led to removal of fewer genes associated with these subtypes. When multiple probes indexed the expression of the same gene, we selected and used the probe with the most consistent pattern of regional variation across donors (*i.e.*, differential stability) [5], calculated with:

$$\Delta_S(p) = \frac{1}{\binom{N}{2}} \sum_{i=1}^{N-1} \sum_{j=i+1}^{N} \rho\left[B_i(p), B_j(p)\right]$$

where ρ is Spearman's rank correlation of the expression of a single probe, p, across regions in two donors i and j, and N is the total number of donors. Here, regions correspond to the structural designations provided in the ontology from the AHBA.

The MNI coordinates of tissue samples were updated to those generated via non-linear registration using the Advanced Normalization Tools (ANTs; https://github.com/chrisfilo/alleninf). Samples were assigned to brain regions by minimizing the Euclidean distance between the MNI coordinates of each sample and the nearest surface vertex. Samples where the Euclidean distance to the nearest vertex was more than 2 standard deviations above the mean distance for all samples belonging to that donor were excluded. To reduce the potential for misassignment, sample-to-region matching was constrained by hemisphere and gross structural divisions (i.e., cerebral cortex, subcortex/brainstem, and cerebellum, such that *e.g.*, a sample in the left cerebral cortex could only be assigned to an atlas parcel in the left cerebral cortex) [3]. If a brain region was not assigned a sample from any donor based on the above procedure, the tissue sample closest to the centroid of that parcel was identified independently for each donor. The average of these samples was taken across donors, weighted by the distance between the parcel centroid and the sample, to obtain an estimate of the parcellated expression values for the missing region. All tissue samples not assigned to a brain region in the provided atlas were discarded.

Inter-subject variation was addressed by normalizing tissue sample expression values across genes using a robust sigmoid function [6]:

$$x_{norm} = \frac{1}{1 + \exp\left(-\frac{(x - \langle x \rangle)}{IQR_x}\right)}$$

where $<x>$ is the median and IQR_x is the normalized interquartile range of the expression of a single tissue sample across genes. Normalized expression values were then rescaled to the unit interval:

$$x_{scaled} = \frac{x_{norm} - \min(x_{norm})}{\max(x_{norm}) - \min(x_{norm})}$$

Gene expression values were then normalized across tissue samples using an identical procedure. Samples assigned to the same brain region were averaged separately for each donor and then across donors, yielding a regional expression matrix. We then excluded the right hemisphere regions due to the sparsity of samples and large number of regions with no expression data.

Transcriptomics maps of excitatory and inhibitory neuronal subtypes

The RNA sequencing of isolated cortical neuronal nuclei and their data-driven clustering has previously identified 16 neuronal subtypes, consisting of eight excitatory and eight inhibitory subtypes [7]. We used the list of subtype-specific genes and estimated the spatial distribution of each subtype by taking the weighted average mRNA expression of genes associated with the subtype, weighting each gene by the fraction of its positive values using exon-only derived transcripts per million [8]. Of note, few of the genes associated with each neuronal subtype had no eligible expression data in the processed AHBA dataset and were omitted from the calculation of subtype-specific gene expression (Table I).

We observed significant correlations of the principal axis of laminar thickness covariance (LTC G1), asymmetry-based hierarchy and laminar-based hierarchy maps with the spatial variation of excitatory and inhibitory neuronal subtypes based on gene expression (Fig I) [1,2,7]. This finding was in line with previous work on the association of cortical hierarchy and microstructure with cortical microcircuits [8–10].

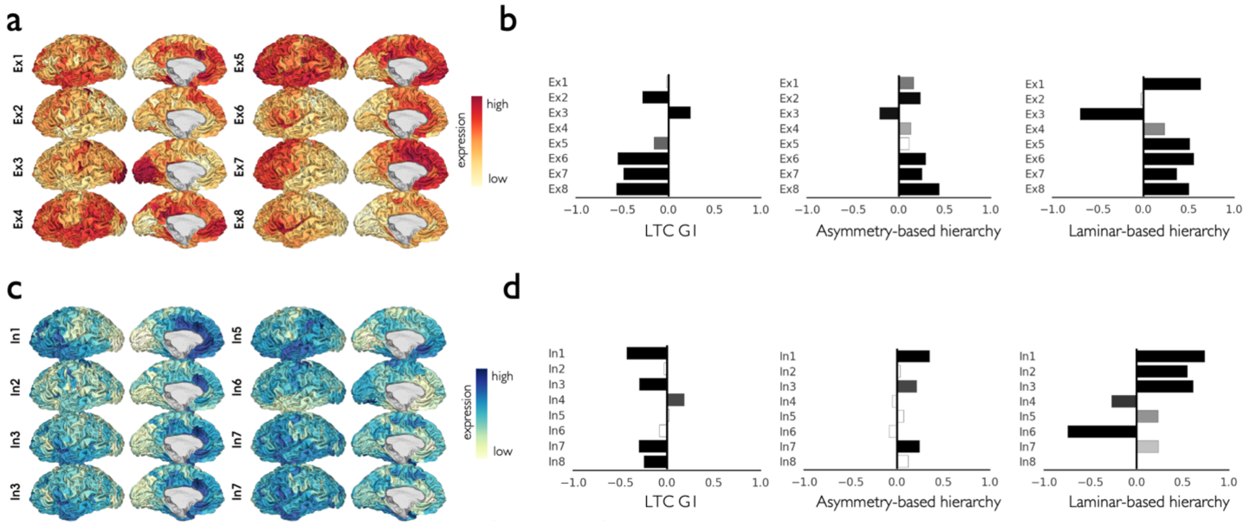

Fig I. Microcircuitry in association with cortical hierarchy and the principal axis of laminar thickness covariance. Spatial distribution of excitatory (**a**) and inhibitory (**c**) neuronal subtypes based on gene expression (left hemisphere), and their correlation with the principal axis of laminar thickness covariance (LTC G1) as well as asymmetry-based and laminar-based hierarchy (**b, d**). Bar length shows the correlation coefficient and its color represents the level of statistical significance from white ($p_{variogram,\ FDR} > 0.05$) to black ($p_{variogram,\ FDR} < 0.001$). The data and code needed to generate this figure can be found in https://zenodo.org/record/8410965.

Table I. Category and number of genes associated with each neuronal subtype.

Neuronal subtype	Category	Number of genes<br>*Total (excluded)*
**Excitatory**		
Ex1	*CPN*	14 (0)
Ex2	*GN*	9 (1)
Ex3	*GN*	4 (0)
Ex4	*SCPN*	12 (1)
Ex5	*SCPN*	8 (0)
Ex6	*SCPN*	33 (0)
Ex7	*CThPN*	3 (0)
Ex8	*CThPN*	57 (5)
**Inhibitory**		
In1	*VIP+ RELN+ NDNF+*	2 (0)
In2	*VIP+ RELN- NDNF-*	4 (0)
In3	*VIP+ RELN+ NDNF-*	12 (0)
In4	*VIP- RELN+ NDNF+*	6 (0)
In5	*CCK+ nNOS+ CB+*	7 (2)
In6	*PV+ CRHBP*	4 (0)
In7	*SOM+ CB+ NPY+*	6 (0)
In8	*SOM+ nNOS+*	1 (0)

CPN: cortical projection neuron, GN: granule neuron, SCPN: subcortical projection neuron, CThPN: corticothalamic projection neuron, SOM: somatostatin, NPY: neuropeptide Y, CB: calbindin-D-28k, VIP: vasoactive intestinal peptide, RELN: reelin, nNOS: neuronal nitric oxide synthase, PV: parvalbumin, CCK: cholecystokinin, NDNF: neuron-derived neurotrophic factor; CRHBP: corticotropin releasing hormone binding protein.

Correlated gene expression matrix

The correlated gene expression matrix was computed by correlating the regional gene expression patterns between the regions and across all genes, followed by its Z-transformation.

Further supporting shared genetic effects between regions with similar laminar thickness, we observed a correlation of $r = 0.24$ ($p_{spin} = 0.010$) between LTC and the correlated gene expression matrix. This matrix shows the correlation of gene expression between regions and across all genes, based on transcriptomics data obtained from the Allen Human Brain Atlas (AHBA; Fig II) [1,2].

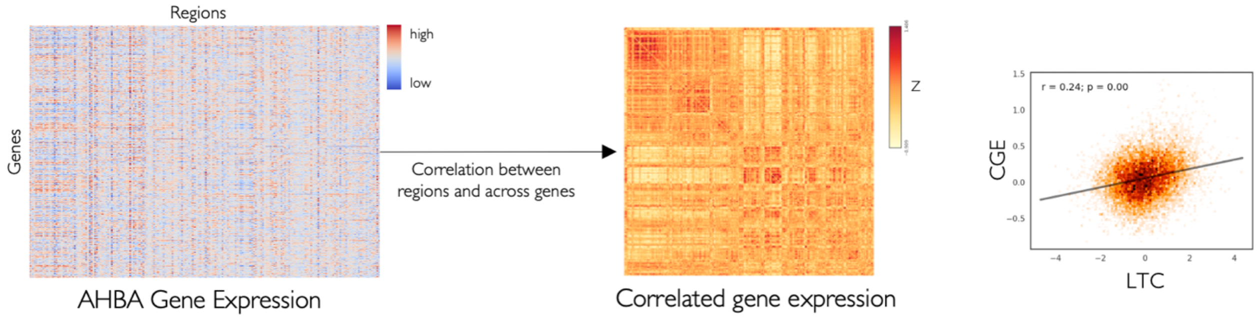

Fig II. Correlated gene expression in association with laminar thickness covariance. The gene expression data from Allen Human Brain Atlas (*left*) was used to create correlated gene expression (CGE) matrix, showing inter-regional similarity of gene expression profiles (*middle*), which was compared to the laminar thickness covariance matrix (LTC; *right*). The data and code needed to generate this figure can be found in https://zenodo.org/record/8410965.

Partial least squares regression of LTC G1 with gene expression maps and developmental enrichment analysis

We used partial least squares regression (PLS) to identify genes that show high alignment in their regional expression variability with the LTC G1 within the left hemisphere. The scikit-learn package (https://scikit-learn.org/stable/) [11] was used to perform single-component PLS between the parcellated map of LTC G1 on one side, and the regional expression of all genes on the other side. We then ranked the genes based on the absolute value of their weights and selected the top 500 genes, which were subsequently divided into genes with positive and negative weight. Next, we fed the list of top genes with positive and negative alignment to LTC G1 to the online cell-type specific expression analysis (CSEA) developmental expression tool (http://genetics.wustl.edu/jdlab/csea-tool-2/) [12], which involves comparison of the genes against developmental expression profiles from the BrainSpan Atlas of the Developing Human Brain (http://www.brainspan.org), and for each developmental stage and brain structure reported the inverse log of Fisher's Exact p-values corrected using Benjamini-Hochberg approach and based on the specificity index (pSI) threshold of 0.05.

We identified distinct sets of genes expressed preferentially at the opposite ends of the LTC G1 in adulthood based on the AHBA data, and investigated their developmental enrichment using the BrainSpan dataset [12]. We observed distinct spatiotemporal patterns for the expression of each set of genes associated with the rostral versus caudal ends of the LTC G1. Specifically, within the cerebral cortex, genes preferentially expressed at the caudal end of LTC G1 are mainly expressed in mid fetal and post-pubertal stages, while genes with higher expression at the rostral end of LTC G1 were expressed in late fetal and pre-pubertal stages of development (Fig III).

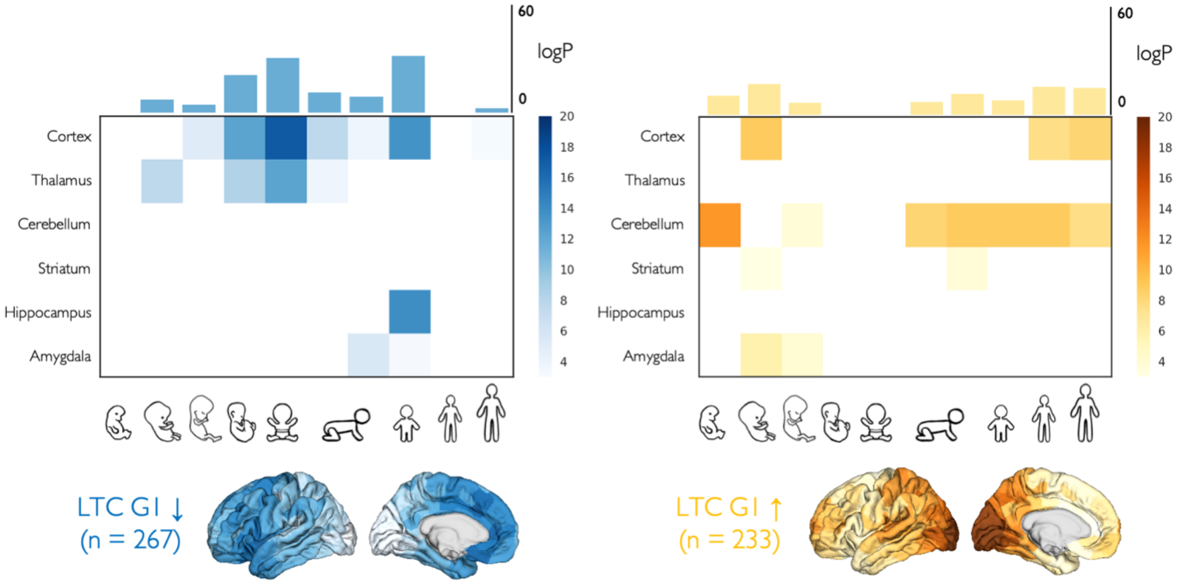

Fig III. Developmental enrichment of genes expressed in alignment with the principal axis of laminar thickness covariance. The average expression of top genes with regional expressions aligned to the principal axis of laminar thickness covariance (LTC G1), over-expressed rostrally (blue) or caudally (yellow) in the left hemisphere, and their spatiotemporal developmental enrichment. The data and code needed to generate this figure can be found in https://zenodo.org/record/8410965. The clip-arts are based on Ref. [13] published under CC BY 4.0 (https://creativecommons.org/licenses/by/4.0/).

References

1. Hawrylycz MJ, Lein ES, Guillozet-Bongaarts AL, Shen EH, Ng L, Miller JA, et al. An anatomically comprehensive atlas of the adult human brain transcriptome. Nature. 2012;489: 391–399. doi:10.1038/nature11405
2. Markello RD, Arnatkeviciute A, Poline J-B, Fulcher BD, Fornito A, Misic B. Standardizing workflows in imaging transcriptomics with the abagen toolbox. Jbabdi S, Makin TR, Jbabdi S, Burt J, Hawrylycz MJ, editors. eLife. 2021;10: e72129. doi:10.7554/eLife.72129
3. Arnatkevičiūtė A, Fulcher BD, Fornito A. A practical guide to linking brain-wide gene expression and neuroimaging data. NeuroImage. 2019;189: 353–367. doi:10.1016/j.neuroimage.2019.01.011
4. Quackenbush J. Microarray data normalization and transformation. Nat Genet. 2002;32 Suppl: 496–501. doi:10.1038/ng1032
5. Hawrylycz M, Miller JA, Menon V, Feng D, Dolbeare T, Guillozet-Bongaarts AL, et al. Canonical genetic signatures of the adult human brain. Nat Neurosci. 2015;18: 1832–1844. doi:10.1038/nn.4171
6. Fulcher BD, Little MA, Jones NS. Highly comparative time-series analysis: the empirical structure of time series and their methods. J R Soc Interface. 2013;10: 20130048. doi:10.1098/rsif.2013.0048
7. Lake BB, Ai R, Kaeser GE, Salathia NS, Yung YC, Liu R, et al. Neuronal subtypes and diversity revealed by single-nucleus RNA sequencing of the human brain. Science. 2016;352: 1586–1590. doi:10.1126/science.aaf1204
8. Burt JB, Demirtaş M, Eckner WJ, Navejar NM, Ji JL, Martin WJ, et al. Hierarchy of transcriptomic specialization across human cortex captured by structural neuroimaging topography. Nat Neurosci. 2018;21: 1251–1259. doi:10.1038/s41593-018-0195-0
9. Dombrowski SM, Hilgetag CC, Barbas H. Quantitative architecture distinguishes prefrontal cortical systems in the rhesus monkey. Cereb Cortex N Y N 1991. 2001;11: 975–988. doi:10.1093/cercor/11.10.975
10. Wang X-J. Macroscopic gradients of synaptic excitation and inhibition in the neocortex. Nat Rev Neurosci. 2020;21: 169–178. doi:10.1038/s41583-020-0262-x
11. Pedregosa F, Varoquaux G, Gramfort A, Michel V, Thirion B, Grisel O, et al. Scikit-learn: Machine learning in Python. J Mach Learn Res. 2011;12: 2825–2830.
12. Dougherty JD, Schmidt EF, Nakajima M, Heintz N. Analytical approaches to RNA profiling data for the identification of genes enriched in specific cells. Nucleic Acids Res. 2010;38: 4218–4230. doi:10.1093/nar/gkq130
13. Hettwer MD, Larivière S, Park BY, van den Heuvel OA, Schmaal L, Andreassen OA, et al. Coordinated cortical thickness alterations across six neurodevelopmental and psychiatric disorders. Nat Commun. 2022;13: 6851. doi:10.1038/s41467-022-34367-6
