# Supplementary material for: The regional variation of laminar thickness in the human isocortex is related to cortical hierarchy and interregional connectivity
Source: PLoS Biol. 2023 Nov 9;21(11):e3002365. doi: 10.1371/journal.pbio.3002365 (PMC10684102; doi:10.1371/journal.pbio.3002365)
Supplement: Attachment — Submitted filename: Response Letter.pdf [file pbio.3002365.s019.pdf]

Response letter

PBIOLOGY-D-23-00811R1, *The spatial arrangement of laminar thickness profiles in the human cortex scaffolds processing hierarchy*

We thank the Reviewers and editors for their thorough examination of our work, their helpful comments and questions, and the opportunity to submit a revised manuscript. We believe the article has significantly improved thanks to the revision. We have detailed our responses below as well as the changes in the manuscript.

In summary, as suggested by the Reviewers and the editors, we have put a stronger focus on our neuroanatomical investigations and findings, and de-emphasized our findings regarding the association of LTC with structural covariance and gene expression. We moved former Fig. 4 to Figs. S17, S18, and replaced it with a new main figure (Fig. 2), describing the association of laminar thickness variation in the BigBrain with laminar grey-matter density, neuronal density and soma size. We have performed additional analyses including the comparison of left and right hemispheres (Fig. S5) and evaluating intra-regional heterogeneity (Fig. S9). Moreover, we have rewritten parts of the Introduction and Discussion to i) reflect a more balanced view on interpretations of our findings and clearly indicate our speculations, ii) elaborate more on the issues of intra-/inter-regional heterogeneity, different number of layers and the exclusion of a-/dysgranular regions, iii) provide a clearer definition of "cortical hierarchy" and our interpretations of its relation to LTC G1, and iv) include a more diverse selection of citations.

Response to Reviewer #1

The present communication, from an established and expert group of investigators, sets out to quantitatively characterize co-variance of laminar thickness across 6-layered neocortical areas and further attempts to correlate results with ideas of hierarchical organization, genetic expression gradients, and developmental trajectories. I found the experimental results on laminar thickness trends interesting, novel, and significant, although I might have liked to see more detail and certainly more discussion; for example, about Fig. S6 which illustrates that results for three- and four-layer models are similar to the original six-layer model (now briefly described on page 6). That particular result strikes me as odd, as I might have considered a natural and more justified grouping to be the three-layer model, which separately distinguishes layer IV (a primarily input recipient and intrinsic layer). A main point, possibly problematic, is the focus on six-layers and the exclusion of agranular areas. Without further discussion and explication, this arguably detracts from the generality of the results. Some species have an exclusively pyramidalized cortex (as cetaceans; JM Graic... B. Cozzi, 2022, among others). Even within the six-layer scheme, as the authors will know, there are issues of sublamination, as well as significant intra-areal heterogeneity.

We thank the Reviewer for the appreciation of the work and the helpful and thoughtful comments and suggestions.

Indeed, in the current work we primarily focused on a six-layered model of the human cortex. This model is based on a previous work applying a convolutional neural network on histological images of BigBrain to segment cortical layers (Wagstyl et al., 2020). This model was trained to segment an equal number of six layers in all the regions. However, as the Reviewer also pointed out, the isocortex is not structured homogeneously in six layers, for example in regions with sublamination, or regions that may lack specific layers. Originally, we showed in our supplementary results that three- and four-layer models, created by merging selected conventional six layers, result in an LTC G1 map that is similar to the six-layer model. This model similarly characterizes a shift of infra- to supragranular dominance across LTC G1, which indicates sensitivity of LTC G1 to the changes of thickness in the supra- versus infragranular group of layers, more than individual layers (and potentially sub-layers) within each group. Of note, as the Reviewer has suggested, we have revised this supplementary analysis to only include the more conventional three-layer model of supragranular, granular and infragranular layers.

> *[Supplements, p 8]*
>
> 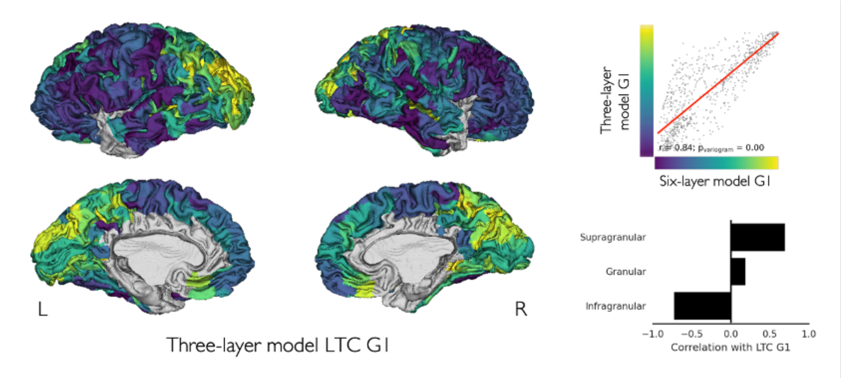
>
>
> Fig. S7. Principal axis of laminar thickness covariance using the three-layer model. The LTC G1 created using a three-layer model including supragranular (I-III), granular (IV) and

infragranular (V-VI) layers was correlated with the original six-layer model LTC G1 and similarly describe a infragranular to granular and supragranular transition.

On the other hand, in a- and dysgranular regions the layer boundaries are less clear and layer IV in particular is absent or rudimentary (García-Cabezas, Hacker, & Zikopoulos, 2020). In these regions assuming a six-layer model would not be accurate, and therefore, we excluded them from our analyses. However, as the Reviewer pointed out, doing so limits the generalizability of our results to the entire extent of the cerebral cortex. Nevertheless, we show that if we had included these regions, the LTC G1 would be consistent in the other regions (see Fig. S4b).

In the discussion, we had previously mentioned the issues of the different number of layers, as well as the exclusion of a-/dysgranular regions in the Limitations section. In the revised manuscript, we have elaborated more on these issues in a separate paragraph after we discuss our structural findings:

[Discussion, p. 13]
In the current work, we studied laminar thickness covariance using a six-layer model of the isocortex, previously created using a convolutional neural network [42]. However, it is well known that some isocortical areas have fewer or a greater number of layers, due to the individual layers being absent, or being divided into sublayers [1,4,82]. For example, area V1 is characterized by a prominent layer IV that is divided into three sublayers, and on the other hand, layer IV is unclear in agranular regions [4,14,82]. To avoid forcing a six-layer model in regions with fewer number of layers and less clear layer boundaries, we excluded a- and dysgranular regions from our analyses. Exclusion of these regions limits the generalizability of our findings to the whole extent of the isocortex, yet we showed that the LTC G1 map was consistent when these regions are included. In addition, to further explore the impact of a priori defined number of layers, we used a three-layer model of supragranular, granular and infragranular layers, and observed a similar principal axis. This indicates that LTC G1 captures variations of thickness in the supragranular, granular and infragranular layer groups rather than the individual layers within each group. Future research may account for the regional differences in the number of layers using more fine-grained models of intra-cortical structure where the number of layers in each location is determined based on the data rather than being fixed. This would enable formally testing the optimal architecture of cortical depth and enables inclusion of a-/dysgranular areas in a more comprehensive model of laminar structure in the cerebral cortex.

For heterogeneity, there are well known examples based on callosal or acallosal zones, and topographic specializations (foveal vs. peripheral field representatiuon in V1) among others, Increasingly, areas are acknowledged to have significant intra-areal heterogeneity (e.g., D. Haenelt... Tootell...2023 https://elifesciences.org/articles/78756 for myelin; E.M. Gordon et al., https://pubmed.ncbi.nlm.nih.gov/37076628/ 2023 for parallel processing (but: motor cortex); J. Gomez, Zhen, Weiner https://pubmed.ncbi.nlm.nih.gov/34618233/ 2021 for transcriptomics and visual field eccentricity, but relevant for laminar thickness comparisons). Can the Authors comment on how intra-areal heterogeneity may influence their results and their interpretation of inter-area interactions? If they are relying on an averaging approach, more careful description of the resulting strengths and limitations would be helpful, even in the Results, since this seems an important point.

Indeed, intra-areal heterogeneity may influence the results to some extent as we smooth, average across parcels and take a global covariance approach. In our results, we have shown that the LTC G1 spatial pattern was consistent ($r = 0.92$, $p_{spin} < 0.001$) when non-averaged data (at the vertex level) was used to construct the principal axis of LTC (Fig. S4a, vertex-wise map). This suggests sensitivity of LTC G1 to broader inter-regional variations rather than intra-areal variations.

To further study the extent of intra-areal variations in different regions, we performed an additional explorative analysis. Here, for every region defined using the Brodmann map, we calculated the average LTC of all vertex pairs within the same region, as well as pairs with other regions, and quantified intra-regional heterogeneity of laminar thickness as the difference of average within- versus between-region LTC. We chose this approach over calculation of variance of laminar thickness values in each region, to account for variability of all the layers in a single metric. We reported this analysis as follows:

[Results, p. 5]
Last, we quantified intra-regional homogeneity of laminar thickness patterns as the difference of intra- versus inter-regional vertex-level LTC across Brodmann areas. We observed high intra-regional homogeneity of laminar thickness in areas such as BA17, BA45 and BA47, in contrast to a high heterogeneity in areas such as BA22 and BA23 (Fig. S9).

[Methods, p. 23]
Intra-regional heterogeneity
The vertex-level LTC matrix was calculated in the downsampled *bigbrain* surface by using vertex-level smoothed laminar thickness patterns. Next, for every region, defined using the Brodmann map, we calculated the average LTC of all vertex pairs within the same region, as well as pairs with other regions. We quantified intra-regional heterogeneity of laminar thickness as the difference of average within- versus between-region LTC.

[Supplements, p. 10]

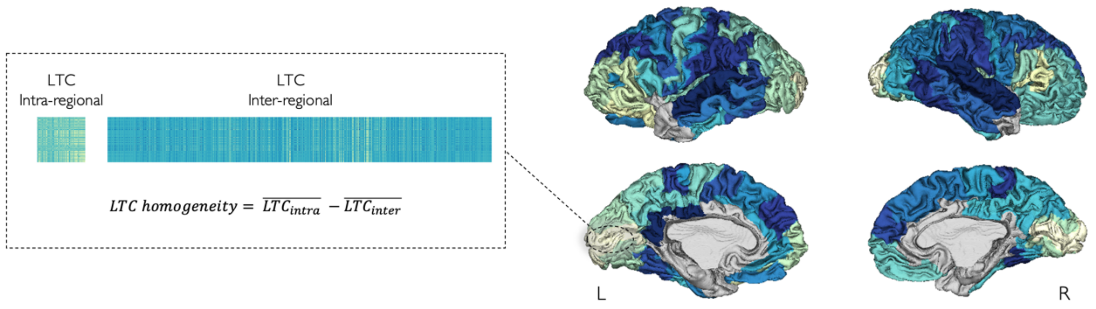

Fig. S9. Intra-regional homogeneity of laminar thickness covariance. Laminar thickness covariance (LTC) homogeneity was calculated as the difference of average LTC between vertices that belong to the same region (LTC_{intra}), versus other regions (LTC_{inter}). Here we used Brodmann areas as the map of cortical regions.

This finding shows the extent of intra-regional homogeneity of laminar thickness patterns in each region. However, from this result the spatial pattern of changes within each region is unclear, which can occur due to the sharp borders or more gradual changes inside the regions. It would be interesting to further explore this in a future study to characterize local transitions

and borders of laminar structure using a data-driven approach while also controlling for the effects of cortical folding.

In the revised discussion, we have included the additional findings, elaborated more on the issue of intra-regional heterogeneity of laminar structure (also mentioning suggested examples of intra-regional heterogeneity observed with other modalities), and suggested it as an interesting and relevant topic for future research:

> *[Discussion, p. 13]*
> Over the past century, there has been a debate over the optimal approach and level of granularity to study cytoarchitectural variability of the cerebral cortex [1,78]. Previous studies have ranged from focusing on fine cytoarchitectural details and identification of sharp borders between regions [2,4] to classification of the cerebral cortex into broader categories with grossly comparable cytoarchitecture [4,14]. On the other hand, some authors have argued against cortex-wide existence of sharp boundaries and rather focused on the gradual variations across the cerebral cortex [5,78]. We should note that here we refrained from making any assumptions on the (non)existence of sharp borders or a level of granularity as we aimed to provide a whole-cortex layer covariance organizational axis. We argue that the topology of cytoarchitectural variability of the cerebral cortex ranges from abrupt to more gradual changes [1,78]. Accordingly, the LTC G1 map consisted of a combination of sharp borders and gradual transitions, but was more dominated by gradual changes. We observed the LTC G1 map was consistent regardless of whether laminar thickness data was averaged into parcels or was analyzed at the level of vertices. This highlights that LTC G1 captures broader variations of laminar thickness across regions, in contrast to the finer local and intra-regional variations. Focusing on the local variations, we observed a varying level of intra-regional heterogeneity of laminar thickness across regions, as quantified by the average within- vs between-regional LTC. Specifically, primary visual area and orbital parts of inferior frontal gyrus were most homogeneous structures whereas regions in temporal and parietal lobes showed high heterogeneity of laminar thickness. Indeed, recent work is increasingly showing patterns of intra-regional cortical heterogeneity such as stripes of differential myelination in V2 [79], inter-effector areas in M1 [80] or differential gene expression in V1 associated with cortical layout of eccentricity [81]. Future work may uncover the spatial pattern and nature of such intra-regional heterogeneities in laminar structure and use data-driven approaches to study the organization of borders and abrupt alterations of layer thickness and associated cytoarchitecture variation.

Similarly, it would be interesting to have more comparison with other approaches, such as results from receptor distribution, which might seem at first glance to give a different picture of organization.

In this work we have focused on the thickness of cytoarchitecturally-defined layers as one of the several features of laminar structure. Indeed, we agree that investigating alternative measures and modalities, including laminar receptor distributions, can provide additional insights into the organization of laminar structure. In fact, a recent study by Goulas et al. (Goulas et al., 2021) used the laminar distribution of receptors as measured via autoradiography (Zilles & Palomero-Gallagher, 2017), and used PCA to identify a sensory-association axis of receptor variability. In the revised manuscript we have discussed this relevant study, further expanding our discussion on the distinct laminar features:

> *[Discussion, p. 13]*

… Beyond cytoarchitecture, additional features such as myeloarchitecture and receptor architecture vary across regions and such changes may be distinct from cytoarchitectural variation of laminar structure [74]. A recent study on the large-scale variation of layer-wise receptor densities in the human cerebral cortex based on autoradiography [75] reported a 'natural axis' of receptor distribution [76]. This axis spanned from association areas with higher infragranular AMPA density towards sensory areas with pronounced supragranular NMDA density as well as a higher diversity of receptor densities which was more prominent in infragranular layers. …

In addition, inspired by this comment, and to further highlight our structural findings, we merged former supplementary figures S9 and S10 to a new main figure (Fig. 2), which shows the variation of additional cytoarchitectural laminar features across the cortex as available in the BigBrain data, including layer-specific grey-matter density (in all vertices), and neuronal density and soma size (in a few selected locations):

[Results, p. 6]

Laminar thickness covariation with laminar neuronal density and size

Having characterized the spatial variation of laminar thickness patterns, we next studied its association with layer-/depth-wise measures of image intensity, neuronal density, and neuronal size in the BigBrain, as well as a map of cortical types which is a theory-driven map of laminar structure. By doing so we aimed to understand how laminar thickness covaries with additional cytoarchitectural features of laminar structure captured using data- and theory-driven approaches.

Microstructural profile covariance (MPC) is based on the image intensity profiles in the BigBrain cerebral cortex and is a data-driven model of cytoarchitecture that is explicitly agnostic to layer boundaries [10]. MPC was significantly correlated with our model of laminar thickness covariation, at the level of matrices ($r = 0.34$, $p_{spin} < 0.001$) and their principal axes ($r = 0.55$, $p_{variogram} < 0.001$) (Fig. S10). Extending this approach to the individual layers, we calculated layer-wise intensity profiles of the BigBrain cerebral cortex as the image intensity sampled at ten equivolumetric surfaces across each layer's depth, which we then averaged across the samples. Next, we calculated laminar intensity covariance (LIC) and applied PCA on the fused matrices of LTC and LIC, as a model of laminar structure covariation which took both laminar thickness and laminar density into account. The principal axis of the laminar thickness and intensity covariance (LTIC G1) was significantly correlated with LTC G1 and showed a similar pattern ($r = 0.84$, $p_{variogram} < 0.001$) (Fig. 2a). Along the LTIC G1, from rostral to caudal regions, we observed significantly increased grey-matter density of all the layers with layer IV showing the strongest effect ($r = 0.74$, $p_{variogram} < 0.001$) (Fig. 2b). The image intensity in the cell-body-stained BigBrain atlas reflects an aggregate of neuronal size and density, and at a resolution of 20 μm as individual neurons cannot be readily distinguished, these components cannot be disentangled. To further explore variations of neuronal size and density separately, we leveraged on a preliminary dataset of layer-wise neuron segmentations based on higher-resolution (1 μm) 2D patches from selected cortical regions of the BigBrain (Fig. S11a). We observed variation of laminar neuronal features along LTIC G1 which was most prominent in layer IV, showing increase of neuronal density ($rho = 0.54$, $p < 0.001$) and decrease of neuronal size ($rho = -0.62$, $p < 0.001$) (Fig. 2c). In addition, the ratio of average neuronal size in layer III to layer V, as a proxy for externopyramidization, was increased along LTIC G1 ($rho = 0.27$, $p = 0.01$; Fig. 2d). Last, we compared our data-driven model of laminar thickness covariation with the map of cortical types, a theory-driven model of laminar structural variation [14], and observed no significant association of the maps ($F = 6.41$, $p_{spin} =$

0.633) but significantly higher within-, compared to between-type average LTC in koniocortex (Fig. S12).

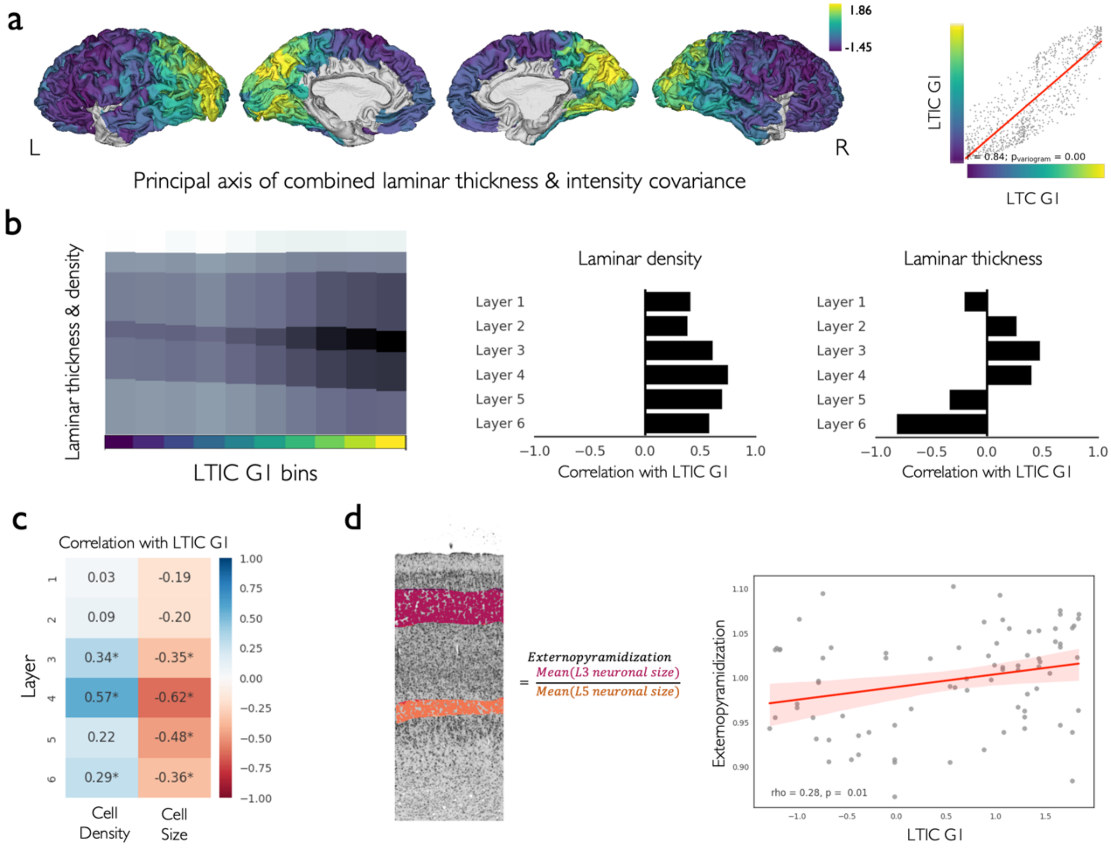

Fig 2. Laminar thickness covariation with laminar neuronal density and size. a) The principal axis of combined laminar thickness and intensity covariance matrices (LTIC G1) and its correlation with LTC G1. b) The pattern of changes in the thickness and density of the six layers along the LTIC G1. c) The correlation of laminar neuronal density and size along the LTIC G1 among the available samples (Fig. S11). d) The correlation of externopyramidization with the LTIC G1.

[Discussion, p. 12]

… In an integrated model of combined laminar thickness and intensity variations we observed a similar rostral to caudal principal axis characterizing increased grey-matter density in all the layers, most prominently in layer IV. …

In comparison with the core results on laminar thickness, the correlations with genetic expression and developmental trajectories are more derived from the literature and can seem more appropriate as speculations, although often presented as fact. In my opinion, the treatment of cell types was too broad and not well supported (Table S1), and the comparison with gene expression was necessarily inferential.

We appreciate the feedback and acknowledge that our findings regarding cell types and development are limited and should be interpreted and described accordingly. We have made several changes in the manuscript to clearly indicate when our interpretations are speculative. In addition, to further highlight our structural findings, we reduced the focus on the findings

related to maturation and gene expression (including the developmental trajectory and cell types). These changes include the following:

i. Former Fig. 4 is moved to supplementary figures S17 and S18, and the text in the Results is edited to reflect more accurate interpretations

[Results, p. 10]

Laminar thickness covariance in association to covariance and maturational coupling of cortical thickness

Thus far, we described how the laminar structure varies across the isocortex and its relevance to cortical hierarchy and connectivity. Lastly, we sought to study potential links of individual-level laminar thickness covariance to population-level inter-regional covariance and maturational coupling of cortical thickness using two complementary, yet indirect, approaches.

Structural covariance matrix reflects the pattern of covariation in cortical morphology (e.g., cortical thickness) across a population, which provides a model of shared maturational and genetic effects between cortical regions [45–47]. We obtained the structural covariance matrix based on the HCP dataset (N = 1113) from our previous work [47], and observed that it was significantly correlated with the LTC at the level of matrices ($r = 0.33$, $p_{spin} < 0.001$) and their principal axes ($r = -0.57$, $p_{variogram} < 0.001$). This may indicate shared maturational and genetic effects between regions with similar laminar thickness (Fig. S17a). Decomposing structural covariance into genetic (heritable, based on twin-modelling) and environmental (non-genetic + noise) components [47], we observed significant correlation of LTC with the inter-regional genetic ($r = 0.30$, $p_{spin} < 0.001$) but not environmental correlation (Fig. S17b). Further supporting shared genetic effects between regions with similar laminar thickness, we observed a correlation of $r = 0.20$ ($p_{spin} = 0.010$) between LTC and the correlated gene expression matrix. This matrix shows the correlation of gene expression between regions and across all genes, based on transcriptomics data obtained from the Allen Human Brain Atlas (AHBA; Fig. S17c) [36,37].

Last, we studied the association of LTC with the inter-regional maturational coupling matrix (MCM), obtained from a previous study by Khundrakpam and colleagues [52]. This matrix shows the similarity of regions in longitudinal cortical thickness changes over development in a dataset of children and adolescents (N = 140, baseline age = 11.9±3.6, followed up for ~2 years), and was weakly correlated with the LTC matrix ($r = 0.10$, $p_{spin} < 0.001$) (Fig. S18a). Furthermore, we identified distinct sets of genes expressed preferentially at the opposite ends of the LTC G1 in adulthood based on the AHBA data, and investigated their developmental enrichment using the BrainSpan dataset [48]. We observed distinct spatiotemporal patterns for the expression of each set of genes associated with the rostral versus caudal ends of the LTC G1. Specifically, within the cerebral cortex, genes preferentially expressed at the caudal end of LTC G1 are mainly expressed in mid fetal and post-pubertal stages, while genes with higher expression at the rostral end of LTC G1 were expressed in late fetal and pre-pubertal stages of development (Fig. S18b).

[Supplements, p. 18]

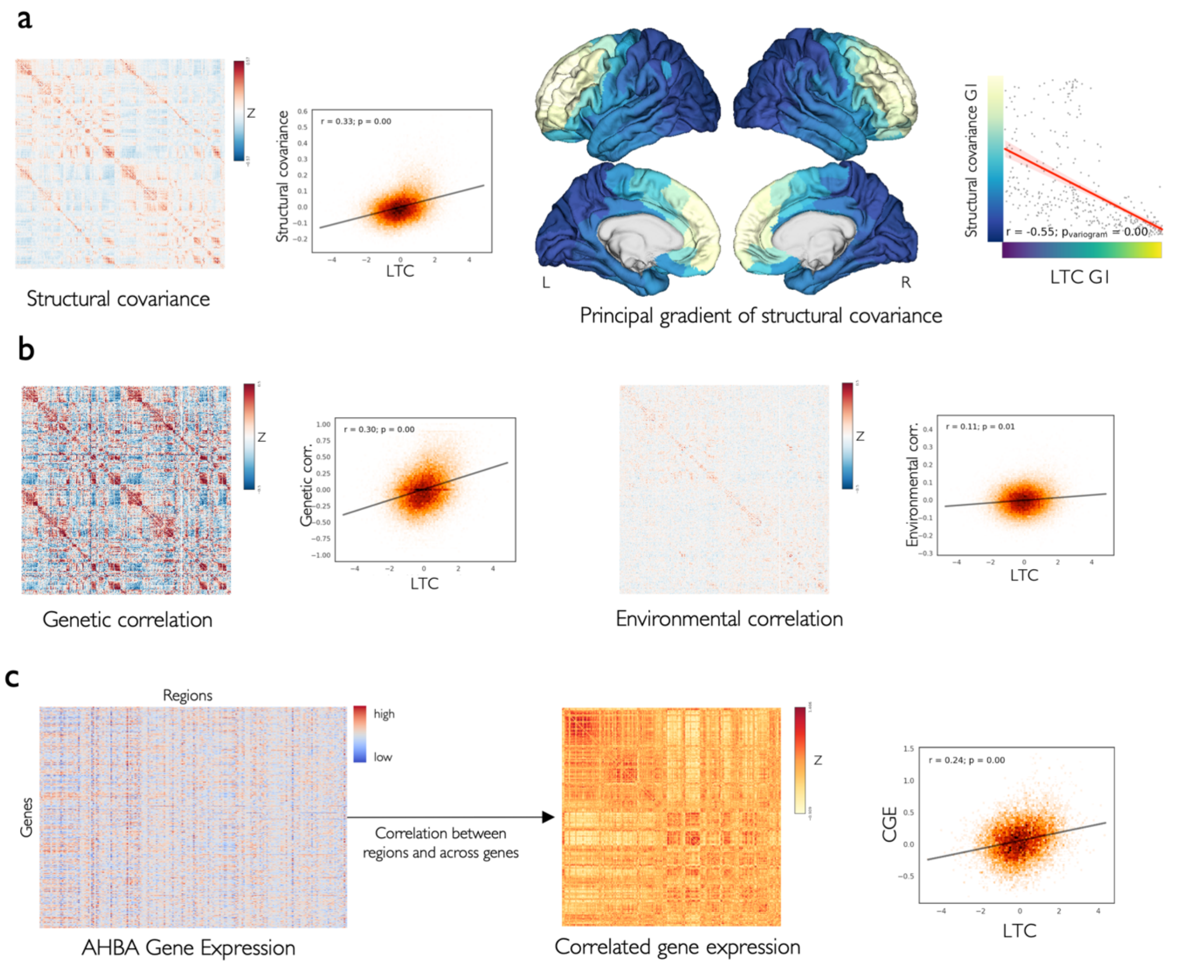

Fig. S17. Laminar thickness covariance in association to structural and gene expression covariance. a) The structural covariance matrix based on cortical thickness (left) in association with the LTC (center left). Main axes of structural covariance (center right) and LTC were correlated (right). b) Inter-regional genetic and environmental correlation matrices based on cortical thickness in the HCP sample and their correlation with laminar thickness covariance. c) The gene expression data from Allen Human Brain Atlas (AHBA) (left) was used to create correlated gene expression (CGE) matrix, showing inter-regional similarity of gene expression profiles (middle), which was compared to the laminar thickness covariance matrix (right).

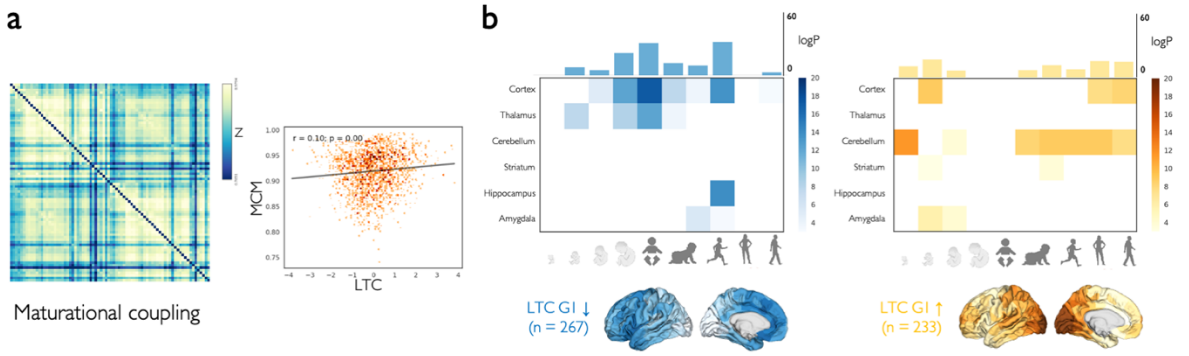

Fig S18. Maturational links to laminar thickness covariance. a) Maturational coupling matrix (left) was weakly associated with LTC (right). b) The average expression of top genes with

regional expressions aligned to LTC G1, over-expressed rostrally (blue) or caudally (yellow) in the left hemisphere, and their spatiotemporal developmental enrichment.

ii. The Introduction paragraph on the developmental hypothesis of laminar structure variation is removed and integrated in the Discussion. The Discussion paragraph on findings related to structural covariance, maturational coupling and transcriptomics is rewritten as follows:

[Discussion, p. 16]
We observed higher inter-regional LTC was linked to higher population-level inter-regional structural covariance, which potentially indicates shared genetic and maturational effects among regions [65,67]. In addition, we observed a significant but weak correlation of LTC with subject-level longitudinal maturational coupling of cortical regions during childhood and adolescence [66], and found distinct pre- and postnatal developmental trajectories of genes overexpressed at the two ends of LTC G1. We speculate that regional differences in gene expression and developmental trajectories may underlie regional variability of laminar structure. The hypothesis of different developmental trajectories relating to gradation of laminar structure has been previously put forward by Hilgetag, Barbas and colleagues [17,22,54]. There are regional differences in neurogenesis timing and cell-cycle duration throughout fetal development [108–114], or region- and layer-specific neuronal death in early postnatal stages [115] which may result in the specification of regions and their cytoarchitectural variability. However, our current findings can only indirectly suggest developmental relevance of laminar thickness organization. Further studies are needed to investigate this hypothesis by using postmortem histology or in-vivo markers of laminar structure [10,116,117] during pre- and postnatal development to characterize maturation of laminar structure at different stages of development and better understand the potential developmental mechanisms underlying regional variations in laminar structure.

iii. We agree that the findings reported on the excitatory and inhibitory cell types are too broad. We believe a more detailed account of these variations and their associations with laminar structure is interesting but beyond the scope of current work. In addition, a-/dysgranular regions are discussed as important anchors of cell type variation in previous literature (Dombrowski, Hilgetag, & Barbas, 2001), and yet, we had to exclude them for methodological considerations discussed above. Therefore, we have removed our previous Discussion paragraph on the findings pertaining to the cell types, though we continue to report it in the Results and the Supplements.

I also had some reservations about the relationship of the lamination results to "processing hierarchy." First, the concept and details of "hierarchy" are still very much in discussion and even contentious, as the Authors briefly state (page 8). They reference articles 59-63, but more current articles should be included; for example, Hilgetag and Goulas, 2020, Vezoli et al., 2021, and (comparing the structural model and distance model) Aparicio-Rodriguez and Garcia-Cabezas, 2023. The Authors evidently elect for the Structural Model, but further justification or at least fuller discussion of the several alternatives would be appropriate.

As the Reviewer pointed out, the term 'hierarchy' is defined in many different ways in the neuroscience literature (Hilgetag & Goulas, 2020), and here, we are focusing on the asymmetry-based and laminar-based definitions of 'hierarchy'. We have rewritten the

relevant paragraph in the Introduction to provide a clear definition of `hierarchy` early in the manuscript:

> *[Introduction, p. 3]*
> The laminar pattern and likelihood of cortico-cortical connections are suggested to relate to the inter-regional variation of cortical cytoarchitecture [3,17,18]. Connectivity is shown to be more likely between regions with similar cytoarchitecture [19–24]. In addition, the gradation of cytoarchitecture is suggested to predict the laminar pattern of cortico-cortical connections [3,17,18,25,26], categorized as 'feedback' (FB), 'feedforward' (FF) or 'lateral' based on tract-tracing data [27–29]. These laminar projections have in turn been used to describe an ordering of regions along a cortical hierarchy, in which FF projections are suggested to carry high-dimensional sensory information from lower to higher regions and are reciprocated by FB projections transmitting context and modulatory signals from higher to lower regions [29,30]. Recently, it was shown that a marker of cortical myelination (T1/T2w) was associated with the map of laminar-based hierarchy [27]. Together with findings on the association of cortical cytoarchitecture and laminar projections [3,17,25,26], this suggests a potential link between cortical microstructure and hierarchy. Yet, it is unclear how laminar thickness may scaffold connections within the cortical hierarchy. Notably, in neuroscience the term 'hierarchy' has been used to describe different phenomena [31], such as gradients of structural and functional features [27,32], topological sequence of connections [33], asymmetry of directional connections indicating inter-regional control or dominance [34,35], or as described above, the sorting of laminar projection patterns and their physiological correlates [28,29,36–41]. Throughout this paper we will focus on the latter two definitions of hierarchy, that is, laminar-based and asymmetry-based hierarchy.

As suggested, although the distance rule model was originally mentioned in the Discussion, we have elaborated more on it and included additional relevant references. In particular, we have also mentioned that the distance rule model has been used to explain the gradation of changes in laminar pattern of connections, in addition to the likelihood of connectivity:

> *[Discussion, p. 14]*
> … The laminar pattern of corticocortical connections is suggested to relate to the gradation of cortical microstructure (the 'structural model') [3,17,25,26] or the physical proximity of regions (the 'distance rule model') [28,29]. These models suggest that the laminar connections of cytoarchitecturally similar or proximal regions are mostly lateral, but the pattern of connections become increasingly FF/FB as regions are more dissimilar in cytoarchitecture or are more distant [3,17,25,28,29,83]. …
>
> *[Discussion, p. 15]*
> … An alternative model of connectivity is the 'distance rule model' which proposes physical proximity as the main predictor of connectivity as a result of wiring cost minimization [61,62,104–106]. It can be argued that the increased connectivity of similar regions may be an epiphenomenon of the distance rule, as nearby cortical regions tend to be similar [63,64]. However, it has been shown that the distance rule alone does not fully account for the connectome architecture. For example, simulated connectomes were shown to better resemble the empirical connectomes when inter-regional similarity was considered in addition to the wiring cost reduction [93]. Recent studies on tract-tracing data have shown that both similarity of cortical types and physical proximity can predict likelihood of structural connections [25,83], though in most species cytoarchitectonic similarity was related to connectivity, above and beyond physical proximity [83]. Nevertheless, in our study long-range connections were

> not significantly associated with similarity of laminar thickness profiles, suggesting distance as an important covariate. …

Second, the Authors frequently talk about "likelihood and strength of connections," but this also can seem inferential, given the non-straightforward homologies between human and macaque brains and again, the complexities of "strength." Similarly, page 8 (and elsewhere): "Feedforward connections transmit high-dimensional information..." [maybe better: are thought to transmit....]. The Authors may precisely be hoping to derive a predictive framework from their approach, but normal histology data are not adequate, and the extrapolated data from gene expression, development, and monkey tract tracing in my opinion, are not compelling. [on organization of default mode network: CM Garin et al (2022) https://doi.org/10.1016/j.celrep.2022.110669, among others.]

First, we agree that defining and measuring "strength" of connectivity, and in particular, structural connections, is a challenge. Therefore, in our comparison of LTC with the structural connectivity, we only focus on the likelihood of existence of connections in a thresholded matrix, rather than strength. However, in the functional connectivity matrix, the strength of connections can be defined as the degree to which regions are in synchrony and can be quantified by pairwise Pearson's correlation of time series. To make this clear, we have described our findings as association of LTC with the likelihood of structural and strength of functional connections, rather than the broad statement of "likelihood and strength of connectivity" without distinguishing structural and functional connectivity.

Second, there are indeed limitations in cross-species comparisons of humans with non-human primates given the structural and functional differences of the species (García-Cabezas, Hacker, & Zikopoulos, 2022; Garin et al., 2022; Xu et al., 2020). There is some evidence on a rostro-caudal infra- to supragranular shift in non-human primates (Charvet, Cahalane, & Finlay, 2015), as well as increased feed-back processing of anterior regions in humans (Huber et al., 2020; Vezoli et al., 2021), but without further formal comparisons, it is unclear how laminar thickness patterns or laminar-based hierarchy maps differ between humans and non-human primates. Nevertheless, we chose to do a cross-species comparison as the direct mapping of laminar connections is only possible via the invasive method of tract-tracing in animals, and yet, we were interested in its association with laminar structure in the context of previous research (Goulas, Zilles, & Hilgetag, 2018). Moreover, to additionally have human-based evidence on the association of laminar structure with cortical hierarchy, we used asymmetry-based hierarchy defined using effective functional connectivity, and observed similar effects. However, we acknowledge that this definition is different from laminar-based hierarchy (Goulas, Uylings, & Stiers, 2014; Hilgetag & Goulas, 2020). We have elaborated more on these issues in our Discussion and highlighted them as important questions that can be addressed in future research:

> *[Discussion, p. 14]*
> … In our comparison of LTC G1 with the laminar-based hierarchy map, we performed a cross-species comparison, yet we should note the limitations of this approach given the differences of humans and non-human primates in cortical cytoarchitecture [89] and connectivity [53,90]. There is some evidence based on cortical oscillations (c.f. above) and the pattern of intra-laminar connectivity estimated using layer-based functional magnetic resonance imaging [91], that indicate increased feedback dominance towards rostral regions in humans as well. Moreover, the human map of cortical hierarchy that we defined based on the asymmetry of effective connections, showed a similar association with LTC G1 as the macaque's laminar-

> based hierarchy. However, the definitions of asymmetry-based and laminar-based hierarchy are different [31] and may result in different maps, as was previously shown in the frontal cortex of macaques [35]. Layer-wise functional imaging is a promising approach that can be used to further investigate the association of laminar structure with the pattern of laminar connections and their functional implications in humans [91]. …

In summary, my recommendation is for more detailed emphasis on and discussion of the laminar thickness experimental data. Possible correlations with gene expression, and developmental trajectory might be better in a single section on "Speculations" or "Perspective" or "Future Directions." These are not in any case mentioned in the present title, although the Authors may have been aiming for a coherent and comprehensive framework.

As mentioned above, we have reduced the focus on our findings related to structural covariance, maturational coupling, and gene expression, in favor of increased emphasis on our structural findings and discussion. In addition, we have rewrote parts of the Introduction and Discussion to clearly indicate our speculations. However, we did not include these in a separate section, as the discussion is organized by the topic and we have discussed speculations/perspectives or future directions in their respective paragraphs. We have also modified the title to a more comprehensive one which captures our different findings, that is, "The spatial arrangement of laminar thickness profiles in the human isocortex scaffolds cortical organization".

The various analysis programs have been developed or extensively used by this Group or colleagues. Statistical analysis is important for this study, but I do not include this in my evaluation.

We appreciate the Reviewer's comment on statistical analysis and agree with its importance.

Other points:
1) The sulcal-gyral deformation is referred to as The "Bok principle" (1929), but the deformation was already illustrated by Economo and Koskinas (figure 30, 1925), possibly among others.

In the revised manuscript we have described this deformation without calling it the "Bok principle".

2) The cellular descriptors, top of page 2, seemed unconventional. The densely packed "granular cells" in layer II are mainly pyramidal, and not necessarily small; layer III is commonly described as having a size gradient from IIIa (small pyramids) to IIIc (larger pyramids), layer IV outside the primary areas has small pyramidal cells (e.g., R. Douglas et al. in Shepherd "The Synaptic Organization..." 2004, 5th edition: "the spiny stellate neurons are found exclusively in layer 4 of the granular cortex" (referencing Cajal); J.S. Lund et al., 1981, from Golgi specimens in monkey visual cortex, and DeFelipe 2011, figures 4 and 13), layer V is often subdivided as Va (smaller, intratelencephalic pyramids) and Vb (with scattered large pyramids), and Layer VI is not usually described (?) has having "spindle-shaped neurons."

As suggested, we have described the layers more accurately, as follows:

[Introduction, p. 3]
… From the pial to the gray-white matter interface, they include layer I which contains mostly dendrites and axon terminals and has a low cellular density, layers II and III which mainly contain pyramidal cells, with a size gradient in neurons of layer III that become larger towards its lower extent, layer IV which consists of densely packed small pyramidal and non-pyramidal neurons, layer V which is composed of pyramidal neurons which are small and intratelencephalic (layer Va) or large and sparse (layer Vb), and layer VI with corticothalamic pyramidal cells and heterogeneously-shaped neurons [2,4,12,13]. …

3) I may have overlooked this but are the data from both hemispheres? RH? LH? What about possible asymmetries?

Our main analyses on the BigBrain are based on the data from both hemispheres. However, in the revised manuscript we performed a new analysis to compare hemisphere-specific LTC G1 maps and showed that they are largely comparable ($r = 0.74$, $p_{variogram} < 0.001$), yet despite the overall alignment of the left and right LTC G1 maps, there are some asymmetries. A detailed investigation into the asymmetry of microstructure and laminar structure of the BigBrain is the topic of another study in our lab which is currently in progress. We have reported and discussed the left and right LTC G1 comparison as follows:

[Results, p. 5]
… In addition, evaluating the left and right hemispheres separately, we observed high similarity of hemisphere-specific LTC G1 axes ($r = 0.74$, $p_{variogram} < 0.001$; Fig. S5).

[Supplements, p. 6]

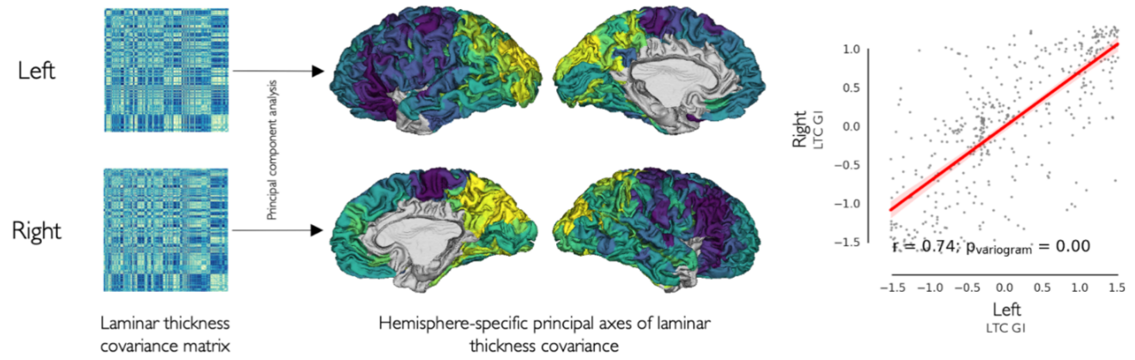

Fig. S5. Hemisphere-specific axes of LTC. LTC and its principal axis was calculated separately on the left and right hemispheres. The principal axes of left and right hemispheres were significantly correlated.

[Discussion, p. 16]
In this study, we used the whole-brain map of cortical layers from a single individual, the BigBrain [9,42]. This is currently the only whole-brain and high-resolution map of cortical layers available, and until a similar atlas becomes available, it is unclear how much our findings would generalize to the other individuals. Of note, when we compared left and right hemispheres of the same individual, we observed similar principal axes, which hints at intra-individual inter-hemispheric consistency of the principal axis of laminar thickness covariance. …

4) Some statements are too sweeping; for example, bottom of p.8 "varying density of PV+ and CB+ inhibitory neuronal subtypes along cortical types is reported in macaque cortex." But ref. 23 refers only to prefrontal cortical systems. The statement needs to be better substantiated or further qualified (ex: DeFelipe...Elston, 1999 for macaque early visual areas).
Similarly, page 8 "from V1 to prefrontal cortex there is an increase in the density of dendritic spines." We do have a detailed comparison of monkey PFC and V1 (JI Luebke, 2017; JP Gilman et al., 2017) but that is a comparison only between two areas. The several relevant papers of Guy Elston are suggestive, but are also not comprehensive. In this regard, a relevant recent paper is Benavides-Piccione et al., 2021 https://pubmed.ncbi.nlm.nih.gov/33723567/
Similarly, page 8 " we observed that LTC G1 and the maps of cortical hierarchy were co-aligned with regional variability of several subtypes...." This is based on comparisons with the literature. Given the complexity of the putative results and the likely problems of extrapolations, this may be too sweeping.

We thank the reviewer for their detailed suggestions. As mentioned above, this paragraph has been removed from the revised manuscript.

Response to Reviewer # 2

This work was based on the BigBrain histological atlas of a postmortem human brain. The authors first eliminated a-/dysgranular regions and to avoid the local effect of cortical curvature, they smoothed the laminar thickness using a moving disk. The normalized laminar thickness map was parcellated and the laminar thickness covariance (LTC) matrix was calculated. The LTC was generated by pairwise partial correlation of relative laminar thickness between cortical locations with Z-transformation, which allowed it to be viewed as a kind of 3D representation of the LTC matrix. PCA was applied to identify the main axes along the regional dissimilarity of laminar thickness. They mainly focused on the first PCA component (LTC G1) to extract the feature of the LTC matrix and showed the following characteristics. First, LTC G1 was compared with asymmetry-based and laminar-based hierarchies, which were calculated as the group-averaged effective connective matrix and regional laminar-based hierarchy map of macaques, showing the correlation of r =0.39 and 0.54, respectively. Second, they compared the LTC matrix with the structural connectivity (SC) and functional connectivity (FC) matrices averaged over a subset of the HCP data. SC likelihood and FC strength correlatedly increased with LTC, whereas they decreased with geodesic distance (GD). The SC matrix based on cortical thickness correlated with LTC, and the principal axis of SC correlated (r=0.55) with LTC G1. Third, the maturational coupling matrix was weakly correlated with the LTC matrix. They identified distinct sets of genes expressed at opposite ends of the LTC G1. That is: Rostral and caudal gene expressions are associated with mid-fetal/post-pubertal and fetal/pre-pubertal stages of human development, respectively.

Based on these analyses, the authors conclude the following in the Abstract and the first paragraph of the Discussion, which states that

1) They identified "a spatial pattern of changes in laminar thickness covariance from lateral frontal to posterior occipital regions, which differentiated the dominance of infra-versus supragranular layer thickness".

2) The infragranular-dominant pattern of laminar thickness was associated with higher hierarchical positions of regions, MRI based on resting-state effective connectivity in humans and tract- tracing based structural connections in macaques.

3) They show that regions with comparable laminar thickness patterns correspond to inter-regional structural covariance, mutational coupling, and transcriptome patterning, indicating developmental relevance.

4) They claim that in sum they characterize the association between organization of laminar thickness and processing hierarchy, anchored in ontology.

I agree with the authors on the first and second conclusions. However, I am less convinced about the third conclusion, particularly the developmental relevance from the actual data presented in this paper. My main concerns with the current form of the paper are with the last conclusion, which I feel they have not provided enough evidence to support it and the citations are not appropriate.

We thank the Reviewer for the thorough evaluation and summary of the main points of our paper. Indeed, based on the comments of the Reviewers and editors we have made various alterations in the current work, focusing on the strengths and providing a more balanced perspective on the latter two conclusions and interpretations. Specifically, we agree that the conclusions we can draw from our analyses on the association of LTC with structural covariance, maturational coupling, and gene expression trajectories are limited, and as such, we have presented our interpretations of these findings as speculations. Further, we have decreased the focus of our manuscript on these findings, in favor of an increased focus on elaboration of our structural findings. Please refer to our response to a similar comment from Reviewer #1 (pages 7-10 of this response letter) for the details of the changes made.

The following are issues related to my concerns in the text.

Page 2 (Abstract), lines 25-29: The fact that certain transcriptomic patterns coincide with layer thickness, structural covariance, and mature connectivity does not directly support an ontogenetic link between the organization of layer thickness and processing hierarchies.

We agree with the Reviewer that a direct ontogenic link cannot be drawn based on our findings. We have removed the statement of "ontogenic link" from the text, and as mentioned above, have considerably revised our wording to provide a more balanced interpretation of our findings.

> *[Abstract, p. 2]*
> The human isocortex consists of tangentially organized layers with unique cytoarchitectural properties. These layers show spatial variations in thickness and cytoarchitecture across the neocortex, which is thought to support function through enabling targeted corticocortical connections. Here, leveraging maps of the six cortical layers based on 3D human brain histology, we aimed to quantitatively characterize the systematic covariation of laminar structure in the cortex and its functional consequences. After correcting for the effect of cortical curvature, we identified a spatial pattern of changes in laminar thickness covariance from lateral frontal to posterior occipital regions, which differentiated the dominance of infra- versus supragranular layer thickness. Corresponding to the laminar regularities of cortical connections along cortical hierarchy, the infragranular-dominant pattern of laminar thickness was associated with higher hierarchical positions of regions, mapped based on resting-state effective connectivity in humans and tract-tracing of structural connections in macaques. Moreover, we show that regions with similar laminar thickness patterns have a higher likelihood of structural connections and strength of functional connections. In sum, here we characterize the organization of laminar thickness in the human isocortex and its association with cortico-cortical connectivity, illustrating how laminar organization may provide a foundational principle of cortical function.

Page 4 (Introduction), lines 78-80: The references cited are only partially relevant to the argument. Ref. 20 excludes the involvement of cortical thickness in the relationship to the connectivity property. Ref. 23 emphasizes more the a-/dysgranular layer in relation to PV vs. CB/CR within the PFC. Ref. 25 is an adult neurogenesis perspective and less relevant to the topic.

We had originally cited Refs. 20 and 23 (Dombrowski et al., 2001; Hilgetag, Medalla, Beul, & Barbas, 2016) as they had put forward the hypothesis of developmental relevance of laminar

structure variation, rather than referring to their findings, as rightfully summarized by the Reviewer. We have made this clear in our revised manuscript.

> *[Discussion, p. 16]*
> … The hypothesis of different developmental trajectories relating to gradation of laminar structure has been previously put forward by Hilgetag, Barbas and colleagues [17,22,54]. …

In addition, we agree that instead of Ref. 25 (Pasko Rakic, 2002), a perspective paper, it would be more appropriate to cite the original research summarized in that paper, i.e., (P. Rakic, 1988), and other relevant papers suggested below by the Reviewer.

Page 4 line 82: Again, Ref. 25 is not relevant. Instead, See Rakic (1988), Science 241, 170-176 and Nadarajah, B. and Parnavelas, J.G. (2002) Nat. Rev. Neurosci. 3, 423-432, would be more relevant.

Page 4 lines 82-83. See Dehay, C. and Kennedy, H. (2007) Nat. Rev. Neurosci. 8, 438-450 and their original papers including the extended outer subventricular zone in non-human primates and in humans, e.g. by Hansen et al. Nature (2010) 464, 554-561, which can be cited.

We appreciate the suggestions and have cited them in the revised Discussion. However, with the reduced focus on developmental ideas, we did not summarize the details of suggested studies.

> *[Discussion, p. 16]*
> … There are regional differences in neurogenesis timing and cell-cycle duration throughout fetal development [108–114], or region- and layer-specific neuronal death in early postnatal stages [115] which may result in the specification of regions and their cytoarchitectural variability. …

Page 8 lines 209-211: They say "This suggests shared developmental/maturational and genetic (heritable) and environmental components". However, I see little "developmental", especially prenatal, evidence in the figure in Fig. 4a.

We agree with the Reviewer and have updated this sentence accordingly:

> *[Results, p. 11]*
> Structural covariance matrix reflects the pattern of covariation in cortical morphology (e.g., cortical thickness) across a population, which provides a model of shared maturational and genetic effects between cortical regions [45–47]. We obtained the structural covariance matrix based on the HCP dataset (N = 1113) from our previous work [47], and observed that it was significantly correlated with the LTC at the level of matrices ($r = 0.33$, $p_{spin} < 0.001$) and their principal axes ($r = -0.57$, $p_{variogram} < 0.001$). This may indicate shared maturational and genetic effects between regions with similar laminar thickness (Fig. S17a). …

Page 8, line 225-Page 9, line 229: This is an interesting observation in itself. However, the existence of these two sets of genes adds little to what has already been reported in the field of developmental neurobiology.

We appreciate the feedback and agree with the Reviewer. As mentioned above, in the revision we have limited the conclusion we drew from this finding and have provided a more balanced perspective.

Page 10, line 291- Page 11, line 293: This is too schematic a view. Please also refer to recent studies, e.g. Gao et al, Nature Neurosci., 27, 515-529, (2022); D'Souza et al, Nature Commun, (2022)13:503, and others as well. The authors' own discussion of these recent results is then required.

Indeed, our description of laminar patterns of FF and FB projections has overlooked the more detailed patterns observed in the suggested references. In our revised discussion we have elaborated more on the additional patterns of FF and FB projections:

> *[Discussion, p. 14]*
> … FF connections originate from the supragranular layers II and III and target layer IV of a higher-order region, which are in turn reciprocated by FB connections originating from infragranular layers V and VI and terminating outside layer IV of a lower-order region [28,29,36,38,41]. Of note, more detailed accounts of neuronal projections have revealed additional patterns of FF and FB connections [28,29,37,39,85], such as a FB projections originating from layer II and FF projections originating from layers V and VI [28,29], or FF and lateral projections targeting layer I [37]. …

Page 11 line 310-311: It is not clear how the reference relates to the current study, since the difference between PV and CR in ref 23 is most different between a-/dysgranular and granular cells. However, in this paper, the authors exclude the former group from the analysis.

As Reviewer correctly pointed out, the mentioned study (Dombrowski et al., 2001) reports a-/dysgranular regions as anchors of the regional variability in the inhibitory cell types, and yet, we had to exclude them for methodological considerations. We have nevertheless limited our discussion on the cell types and removed the paragraph in question, also in response to feedback from Reviewer #1 (see page 7 of this letter).

Minor comments
Page 5, line 122 (Fig. 1e): I am not sure what the difference is between the left and right pair of figures. Also, please clarify the relationship to Figs. S2 and S4.

In Fig. 1e the left and right pairs show the left and right hemispheres. To make it clear we have added L and R legends to this and all other figures. In Figs. S2 and S4 we are only showing the left hemisphere to make the figure less crowded, as we are showing multiple brains. In the figures that only show the left hemisphere we have indicated it in the figure caption.

Page 6, line 138 (Fig. S8): In Fig. S8a, "microstructural profile" should read "cortical depth" as shown in Ref. 10.

Here, by "microstructural profile" we refer to the image intensity sampled across cortical depth, rather than the cortical depth itself. The reference mentioned (Paquola et al., 2019), also uses the term "microstructural profile" or "intensity profile" in a similar way.

Response to the Academic Editor

I have had a quick look at the text and there are some problems in the discussion:

Ref 59 Bastos et al., 2012 is a theoretical review of predictive coding and is incorrectly cited on p10. In the same line Felleman and Van Essen 1991 and Rockland and Pandya 1979 are cited for laminar patterns of feedforward (FF) and feedback (FB) connections. These studies are relatively dated and should be completed by Barone..Kennedy 2000, and Markov et al., JCN 2014. The following 2 sentences are confusing, because they both imply that FB are limited to infragranular layers. That is not the case: throughout the extrastriate cortex of NHP there is a robust supragranular FB pathway! This is reported in Markov et al. JCN 2014 and discussed in Vezoli et al., NeuroImage 2021.

We thank the Academic Editor for their suggestions which we have cited as suggested. In addition, we have mentioned the supragranular FB pathway in addition to the FF and FB projection patterns previously described.

> *[Discussion, p. 14]*
> … FF connections originate from the supragranular layers II and III and target layer IV of a higher-order region, which are in turn reciprocated by FB connections originating from infragranular layers V and VI and terminating outside layer IV of a lower-order region [28,29,36,38,41]. Of note, more detailed accounts of neuronal projections have revealed additional patterns of FF and FB connections [28,29,37,39,85], such as a FB projections originating from layer II and FF projections originating from layers V and VI [28,29], or FF and lateral projections targeting layer I [37]. …

I found the whole paragraph at the top of P11 very confused and extremely biased in its citation of the literature. The authors citation 64 for functional is very strange , Michalareas et al., Neuron 2016 is a cortical hierarchy in human based on function which is much more to the point and relevant than ref 64. Finally the conclusion of the penultimate line of this paragraph is simply undefendable "that is, they influence the activity of lower regions rather than being influenced by them refs 65-67)" . This is a speculation based on nothing tangible, and should be removed.

We agree that the suggested reference (Michalareas et al., 2016) is more relevant to the definition of laminar-based hierarchy that we used here. Originally, we had cited Ref. 64 (Margulies et al., 2016) as an example of the many different definitions of 'hierarchy' used in the literature. In the revised manuscript we have discussed the issue of different definitions of 'hierarchy' in the Introduction:

> *[Introduction, p. 4]*
> … Notably, in neuroscience the term 'hierarchy' has been used to describe different phenomena [31], such as gradients of structural and functional features [27,32], topological sequence of connections [33], asymmetry of directional connections indicating inter-regional control or dominance [34,35], or as described above, the sorting of laminar projection patterns and their physiological correlates [28,29,36–41]. Throughout this paper we will focus on the latter two definitions of hierarchy, that is, laminar-based and asymmetry-based hierarchy.

In the discussion, we have discussed the association of feedback and feedforward processing with alpha/beta and gamma activity, citing the relevant and interesting work by Michalareas and colleagues (Michalareas et al., 2016):

> *[Discussion, p. 14]*
> … Interestingly, the FF and FB connections are respectively associated with gamma and alpha/beta rhythms [29,30,40,86–88], which in turn show regional and laminar specificity, with more prominent gamma rhythms in early visual areas and superficial layers and beta rhythms in fronto-parietal areas and infragranular layers [29]. In fact, the asymmetry of FF and FB projections inferred based on magnetoencephalography has been previously used to map the cortical hierarchy of visual areas in humans [40]. ….

We also agree with the Academic Editor's opinion about the sentence "that is, they influence the activity of lower regions rather than being influenced by them", and have removed it.

The last line ;" The hierarchy in macaque was defined based on the laminar patters of feedforward and feedback connections identified in tract-tracing studies ref 39. The tract tracing studies in question were done by the Kennedy lab and the resulting hierarchy published in Markov JCN 2014 and then subsequently in more detail in Vezoli NeuroImage 2021. BTW the Vezoli et al 2021 is highly pertinent to the present study and should be cited.

We appreciate the suggestions and agree these are more appropriate citations. We had originally cited (Markov, Ercsey-Ravasz, et al., 2014) and (Markov, Vezoli, et al., 2014) elsewhere in our Discussion and Methods. However, in the revision we have cited these articles as well as (Vezoli et al., 2021) to refer to "tract-tracing studies".

The paragraph beginning line 319 makes much of the strength of connections and its consequence for understanding large-scale models of the cortex. The authors cite numerous papers looking at the issue uniquely from the perspective of the structural model and go out of the way to cite the alternative which is based on connectivity Ercsey-Ravasz et al., Neuron 2013, Markov et al., Science 2013. These studies from the Kennedy lab have shown that connectivity based models have strong predictability and again should be cited.

The distance rule model of connectivity had been originally mentioned in our Discussion as an alternative to the structural model in explaining likelihood of connections. We have elaborated more on the distance rule in our discussion by citing the suggested references and discussing its importance in the laminar pattern of connections as well.

> *[Discussion, p. 14]*
> … The laminar pattern of corticocortical connections is suggested to relate to the gradation of cortical microstructure (the 'structural model') [3,17,25,26] or the physical proximity of regions (the 'distance rule model') [28,29]. These models suggest that the laminar connections of cytoarchitecturally similar or proximal regions are mostly lateral, but the pattern of connections become increasingly FF/FB as regions are more dissimilar in cytoarchitecture or are more distant [3,17,25,28,29,83]. …
>
> *[Discussion, p. 15]*
> … An alternative model of connectivity is the 'distance rule model' which proposes physical proximity as the main predictor of connectivity as a result of wiring cost minimization

[61,62,104–106]. It can be argued that the increased connectivity of similar regions may be an epiphenomenon of the distance rule, as nearby cortical regions tend to be similar [63,64]. However, it has been shown that the distance rule alone does not fully account for the connectome architecture. For example, simulated connectomes were shown to better resemble the empirical connectomes when inter-regional similarity was considered in addition to the wiring cost reduction [93]. Recent studies on tract-tracing data have shown that both similarity of cortical types and physical proximity can predict likelihood of structural connections [25,83], though in most species cytoarchitectonic similarity was related to connectivity, above and beyond physical proximity [83]. Nevertheless, in our study long-range connections were not significantly associated with similarity of laminar thickness profiles, suggesting distance as an important covariate. …

Finally, in the paragraph starting line 352 there is a long-winded attempt to relate adult structure to developmental processes. Here again the Kennedy lab has carried out numerous studies on showing the role of cortical neurogenesis on adult cytoarchitecture in rodents and NHP, this body of work including reviews and original studies none of which are cited. Instead they continue to cite their colleague Hilgetag (refs 23, 26 , 30) co-adapts of the structural model.

We agree with the Academic Editor that our findings on association of LTC with structural covariance, maturational coupling and developmental trajectory of gene expression can only indirectly relate adult laminar structure to developmental processes. As mentioned above, in response to similar comments from Reviewers, we have revised the manuscript to present these findings with a more balanced interpretation, while making our speculations clear. Further, we have focused more on the structural findings and therefore reduced our discussion of the developmental hypothesis of laminar structure. Nevertheless, we believe the suggested references by the Academic Editor to be very relevant for the interpretation of the current work, and have mentioned them in our Discussion.

[Discussion, p. 16]
We observed higher inter-regional LTC was linked to higher population-level inter-regional structural covariance, which potentially indicates shared genetic and maturational effects among regions [65,67]. In addition, we observed a significant but weak correlation of LTC with subject-level longitudinal maturational coupling of cortical regions during childhood and adolescence [66], and found distinct pre- and postnatal developmental trajectories of genes overexpressed at the two ends of LTC G1. We speculate that regional differences in gene expression and developmental trajectories may underlie regional variability of laminar structure. The hypothesis of different developmental trajectories relating to gradation of laminar structure has been previously put forward by Hilgetag, Barbas and colleagues [17,22,54]. There are regional differences in neurogenesis timing and cell-cycle duration throughout fetal development [108–114], or region- and layer-specific neuronal death in early postnatal stages [115] which may result in the specification of regions and their cytoarchitectural variability. However, our current findings can only indirectly suggest developmental relevance of laminar thickness organization. Further studies are needed to investigate this hypothesis by using postmortem histology or in-vivo markers of laminar structure [10,116,117] during pre- and postnatal development to characterize maturation of laminar structure at different stages of development and better understand the potential developmental mechanisms underlying regional variations in laminar structure.

In light of the above I think there is a clear need for the authors to re-write the discussion and give a much more balanced view of the field.

We appreciate the Academic Editor for their helpful feedback and the suggested relevant references. As mentioned above, we have rewritten important parts of the discussion and aimed to refer to a more diverse selection of citations.

References

Charvet, C. J., Cahalane, D. J., & Finlay, B. L. (2015). Systematic, Cross-Cortex Variation in Neuron Numbers in Rodents and Primates. *Cerebral Cortex*, *25*(1), 147–160. doi: 10.1093/cercor/bht214

Dombrowski, S. M., Hilgetag, C. C., & Barbas, H. (2001). Quantitative architecture distinguishes prefrontal cortical systems in the rhesus monkey. *Cerebral Cortex (New York, N.Y.: 1991)*, *11*(10), 975–988. doi: 10.1093/cercor/11.10.975

García-Cabezas, M. Á., Hacker, J. L., & Zikopoulos, B. (2020). A Protocol for Cortical Type Analysis of the Human Neocortex Applied on Histological Samples, the Atlas of Von Economo and Koskinas, and Magnetic Resonance Imaging. *Frontiers in Neuroanatomy*, *14*. doi: 10.3389/fnana.2020.576015

García-Cabezas, M. Á., Hacker, J. L., & Zikopoulos, B. (2022). Homology of neocortical areas in rats and primates based on cortical type analysis: An update of the Hypothesis on the Dual Origin of the Neocortex. *Brain Structure and Function*. doi: 10.1007/s00429-022-02548-0

Garin, C. M., Hori, Y., Everling, S., Whitlow, C. T., Calabro, F. J., Luna, B., … Constantinidis, C. (2022). An evolutionary gap in primate default mode network organization. *Cell Reports*, *39*(2). doi: 10.1016/j.celrep.2022.110669

Goulas, A., Changeux, J.-P., Wagstyl, K., Amunts, K., Palomero-Gallagher, N., & Hilgetag, C. C. (2021). The natural axis of transmitter receptor distribution in the human cerebral cortex. *Proceedings of the National Academy of Sciences of the United States of America*, *118*(3). doi: 10.1073/pnas.2020574118

Goulas, A., Uylings, H. B. M., & Stiers, P. (2014). Mapping the hierarchical layout of the structural network of the macaque prefrontal cortex. *Cerebral Cortex (New York, N.Y.: 1991)*, *24*(5), 1178–1194. doi: 10.1093/cercor/bhs399

Goulas, A., Zilles, K., & Hilgetag, C. C. (2018). Cortical Gradients and Laminar Projections in Mammals. *Trends in Neurosciences*, *41*(11), 775–788. doi: 10.1016/j.tins.2018.06.003

Hilgetag, C. C., & Goulas, A. (2020). 'Hierarchy' in the organization of brain networks. *Philosophical Transactions of the Royal Society B: Biological Sciences*, *375*(1796), 20190319. doi: 10.1098/rstb.2019.0319

Hilgetag, C. C., Medalla, M., Beul, S. F., & Barbas, H. (2016). The primate connectome in context: Principles of connections of the cortical visual system. *NeuroImage*, *134*, 685–702. doi: 10.1016/j.neuroimage.2016.04.017

Huber, L., Finn, E. S., Chai, Y., Goebel, R., Stirnberg, R., Stöcker, T., … Poser, B. A. (2020). Layer-dependent functional connectivity methods. *Progress in Neurobiology*, 101835. doi: 10.1016/j.pneurobio.2020.101835

Margulies, D. S., Ghosh, S. S., Goulas, A., Falkiewicz, M., Huntenburg, J. M., Langs, G., … Smallwood, J. (2016). Situating the default-mode network along a principal gradient of macroscale cortical organization. *Proceedings of the National Academy of Sciences*, *113*(44), 12574–12579. doi: 10.1073/pnas.1608282113

Markov, N. T., Ercsey-Ravasz, M. M., Ribeiro Gomes, A. R., Lamy, C., Magrou, L., Vezoli, J., … Kennedy, H. (2014). A weighted and directed interareal connectivity matrix for macaque cerebral cortex. *Cerebral Cortex (New York, N.Y.: 1991)*, *24*(1), 17–36. doi: 10.1093/cercor/bhs270

Markov, N. T., Vezoli, J., Chameau, P., Falchier, A., Quilodran, R., Huissoud, C., … Kennedy, H. (2014). Anatomy of hierarchy: Feedforward and feedback pathways in macaque visual cortex. *Journal of Comparative Neurology*, *522*(1), 225–259. doi: 10.1002/cne.23458

Michalareas, G., Vezoli, J., van Pelt, S., Schoffelen, J.-M., Kennedy, H., & Fries, P. (2016). Alpha-Beta and Gamma Rhythms Subserve Feedback and Feedforward Influences among Human Visual Cortical Areas. *Neuron*, *89*(2), 384–397. doi: 10.1016/j.neuron.2015.12.018

Paquola, C., De Wael, R. V., Wagstyl, K., Bethlehem, R. A., Hong, S.-J., Seidlitz, J., … Margulies, D. S. (2019). Microstructural and functional gradients are increasingly dissociated in transmodal cortices. *PLOS Biology*, *17*(5), e3000284.

Rakic, P. (1988). Specification of cerebral cortical areas. *Science (New York, N.Y.)*, *241*(4862), 170–176. doi: 10.1126/science.3291116

Rakic, Pasko. (2002). Neurogenesis in adult primate neocortex: An evaluation of the evidence. *Nature Reviews. Neuroscience*, *3*(1), 65–71. doi: 10.1038/nrn700

Vezoli, J., Magrou, L., Goebel, R., Wang, X.-J., Knoblauch, K., Vinck, M., & Kennedy, H. (2021). Cortical hierarchy, dual counterstream architecture and the importance of top-down generative networks. *NeuroImage*, *225*, 117479. doi: 10.1016/j.neuroimage.2020.117479

Wagstyl, K., Larocque, S., Cucurull, G., Lepage, C., Cohen, J. P., Bludau, S., … Evans, A. C. (2020). BigBrain 3D atlas of cortical layers: Cortical and laminar thickness gradients diverge in sensory and motor cortices. *PLOS Biology*, *18*(4), e3000678. doi: 10.1371/journal.pbio.3000678

Xu, T., Nenning, K.-H., Schwartz, E., Hong, S.-J., Vogelstein, J. T., Goulas, A., … Langs, G. (2020). Cross-species functional alignment reveals evolutionary hierarchy within the connectome. *NeuroImage*, *223*, 117346. doi: 10.1016/j.neuroimage.2020.117346

Zilles, K., & Palomero-Gallagher, N. (2017). Multiple Transmitter Receptors in Regions and Layers of the Human Cerebral Cortex. *Frontiers in Neuroanatomy*, *11*, 78. doi: 10.3389/fnana.2017.00078
