# Supplementary material for: The regional variation of laminar thickness in the human isocortex is related to cortical hierarchy and interregional connectivity
Source: PLoS Biol. 2023 Nov 9;21(11):e3002365. doi: 10.1371/journal.pbio.3002365 (PMC10684102; doi:10.1371/journal.pbio.3002365)
Supplement: Attachment — Submitted filename: Response Letter.pdf [file pbio.3002365.s020.pdf]

Response letter

***PBIOLOGY-D-23-00811R2**, The spatial arrangement of laminar thickness profiles in the human isocortex scaffolds cortical organization*

We thank the reviewers and editors for their helpful re-evaluation of our revised manuscript and the opportunity to submit a second revised manuscript. We have detailed our responses below as well as the changes made in the manuscript. We hope to have addressed the raised concerns and look forward to the feedback on the current version of the manuscript.

Reviewer #1

The Authors appear to have made extensive revisions, and include detailed point-by-point responses to previous comments. On closer reading and comparison of R2 and R1, however, I found the revisions to be less extensive than at first glance, and ended up with many of the same misgivings that I had with the original manuscript.

We appreciate the Reviewer's feedback on R2 and hope to have addressed their concerns in the current revised manuscript.

A main concern, echoing Reviewer 2, is the attempt to correlate findings on laminar thickness with development (dialogue, page 7, 8). 1) The developmental epochs given (bottom of Response page 8) are broad: "mid-fetal and post-pubertal," "late fetal and pre-pubertal." There is a huge literature on developmental events and timetables, as the Authors will know. Obviously, further detail would detract even more from the primary focus of the present paper and might be best addressed in a separate publication. However, it can seem superficial not to incorporate (compare, not only just cite) the many relevant results of established researchers in that field such as Kostovic, Molnar, and Huttner among others. My opinion remains that the sections on development do not reinforce, but rather detract from the main findings and conclusions. Similar comment about reworded paragraph in the Discussion (page 10 of Response). The results can stand alone, and are better focused without the developmental distraction.

We thank the Reviewer for this comment and agree that the developmental relevance of laminar structure is an important and intriguing question that can be investigated in depth in another dedicated paper. Our treatment of this matter in the current paper is an initial exploratory investigation which can be continued in further in-depth studies in the future. We believe, in agreement with the Academic Editor, that reporting our limited and brief findings on the developmental ideas is useful, as it reinforces the question of *how* the (adult) laminar structure variations come about, beyond the questions of *what* and *why*, which were the main foci of the current paper.

Having said that, we agree that further elaboration and discussion of these findings in the current paper could detract from our primary objectives. Therefore, in the revised manuscript we have shortened this Results section in the main text, by moving the transcriptomics analyses and their methods to Text S1.

Moreover, we appreciate the Reviewer's suggestion to compare our developmental transcriptomics findings with the research on the genetic regulation of cortical lamination and arealization. However, in the current work the transcriptomic analysis was rather a spatial decoding approach, to contextualize main findings of the work and explore relationships with transcriptomic expression across the lifespan, rather than a second step in the form of a in depth evaluation of gene regulation of laminar maturation, which would warrant a whole new study. We believe that given the different nature and focus of such investigations, versus our approach, a head-to-head comparison of the results is not readily feasible within the framework of the current study. At the same time, we agree that these works are relevant and should be mentioned in our discussion, yet, to avoid further expanding this section and risk distracting the reader, we refer the reader to the relevant publications for more detail.

In addition, we have improved our Discussion of the developmental ideas by additionally elaborating on the relevance of OSVZ zone expansion to externopyramidization, as suggested by the Reivewer #2 (see page 6 of this letter).

Please see our updated Discussion on the developmental ideas below. Please note that as Text S1 is available as a separate file and is five pages, we will not repeat its content in the response letter.

> *[Discussion, p. 13]*
> Having studied the what and why questions of laminar thickness covariance, we also explored the question of how the (adult) laminar structure variations may come about. A central hypothesis on the origins of laminar structure variability proposes that different developmental trajectories across regions may relate to the gradation of laminar structure [17,22,103]. There are regional differences in neurogenesis timing and cell-cycle duration throughout fetal development [104–110], or region- and layer-specific neuronal death in early postnatal stages [111] which may result in the specification of regions and their cytoarchitectural variability. For example, outer subventricular zone, a germinal zone of the developing cortex that is thought to generate the expanded primate granular and supragranular layers, is denser and deeper in area 17 compared to area 18 and has an increased rate of cell cycles, leading to a marked expansion of the upper layers in this region [104,105,109,112]. In the current study, we observed higher inter-regional LTC was linked to higher population-level inter-regional structural covariance, which potentially indicates shared genetic and maturational effects among regions [60,62]. In addition, we observed a significant but weak correlation of LTC with subject-level longitudinal maturational coupling of cortical regions during childhood and adolescence [61], and found distinct pre- and postnatal developmental trajectories of genes overexpressed at the two ends of LTC G1 (Text S1). Importantly, our current findings only indirectly suggest developmental relevance of laminar thickness organization. For example, the transcriptomics analysis involves mere spatial colocalization of the LTC G1 with the gene expression maps and the developmental enrichment of those genes, and therefore, lacks mechanistic insights on the complex gene regulatory mechanisms underlying regional differences of laminar structure. We refer the interested reader to the rich literature on cortical arealization and its genetic regulation [113–116]. Consequently, further research will be needed to study the developmental relevance of laminar structure variability by investigating postmortem histology or in-vivo markers of laminar structure [10,117,118] at different stages of development to shed light on the maturation of laminar structure and its regional variability.

I might make the same comment about the link to macaque connectivity, which also can appear superficial and has in any case been previously emphasized by this Group.

There are indeed shortcomings with our cross-species comparison of the laminar thickness data from humans with the macaque connectivity, as is pointed out in our Discussion. However, the laminar connectivity data can only be obtained from macaques based on the invasive tract-tracing approach, which is not available in humans. In addition, we understand that our analysis of the macaque connectivity may be minimal. A detailed accounts of the macaque connectivity data has not been the focus of our study, and such investigations can be found in multiple papers dedicated to this topic including the works of Kennedy and Hilgetag laboratories. Rather, here we have obtained the laminar-based hierarchy map from another publication (Burt et al., 2018). Our aim was to have an alternative definition of

hierarchy based on laminar connections, which is more directly related to our investigations on laminar anatomy, as compared to the asymmetry-based hierarchy obtained based on human resting-state fMRI data. We believe that both measures combined will better underscore the relevance of cortical hierarchy to the laminar thickness covariance and overcomes each method's shortcomings.

The use of feedback/feedforward beyond the early sensory pathways remains problematic (why discard Maunsell and Felleman "lateral?"). The Author Response, (pages 19, and 20 in reference to Discussion page 14) seems minimal - just to note (in passing?) the existence of "additional patterns." This is arguably disingenuous, and not a clear or useful correction. Key papers in visual cortex are still missing: A. Angelucci and colleagues (2006, 2020, 2021 among others); and S.Shen...Tolias (2022) on "...two cortico-cortical feedback pathways." There is also a 2022 Frontiers Research Topic "Feed-forward and Feedback Processing...." (comments by E. Zagha; K. Rockland; among others). Likely, there is not space, in a Short Report, to adequately discuss the relevant points, and the best option may be to curtail or postpone discussion here, or refer to previous relevant pubs where this is already raised by the present Authors.

We thank the Reviewer for these suggestions. We made the following changes to improve our description of the laminar projections:

- Based on (Rockland, 2022) we mentioned the fact that feedback connections can be reciprocal to feed-forward connections, while not always being the case.

> *[Discussion, p. 11]*
> … FF connections originate from the supragranular layers II and III and target layer IV of a higher-order region, whereas FB connections originate from infragranular layers V and VI and terminate outside layer IV of a lower-order region [28,29,36,38,41], which may reciprocate FF connections [78]. …

- We also added a brief description on the distinct functions of FB and FF projections, by citing (Zagha, 2020) among others:

> *[Discussion, p. 11]*
> … The FF and FB projections are thought to have distinct physiological roles, that is, FF projections carry high-dimensional (sensory) information up the hierarchy, whereas FB projections propagate context and modulate the function of lower-order regions [29,30,80]. …

- In addition, we included a description of the lateral connections:

> *[Discussion, p. 11]*
> … In addition, lateral connections originate from supra- and infragranular layers and terminate across all the layers, connecting regions at a similar level [38]. …

As the Reviewer suggested, there are many more interesting details within the vast literature on the anatomy and function of laminar projections, such as the papers of Angelucci et al. on the function of FF and FB projections within the visual system, and Shen et al. on the two FB pathways. Yet, we also agree that within the limited extent of our discussion, we cannot provide a comprehensive review of this literature without detracting from our main aims and keeping the discussed points relevant to our findings.

In summary, this could be an interesting and focused addition to the several papers from this Group on cortical laminar thickness. The correlations with development and macaque connectivity detract from the core findings, especially in what is a Short Report, and obscure what can be an interesting approach to deal with complex issues of lamination and area organization.

We thank the Reviewer for the helpful feedback! We hope that the alterations made can further contribute underscoring the strength of the work.

If there is space, "limitations" section (as in Wagstyl et al., 2020) would be useful, as perhaps also a brief section on "future directions."

As suggested, we have included a "Limitations and future directions" section in the Discussion, discussing the issues of BigBrain being a single subject, as well as the issues related to the pre-defined number of layers, which were previously discussed elsewhere in the text.

> *[Discussion, p. 13]*
> **Limitations and future directions**
> In this study, we used the whole-brain map of cortical layers from a single individual, the BigBrain [9,42]. This is currently the only whole-brain and high-resolution map of cortical layers available, and until a similar atlas becomes available, it is unclear how much our findings would generalize to the other individuals. Of note, when we compared left and right hemispheres of the same individual, we observed similar principal axes, which hints at intra-individual inter-hemispheric consistency of the principal axis of laminar thickness covariance. In addition to generalizability, an intriguing question for the future research is the degree to which laminar structure varies across individuals, and how it might relate to behavior and function, and its changes through development. This highlights the importance of future studies on in-vivo estimation of laminar structure based on high-resolution imaging.
>
> We studied laminar thickness covariance using a six-layer model of the isocortex, previously created using a convolutional neural network [42]. However, it is well known that some isocortical areas have fewer or a greater number of layers, due to the individual layers being absent, or being divided into sublayers [1,4,119]. For example, area V1 is characterized by a prominent layer IV that is divided into three sublayers, and on the other hand, layer IV is unclear in agranular regions [4,14,119]. To avoid forcing a six-layer model in regions with fewer number of layers and less clear layer boundaries, we excluded a- and dysgranular regions from our analyses. Exclusion of these regions limits the generalizability of our findings to the whole extent of the isocortex, yet we showed that the LTC G1 map was consistent when these regions are included. In addition, to further explore the impact of a priori defined number of layers, we used a three-layer model of supragranular, granular and infragranular layers, and observed a similar principal axis. This indicates that LTC G1 captures variations of thickness in the supragranular, granular and infragranular layer groups rather than the individual layers within each group. Future research may account for the regional differences in the number of layers using more fine-grained models of intra-cortical structure where the number of layers in each location is determined based on the data rather than being fixed. This would enable formally testing the optimal architecture of cortical depth and enables inclusion of a-/dysgranular areas in a more comprehensive model of laminar structure in the cerebral cortex.

Reviewer #2

The authors have largely addressed my concerns. I have only a few comments to add.

We thank the Reviewer for the helpful suggestion and have addressed their further comments in a point-by-point fashion below.

1. In the second paragraph of the Discussion, the authors cite references 69, 70, 71, 72 published by Barbara Finlay's laboratory. The series of studies by Finlay focus on layers II-VI, and layers V-VI as a separate set for cross-validation of rostral and caudal cortical gradients across mammalian species. The Materials and Methods section of Reference 69 clearly states, "Measurements of layers were taken orthogonal to the cortical surface area. In our samples, it was difficult to reliably distinguish layers II and III. Moreover, layer IV could not be reliably visualized in some parts of the isocortex (e.g., agranular cortex). In order to systematically compare neuron numbers in various layers across the RC and ML axes of the isocortex, we combined layers II-III and IV in our analyses. Layer V and VI were analyzed together. We therefore only examine how supragranular (layer II-IV), infragranular (layers V-VI) neurons, and their sum vary across the RC and ML axes of the isocortex". Thus, they did not distinguish layer IV from layers II and III. In contrast, in the current study, layer IV is the key layer that characterizes the BigBrain separately from the supragranular layers of layers II and III. In the immediately following sentence the authors state: "In an integrated model of combined laminar thickness and intensity variations, we observed a similar rostral to caudal principal axis characterizing increased gray matter density in all layers, most prominently in layer IV". I think this is not a logical smooth flow.

We appreciate the Reviewer's feedback. We apologize for the unclear sentence structure. Accordingly, we have now reformalized the text to make the description of their work and its relevance to our work clearer:

> *[Discussion, p. 9]*
> … This was in line with a previous animal study which illustrated relative increase in the implied column height of the upper layers along the rostro-caudal axis of the cerebral cortex in several rodent and non-human primate species [63]. The same study also reported that from rostral to caudal regions the density of neurons increases, as had been shown in a few other studies [64–66], but additionally reported the increase to be more prominent in layers II-IV rather than layers V-VI (without differentiating the individual layers in each layer group). In an integrated model of combined laminar thickness and intensity variations we observed a rostral to caudal principal axis, similar to LTC G1, characterizing increased grey-matter density in all the layers, most prominently in layer IV. …

2. Externopyramidization may be related to the expansion of the outer subventricular zone (Dehay & Kennedy, 2007; Smart, I. H. M. et al., Cereb. Cortex 12, 37-53, 2002). The authors cite references 113 and 114, but do not specifically mention it in the discussion.

We thank the reviewer for raising this point and have now incorporated this in our Discussion:

> *[Discussion, p. 13]*

> … There are regional differences in neurogenesis timing and cell-cycle duration throughout fetal development [104–110], or region- and layer-specific neuronal death in early postnatal stages [111] which may result in the specification of regions and their cytoarchitectural variability. For example, outer subventricular zone, a germinal zone of the developing cortex that is thought to generate the expanded primate granular and supragranular layers, is denser and deeper in area 17 compared to area 18 and has an increased rate of cell cycles, leading to a marked expansion of the upper layers in this region [104,105,109,112]. …

3. A minor comment. There are some mixed styles in the figure legends. Fig. 1: small letters for numbering (a, b, c, with bold and non-bold letters), Fig. 2: capital letters for numbering.

Thanks! We've fixed the inconsistency issues in the figure legends.

References

Burt, J. B., Demirtaş, M., Eckner, W. J., Navejar, N. M., Ji, J. L., Martin, W. J., Bernacchia, A., Anticevic, A., & Murray, J. D. (2018). Hierarchy of transcriptomic specialization across human cortex captured by structural neuroimaging topography. *Nature Neuroscience*, *21*(9), Article 9. https://doi.org/10.1038/s41593-018-0195-0

Rockland, K. S. (2022). Notes on Visual Cortical Feedback and Feedforward Connections. *Frontiers in Systems Neuroscience*, *16*. https://www.frontiersin.org/articles/10.3389/fnsys.2022.784310

Zagha, E. (2020). Shaping the Cortical Landscape: Functions and Mechanisms of Top-Down Cortical Feedback Pathways. *Frontiers in Systems Neuroscience*, *14*. https://www.frontiersin.org/articles/10.3389/fnsys.2020.00033
