# Supplementary material for: The regional variation of laminar thickness in the human isocortex is related to cortical hierarchy and interregional connectivity
Source: PLoS Biol. 2023 Nov 9;21(11):e3002365. doi: 10.1371/journal.pbio.3002365 (PMC10684102; doi:10.1371/journal.pbio.3002365)
Supplement: Peer review history

# Editor report: Decision Letter 0

**DOI:** 10.1371/journal.pbio.3002365.r001

**Authors:** Roland G Roberts

7 Apr 2023

Dear Dr Valk, 

Thank you for submitting your manuscript entitled "The spatial arrangement of laminar thickness profiles in the human cortex scaffolds processing hierarchy" for consideration as a Research Article by PLOS Biology.

Your manuscript has now been evaluated by the PLOS Biology editorial staff, as well as by an academic editor with relevant expertise, and I'm writing to let you know that we would like to send your submission out for external peer review.

However, before we can send your manuscript to reviewers, we need you to complete your submission by providing the metadata that is required for full assessment. To this end, please login to Editorial Manager where you will find the paper in the 'Submissions Needing Revisions' folder on your homepage. Please click 'Revise Submission' from the Action Links and complete all additional questions in the submission questionnaire.

Once your full submission is complete, your paper will undergo a series of checks in preparation for peer review. After your manuscript has passed the checks it will be sent out for review. To provide the metadata for your submission, please Login to Editorial Manager (https://www.editorialmanager.com/pbiology) within two working days, i.e. by Apr 11 2023 11:59PM.

If your manuscript has been previously peer-reviewed at another journal, PLOS Biology is willing to work with those reviews in order to avoid re-starting the process. Submission of the previous reviews is entirely optional and our ability to use them effectively will depend on the willingness of the previous journal to confirm the content of the reports and share the reviewer identities. Please note that we reserve the right to invite additional reviewers if we consider that additional/independent reviewers are needed, although we aim to avoid this as far as possible. In our experience, working with previous reviews does save time. 

If you would like us to consider previous reviewer reports, please edit your cover letter to let us know and include the name of the journal where the work was previously considered and the manuscript ID it was given. In addition, please upload a response to the reviews as a 'Prior Peer Review' file type, which should include the reports in full and a point-by-point reply detailing how you have or plan to address the reviewers' concerns. 

During the process of completing your manuscript submission, you will be invited to opt-in to posting your pre-review manuscript as a bioRxiv preprint. Visit http://journals.plos.org/plosbiology/s/preprints for full details. If you consent to posting your current manuscript as a preprint, please upload a single Preprint PDF. 

Feel free to email us at plosbiology@plos.org if you have any queries relating to your submission.

Kind regards,

Roli Roberts

Roland Roberts, PhD

Senior Editor

PLOS Biology

rroberts@plos.org

# Peer review: Decision Letter 1

**DOI:** 10.1371/journal.pbio.3002365.r002

**Authors:** Roland G Roberts

15 May 2023

Dear Dr Valk,

Thank you for your patience while your manuscript "The spatial arrangement of laminar thickness profiles in the human cortex scaffolds processing hierarchy" was peer-reviewed at PLOS Biology. It has now been evaluated by the PLOS Biology editors, an Academic Editor with relevant expertise, and by two independent reviewers. 

In light of the reviews, which you will find at the end of this email, we would like to invite you to revise the work to thoroughly address the reviewers' reports.

IMPORTANT - Please note the following:

a) You’ll see that reviewer #1 is broadly positive, but wants considerably more discussion, especially around the different layer models and issues of intra-areal heterogeneity. S/he also suggests that you de-emphasise (or explicitly mark as speculative) correlations with non-structural data (gene expression, etc.), as these are poorly supported. Reviewer #2 is also positive, but raises concerns about the support for one of your claims (this overlaps somewhat with reviewer #1's concerns).

b) Although not mentioned directly by the reviewers in their comments to authors, during the cross-commenting phase, one of the reviewers pointed out that your findings are from a single human individual, i.e. that used by the BigBrain initiative. You state clearly that corresponding data for other individuals are not available, and you mention this fact prominently as a limitation. However, n=1 is not normally adequate for the standard that we uphold for our Research Articles. As a result, we have changed the article type to Short Report; because your paper is already concise, and within the maximum allowed number of Figures (four) for this article type, no re-formatting is required. We hope that you understand our reasons for doing this.

c) When this point was raised during the cross-commenting, one of the reviewers said "An obvious problem... is the n=1. The interpretations and conclusions are simply too strong given the inherent limitation of the data set. Perhaps the Authors might have undertaken a left-right hemisphere comparison of laminar thickness alone…"

d) When I discussed this and other concerns with the Academic Editor, s/he noted some issues regarding the choice of citations in the Discussion. I have included an edited version of Academic Editor's comments at the foot of this email, and you should address these alongside the reviewers' comments.

Given the extent of revision needed, we cannot make a decision about publication until we have seen the revised manuscript and your response to the reviewers' comments. Your revised manuscript is likely to be sent for further evaluation by all or a subset of the reviewers.

We expect to receive your revised manuscript within 3 months. Please email us (plosbiology@plos.org) if you have any questions or concerns, or would like to request an extension. 

At this stage, your manuscript remains formally under active consideration at our journal; please notify us by email if you do not intend to submit a revision so that we may withdraw it.

**IMPORTANT - SUBMITTING YOUR REVISION**

Your revisions should address the specific points made by each reviewer. Please submit the following files along with your revised manuscript:

1. A 'Response to Reviewers' file - this should detail your responses to the editorial requests, present a point-by-point response to all of the reviewers' comments, and indicate the changes made to the manuscript. 

*NOTE: In your point-by-point response to the reviewers, please provide the full context of each review. Do not selectively quote paragraphs or sentences to reply to. The entire set of reviewer comments should be present in full and each specific point should be responded to individually, point by point.

You should also cite any additional relevant literature that has been published since the original submission and mention any additional citations in your response. 

2. In addition to a clean copy of the manuscript, please also upload a 'track-changes' version of your manuscript that specifies the edits made. This should be uploaded as a "Revised Article with Changes Highlighted" file type. 

*Re-submission Checklist*

When you are ready to resubmit your revised manuscript, please refer to this re-submission checklist: https://plos.io/Biology_Checklist

To submit a revised version of your manuscript, please go to https://www.editorialmanager.com/pbiology/ and log in as an Author. Click the link labelled 'Submissions Needing Revision' where you will find your submission record. 

Please make sure to read the following important policies and guidelines while preparing your revision:

*Published Peer Review*

Please note while forming your response, if your article is accepted, you may have the opportunity to make the peer review history publicly available. The record will include editor decision letters (with reviews) and your responses to reviewer comments. If eligible, we will contact you to opt in or out. Please see here for more details:

https://blogs.plos.org/plos/2019/05/plos-journals-now-open-for-published-peer-review/

*PLOS Data Policy*

Please note that as a condition of publication PLOS' data policy (http://journals.plos.org/plosbiology/s/data-availability) requires that you make available all data used to draw the conclusions arrived at in your manuscript. If you have not already done so, you must include any data used in your manuscript either in appropriate repositories, within the body of the manuscript, or as supporting information (N.B. this includes any numerical values that were used to generate graphs, histograms etc.). For an example see here: http://www.plosbiology.org/article/info%3Adoi%2F10.1371%2Fjournal.pbio.1001908#s5

*Blot and Gel Data Policy*

We require the original, uncropped and minimally adjusted images supporting all blot and gel results reported in an article's figures or Supporting Information files. We will require these files before a manuscript can be accepted so please prepare them now, if you have not already uploaded them. Please carefully read our guidelines for how to prepare and upload this data: https://journals.plos.org/plosbiology/s/figures#loc-blot-and-gel-reporting-requirements

*Protocols deposition*

To enhance the reproducibility of your results, we recommend that if applicable you deposit your laboratory protocols in protocols.io, where a protocol can be assigned its own identifier (DOI) such that it can be cited independently in the future. Additionally, PLOS ONE offers an option for publishing peer-reviewed Lab Protocol articles, which describe protocols hosted on protocols.io. Read more information on sharing protocols at https://plos.org/protocols?utm_medium=editorial-email&utm_source=authorletters&utm_campaign=protocols

Thank you again for your submission to our journal. We hope that our editorial process has been constructive thus far, and we welcome your feedback at any time. Please don't hesitate to contact us if you have any questions or comments.

Sincerely,

Roland

Roland Roberts, PhD

Senior Editor

PLOS Biology

rroberts@plos.org

------------------------------------

REVIEWERS' COMMENTS:

Reviewer #1:

The present communication, from an established and expert group of investigators, sets out to quantitatively characterize co-variance of laminar thickness across 6-layered neocortical areas and further attempts to correlate results with ideas of hierarchical organization, genetic expression gradients, and developmental trajectories. I found the experimental results on laminar thickness trends interesting, novel, and significant, although I might have liked to see more detail and certainly more discussion; for example, about Fig. S6 which illustrates that results for three- and four-layer models are similar to the original six-layer model (now briefly described on page 6). That particular result strikes me as odd, as I might have considered a natural and more justified grouping to be the three-layer model, which separately distinguishes layer IV (a primarily input recipient and intrinsic layer). A main point, possibly problematic, is the focus on six-layers and the exclusion of agranular areas. Without further discussion and explication, this arguably detracts from the generality of the results. Some species have an exclusively pyramidalized cortex (as cetaceans; JM Graic... B. Cozzi, 2022, among others). Even within the six-layer scheme, as the authors will know, there are issues of sublamination, as well as significant intra-areal heterogeneity. For heterogeneity, there are well known examples based on callosal or acallosal zones, and topographic specializations (foveal vs. peripheral field representatiuon in V1) among others, Increasingly, areas are acknowledged to have significant intra-areal heterogeneity ( e.g., D. Haenelt... Tootell...2023 https://elifesciences.org/articles/78756 for myelin; E.M. Gordon et al., https://pubmed.ncbi.nlm.nih.gov/37076628/ 2023 for parallel processing (but: motor cortex); J. Gomez, Zhen, Weiner https://pubmed.ncbi.nlm.nih.gov/34618233/ 2021 for transcriptomics and visual field eccentricity, but relevant for laminar thickness comparisons). Can the Authors comment on how intra-areal heterogeneity may influence their results and their interpretation of inter-area interactions? If they are relying on an averaging approach, more careful description of the resulting strengths and limitations would be helpful, even in the Results, since this seems an important point. Similarly, it would be interesting to have more comparison with other approaches, such as results from receptor distribution, which might seem at first glance to give a different picture of organization.

In comparison with the core results on laminar thickness, the correlations with genetic expression and developmental trajectories are more derived from the literature and can seem more appropriate as speculations, although often presented as fact. In my opinion, the treatment of cell types was too broad and not well supported (Table S1), and the comparison with gene expression was necessarily inferential. I also had some reservations about the relationship of the lamination results to "processing hierarchy." First, the concept and details of "hierarchy" are still very much in discussion and even contentious, as the Authors briefly state (page 8). They reference articles 59-63, but more current articles should be included; for example, Hilgetag and Goulas, 2020, Vezoli et al., 2021, and (comparing the structural model and distance model) Aparicio-Rodriguez and Garcia-Cabezas, 2023. The Authors evidently elect for the Structural Model, but further justification or at least fuller discussion of the several alternatives would be appropriate. Second, the Authors frequently talk about "likelihood and strength of connections," but this also can seem inferential, given the non-straightforward homologies between human and macaque brains and again, the complexities of "strength." Similarly, page 8 (and elsewhere): "Feedforward connections transmit high-dimensional information..." [maybe better: are thought to transmit....]. The Authors may precisely be hoping to derive a predictive framework from their approach, but normal histology data are not adequate, and the extrapolated data from gene expression, development, and monkey tract tracing in my opinion, are not compelling. [ on organization of default mode network: CM Garin et al (2022) https://doi.org/10.1016/j.celrep.2022.110669, among others. ]

In summary, my recommendation is for more detailed emphasis on and discussion of the laminar thickness experimental data. Possible correlations with gene expression, and developmental trajectory might be better in a single section on "Speculations" or "Perspective" or "Future Directions." These are not in any case mentioned in the present title, although the Authors may have been aiming for a coherent and comprehensive framework.

The various analysis programs have been developed or extensively used by this Group or colleagues. Statistical analysis is important for this study, but I do not include this in my evaluation.

Other points:

1) The sulcal-gyral deformation is referred to as The "Bok principle" (1929), but the deformation was already illustrated by Economo and Koskinas (figure 30, 1925), possibly among others. 

2) The cellular descriptors, top of page 2, seemed unconventional. The densely packed "granular cells" in layer II are mainly pyramidal, and not necessarily small; layer III is commonly described as having a size gradient from IIIa (small pyramids) to IIIc (larger pyramids), layer IV outside the primary areas has small pyramidal cells (e.g., R. Douglas et al. in Shepherd "The Synaptic Organization..." 2004, 5th edition: "the spiny stellate neurons are found exclusively in layer 4 of the granular cortex" (referencing Cajal); J.S. Lund et al., 1981, from Golgi specimens in monkey visual cortex, and DeFelipe 2011, figures 4 and 13), layer V is often subdivided as Va (smaller, intratelencephalic pyramids) and Vb (with scattered large pyramids), and Layer VI is not usually described (?) has having "spindle-shaped neurons."

3) I may have overlooked this but are the data from both hemispheres? RH? LH? What about possible asymmetries?

4) Some statements are too sweeping; for example, bottom of p.8 "varying density of PV+ and CB+ inhibitory neuronal subtypes along cortical types is reported in macaque cortex." But ref. 23 refers only to prefrontal cortical systems. The statement needs to be better substantiated or further qualified (ex: DeFelipe...Elston, 1999 for macaque early visual areas).

Similarly, page 8 "from V1 to prefrontal cortex there is an increase in the density of dendritic spines." We do have a detailed comparison of monkey PFC and V1 (JI Luebke, 2017; JP Gilman et al., 2017) but that is a comparison only between two areas. The several relevant papers of Guy Elston are suggestive, but are also not comprehensive. In this regard, a relevant recent paper is Benavides-Piccione et al., 2021 https://pubmed.ncbi.nlm.nih.gov/33723567/

Similarly, page 8 " we observed that LTC G1 and the maps of cortical hierarchy were co-aligned with regional variability of several subtypes...." This is based on comparisons with the literature. Given the complexity of the putative results and the likely problems of extrapolations, this may be too sweeping.

Reviewer #2:

This work was based on the BigBrain histological atlas of a postmortem human brain. The authors first eliminated a-/dysgranular regions and to avoid the local effect of cortical curvature, they smoothed the laminar thickness using a moving disk. The normalized laminar thickness map was parcellated and the laminar thickness covariance (LTC) matrix was calculated. The LTC was generated by pairwise partial correlation of relative laminar thickness between cortical locations with Z-transformation, which allowed it to be viewed as a kind of 3D representation of the LTC matrix. PCA was applied to identify the main axes along the regional dissimilarity of laminar thickness. They mainly focused on the first PCA component (LTC G1) to extract the feature of the LTC matrix and showed the following characteristics.

First, LTC G1 was compared with asymmetry-based and laminar-based hierarchies, which were calculated as the group-averaged effective connective matrix and regional laminar-based hierarchy map of macaques, showing the correlation of r =0.39 and 0.54, respectively.

Second, they compared the LTC matrix with the structural connectivity (SC) and functional connectivity (FC) matrices averaged over a subset of the HCP data. SC likelihood and FC strength correlatedly increased with LTC, whereas they decreased with geodesic distance (GD). The SC matrix based on cortical thickness correlated with LTC, and the principal axis of SC correlated (r=0.55) with LTC G1. 

Third, the maturational coupling matrix was weakly correlated with the LTC matrix. They identified distinct sets of genes expressed at opposite ends of the LTC G1. That is: Rostral and caudal gene expressions are associated with mid-fetal/post-pubertal and fetal/pre-pubertal stages of human development, respectively. 

 Based on these analyses, the authors conclude the following in the Abstract and the first paragraph of the Discussion, which states that

1) They identified "a spatial pattern of changes in laminar thickness covariance from lateral frontal to posterior occipital regions, which differentiated the dominance of infra-versus supragranular layer thickness".

2) The infragranular-dominant pattern of laminar thickness was associated with higher hierarchical positions of regions, MRI based on resting-state effective connectivity in humans and tract- tracing based structural connections in macaques.

3) They show that regions with comparable laminar thickness patterns correspond to inter-regional structural covariance, mutational coupling, and transcriptome patterning, indicating developmental relevance.

4) They claim that in sum they characterize the association between organization of laminar thickness and processing hierarchy, anchored in ontology.

I agree with the authors on the first and second conclusions. However, I am less convinced about the third conclusion, particularly the developmental relevance from the actual data presented in this paper. My main concerns with the current form of the paper are with the last conclusion, which I feel they have not provided enough evidence to support it and the citations are not appropriate.

The following are issues related to my concerns in the text.

Page 2 (Abstract), lines 25-29: The fact that certain transcriptomic patterns coincide with layer thickness, structural covariance, and mature connectivity does not directly support an ontogenetic link between the organization of layer thickness and processing hierarchies.

Page 4 (Introduction), lines 78-80: The references cited are only partially relevant to the argument. Ref. 20 excludes the involvement of cortical thickness in the relationship to the connectivity property. Ref. 23 emphasizes more the a-/dysgranular layer in relation to PV vs. CB/CR within the PFC. Ref. 25 is an adult neurogenesis perspective and less relevant to the topic.

Page 4 line 82: Again, Ref. 25 is not relevant. Instead, See Rakic (1988), Science 241, 170-176 and Nadarajah, B. and Parnavelas, J.G. (2002) Nat. Rev. Neurosci. 3, 423-432, would be more relevant.

Page 4 lines 82-83. See Dehay, C. and Kennedy, H. (2007) Nat. Rev. Neurosci. 8, 438-450 and their original papers including the extended outer subventricular zone in non-human primates and in humans, e.g. by Hansen et al. Nature (2010) 464, 554-561, which can be cited.

Page 8 lines 209-211: They say "This suggests shared developmental/maturational and genetic (heritable) and environmental components". However, I see little "developmental", especially prenatal, evidence in the figure in Fig. 4a.

Page 8, line 225-Page 9, line 229: This is an interesting observation in itself. However, the existence of these two sets of genes adds little to what has already been reported in the field of developmental neurobiology.

Page 10, line 291- Page 11, line 293: This is too schematic a view. Please also refer to recent studies, e.g. Gao et al, Nature Neurosci., 27, 515-529, (2022); D'Souza et al, Nature Commun, (2022)13:503, and others as well. The authors' own discussion of these recent results is then required.

Page 11 line 310-311: It is not clear how the reference relates to the current study, since the difference between PV and CR in ref 23 is most different between a-/dysgranular and granular cells. However, in this paper, the authors exclude the former group from the analysis.

Minor comments

Page 5, line 122 (Fig. 1e): I am not sure what the difference is between the left and right pair of figures. Also, please clarify the relationship to Figs. S2 and S4.

Page 6, line 138 (Fig. S8): In Fig. S8a, "microstructural profile" should read "cortical depth" as shown in Ref. 10.

COMMENTS FROM THE ACADEMIC EDITOR:

[lightly edited]

I have had a quick look at the text and there are some problems in the discussion:

Ref 59 Bastos et al., 2012 is a theoretical review of predictive coding and is incorrectly cited on p10. In the same line Felleman and Van Essen 1991 and Rockland and Pandya 1979 are cited for laminar patterns of feedforward (FF) and feedback (FB) connections. These studies are relatively dated and should be completed by Barone..Kennedy 2000, and Markov et al., JCN 2014. The following 2 sentences are confusing, because they both imply that FB are limited to infragranular layers. That is not the case: throughout the extrastriate cortex of NHP there is a robust supragranular FB pathway! This is reported in Markov et al. JCN 2014 and discussed in Vezoli et al., NeuroImage 2021.

I found the whole paragraph at the top of P11 very confused and extremely biased in its citation of the literature. The authors citation 64 for functional is very strange , Michalareas et al., Neuron 2016 is a cortical hierarchy in human based on function which is much more to the point and relevant than ref 64. Finally the conclusion of the penultimate line of this paragraph is simply undefendable "that is, they influence the activity of lower regions rather than being influenced by them refs 65-67)" . This is a speculation based on nothing tangible, and should be removed.

The last line ;" The hierarchy in macaque was defined based on the laminar patters of feedforward and feedback connections identified in tract-tracing studies ref 39. The tract tracing studies in question were done by the Kennedy lab and the resulting hierarchy published in Markov JCN 2014 and then subsequently in more detail in Vezoli NeuroImage 2021. BTW the Vezoli et al 2021 is highly pertinent to the present study and should be cited.

The paragraph beginning line 319 makes much of the strength of connections and its consequence for understanding large-scale models of the cortex. The authors cite numerous papers looking at the issue uniquely from the perspective of the structural model and go out of the way to cite the alternative which is based on connectivity Ercsey-Ravasz et al., Neuron 2013, Markov et al., Science 2013. These studies from the Kennedy lab have shown that connectivity based models have strong predictability and again should be cited.

Finally, in the paragraph starting line 352 there is a long-winded attempt to relate adult structure to developmental processes. Here again the Kennedy lab has carried out numerous studies on showing the role of cortical neurogenesis on adult cytoarchitecture in rodents and NHP, this body of work including reviews and original studies none of which are cited. Instead they continue to cite their colleague Hilgetag (refs 23, 26 , 30) co-adapts of the structural model.

In light of the above I think there is a clear need for the authors to re-write the discussion and give a much more balanced view of the field.

# Author comment: Author response to Decision Letter 1

**DOI:** 10.1371/journal.pbio.3002365.r003

29 Aug 2023

Attachment Submitted filename: Response Letter.pdf Click here for additional data file.

# Peer review: Decision Letter 2

**DOI:** 10.1371/journal.pbio.3002365.r004

**Authors:** Roland G Roberts

22 Sep 2023

Dear Dr Valk,

Thank you for your patience while we considered your revised manuscript "The spatial arrangement of laminar thickness profiles in the human isocortex scaffolds cortical organization" for publication as a Short Reports at PLOS Biology. This revised version of your manuscript has been evaluated by the PLOS Biology editors, the Academic Editor and both of the original reviewers.

Based on the reviews, we are likely to accept this manuscript for publication, provided you satisfactorily address the remaining points raised by the reviewers, and other policy-related requests.

IMPORTANT - please attend to the following:

a) The use of the word "scaffold" in your Title is potentially confusing (because it is more normally a noun) and we believe that it implies a causal relationship that is not demonstrated here. Instead, please could you change it to something like the following: "The regional variation of laminar thickness in the human isocortex is related to cortical hierarchy and inter-regional connectivity"

b) Please attend to the remaining requests from the reviewers. The Academic Editor has asked me to say that s/he disagrees with reviewer #1's request to remove the discussion of development and non-human primates, finding these discussions to be "both useful and pertinent." The Academic Editor also emphasised reviewer #2's suggestion to mention externopyramidization is a good one, as are this reviewer's comments about Layer IV, which should be taken into account.

c) Many thanks for supplying your data and code in Github and the container app in Docker. However, because Github depositions can be readily changed or deleted, please make a permanent DOI’d copy (e.g. in Zenodo) and provide this URL (see below).

d) Please cite the location of the data/code clearly in all relevant main and supplementary Figure legends, e.g. “The data and code needed to generate this Figure can be found in https://doi.org/10.5281/zenodo.XXXXX”

As you address these items, please take this last chance to review your reference list to ensure that it is complete and correct. If you have cited papers that have been retracted, please include the rationale for doing so in the manuscript text, or remove these references and replace them with relevant current references. Any changes to the reference list should be mentioned in the cover letter that accompanies your revised manuscript.

We expect to receive your revised manuscript within two weeks. 

To submit your revision, please go to https://www.editorialmanager.com/pbiology/ and log in as an Author. Click the link labelled 'Submissions Needing Revision' to find your submission record. Your revised submission must include the following:

- a cover letter that should detail your responses to any editorial requests, if applicable, and whether changes have been made to the reference list

- a Response to Reviewers file that provides a detailed response to the reviewers' comments (if applicable)

- a track-changes file indicating any changes that you have made to the manuscript. 

NOTE: If Supporting Information files are included with your article, note that these are not copyedited and will be published as they are submitted. Please ensure that these files are legible and of high quality (at least 300 dpi) in an easily accessible file format. For this reason, please be aware that any references listed in an SI file will not be indexed. For more information, see our Supporting Information guidelines:

https://journals.plos.org/plosbiology/s/supporting-information

*Published Peer Review History*

Please note that you may have the opportunity to make the peer review history publicly available. The record will include editor decision letters (with reviews) and your responses to reviewer comments. If eligible, we will contact you to opt in or out. Please see here for more details:

https://blogs.plos.org/plos/2019/05/plos-journals-now-open-for-published-peer-review/

*Press*

Should you, your institution's press office or the journal office choose to press release your paper, please ensure you have opted out of Early Article Posting on the submission form. We ask that you notify us as soon as possible if you or your institution is planning to press release the article.

*Protocols deposition*

To enhance the reproducibility of your results, we recommend that if applicable you deposit your laboratory protocols in protocols.io, where a protocol can be assigned its own identifier (DOI) such that it can be cited independently in the future. Additionally, PLOS ONE offers an option for publishing peer-reviewed Lab Protocol articles, which describe protocols hosted on protocols.io. Read more information on sharing protocols at https://plos.org/protocols?utm_medium=editorial-email&utm_source=authorletters&utm_campaign=protocols

Please do not hesitate to contact me should you have any questions.

Sincerely,

Roli Roberts

Roland Roberts, PhD

Senior Editor,

rroberts@plos.org,

PLOS Biology

------------------------------------------------------------------------

DATA NOT SHOWN?

- Please note that per journal policy, we do not allow the mention of "data not shown", "personal communication", "manuscript in preparation" or other references to data that is not publicly available or contained within this manuscript. Please either remove mention of these data or provide figures presenting the results and the data underlying the figure(s).

------------------------------------------------------------------------

REVIEWERS' COMMENTS:

Reviewer #1:

The Authors appear to have made extensive revisions, and include detailed point-by-point responses to previous comments. On closer reading and comparison of R2 and R1, however, I found the revisions to be less extensive than at first glance, and ended up with many of the same misgivings that I had with the original manuscript. 

A main concern, echoing Reviewer 2, is the attempt to correlate findings on laminar thickness with development (dialogue, page 7, 8). 1) The developmental epochs given (bottom of Response page 8) are broad: "mid-fetal and post-pubertal," "late fetal and pre-pubertal." There is a huge literature on developmental events and timetables, as the Authors will know. Obviously, further detail would detract even more from the primary focus of the present paper and might be best addressed in a separate publication. However, it can seem superficial not to incorporate (compare, not only just cite) the many relevant results of established researchers in that field such as Kostovic, Molnar, and Huttner among others. My opinion remains that the sections on development do not reinforce, but rather detract from the main findings and conclusions. Similar comment about reworded paragraph in the Discussion (page 10 of Response). The results can stand alone, and are better focused without the developmental distraction.

I might make the same comment about the link to macaque connectivity, which also can appear superficial and has in any case been previously emphasized by this Group. 

The use of feedback/feedforward beyond the early sensory pathways remains problematic (why discard Maunsell and Felleman "lateral?"). The Author Response, (pages 19, and 20 in reference to Discussion page 14) seems minimal - just to note (in passing?) the existence of "additional patterns." This is arguably disingenuous, and not a clear or useful correction. Key papers in visual cortex are still missing: A. Angelucci and colleagues (2006, 2020, 2021 among others); and S.Shen...Tolias (2022) on "...two cortico-cortical feedback pathways." There is also a 2022 Frontiers Research Topic "Feed-forward and Feedback Processing...." (comments by E. Zagha; K. Rockland; among others). Likely, there is not space, in a Short Report, to adequately discuss the relevant points, and the best option may be to curtail or postpone discussion here, or refer to previous relevant pubs where this is already raised by the present Authors.

In summary, this could be an interesting and focused addition to the several papers from this Group on cortical laminar thickness. The correlations with development and macaque connectivity detract from the core findings, especially in what is a Short Report, and obscure what can be an interesting approach to deal with complex issues of lamination and area organization.

If there is space, "limitations" section (as in Wagstyl et al., 2020) would be useful, as perhaps also a brief section on "future directions."

Reviewer #2:

The authors have largely addressed my concerns. I have only a few comments to add. 

1. In the second paragraph of the Discussion, the authors cite references 69, 70, 71, 72 published by Barbara Finlay's laboratory. The series of studies by Finlay focus on layers II-VI, and layers V-VI as a separate set for cross-validation of rostral and caudal cortical gradients across mammalian species. The Materials and Methods section of Reference 69 clearly states, "Measurements of layers were taken orthogonal to the cortical surface area. In our samples, it was difficult to reliably distinguish layers II and III. Moreover, layer IV could not be reliably visualized in some parts of the isocortex (e.g., agranular cortex). In order to systematically compare neuron numbers in various layers across the RC and ML axes of the iso-cortex, we combined layers II-III and IV in our analyses. Layer V and VI were analyzed together. We therefore only examine how supragranular (layer II-IV), infragranular (layers V-VI) neurons, and their sum vary across the RC and ML axes of the isocortex". Thus, they did not distinguish layer IV from layers II and III. In contrast, in the current study, layer IV is the key layer that characterizes the BigBrain separately from the supragranular layers of layers II and III. In the immediately following sentence the authors state: "In an integrated model of combined laminar thickness and intensity variations, we observed a similar rostral to caudal principal axis characterizing increased gray matter density in all layers, most prominently in layer IV". I think this is not a logical smooth flow.

2. Externopyramidization may be related to the expansion of the outer subventricular zone (Dehay & Kennedy, 2007; Smart, I. H. M. et al., Cereb. Cortex 12, 37-53, 2002). The authors cite references 113 and 114, but do not specifically mention it in the discussion.

3. A minor comment. There are some mixed styles in the figure legends. Fig. 1: small letters for numbering (a, b, c, with bold and non-bold letters), Fig. 2: capital letters for numbering.

# Author comment: Author response to Decision Letter 2

**DOI:** 10.1371/journal.pbio.3002365.r005

6 Oct 2023

Attachment Submitted filename: Response Letter.pdf Click here for additional data file.

# Editor report: Decision Letter 3

**DOI:** 10.1371/journal.pbio.3002365.r006

**Authors:** Roland G Roberts

6 Oct 2023

Dear Dr Valk,

Thank you for the submission of your revised Short Report "The regional variation of laminar thickness in the human isocortex is related to cortical hierarchy and inter-regional connectivity" for publication in PLOS Biology. On behalf of my colleagues and the Academic Editor, Henry Kennedy, I'm pleased to say that we can in principle accept your manuscript for publication, provided you address any remaining formatting and reporting issues. These will be detailed in an email you should receive within 2-3 business days from our colleagues in the journal operations team; no action is required from you until then. Please note that we will not be able to formally accept your manuscript and schedule it for publication until you have completed any requested changes.

Please take a minute to log into Editorial Manager at http://www.editorialmanager.com/pbiology/, click the "Update My Information" link at the top of the page, and update your user information to ensure an efficient production process.

PRESS: We frequently collaborate with press offices. If your institution or institutions have a press office, please notify them about your upcoming paper at this point, to enable them to help maximise its impact. If the press office is planning to promote your findings, we would be grateful if they could coordinate with biologypress@plos.org. If you have previously opted in to the early version process, we ask that you notify us immediately of any press plans so that we may opt out on your behalf.

We also ask that you take this opportunity to read our Embargo Policy regarding the discussion, promotion and media coverage of work that is yet to be published by PLOS. As your manuscript is not yet published, it is bound by the conditions of our Embargo Policy. Please be aware that this policy is in place both to ensure that any press coverage of your article is fully substantiated and to provide a direct link between such coverage and the published work. For full details of our Embargo Policy, please visit http://www.plos.org/about/media-inquiries/embargo-policy/.

Thank you again for choosing PLOS Biology for publication and supporting Open Access publishing. We look forward to publishing your study. 

Sincerely, 

Roli Roberts

Roland G Roberts, PhD, PhD

Senior Editor

PLOS Biology

rroberts@plos.org
